# In vitro PK/PD modeling of tyrosine kinase inhibitors in non‐small cell lung cancer cell lines

**DOI:** 10.1111/cts.13714

**Published:** 2024-03-13

**Authors:** Linda Wanika, Neil D. Evans, Martin Johnson, Helen Tomkinson, Michael J. Chappell

**Affiliations:** ^1^ School of Engineering University of Warwick Coventry UK; ^2^ MSD London UK; ^3^ Limina Clinical Pharmacology Warrington UK

## Abstract

Tyrosine kinase inhibitors (TKIs) are routinely prescribed for the treatment of non‐small cell lung cancer (NSCLC). As with all medications, patients can experience adverse events due to TKIs. Unfortunately, the relationship between many TKIs and the occurrence of certain adverse events remains unclear. There are limited in vivo studies which focus on TKIs and their effects on different regulation pathways. Many in vitro studies, however, that investigate the effects of TKIs observe additional changes, such as changes in gene activations or protein expressions. These studies could potentially help to gain greater understanding of the mechanisms for TKI induced adverse events. However, in order to utilize these pathways in a pharmacokinetic/pharmacodynamic (PK/PD) framework, an in vitro PK/PD model needs to be developed, in order to characterize the effects of TKIs in NSCLC cell lines. Through the use of ordinary differential equations, cell viability data and nonlinear mixed effects modeling, an in vitro TKI PK/PD model was developed with estimated PK and PD parameter values for the TKIs alectinib, crizotinib, erlotinib, and gefitinib. The relative standard errors for the population parameters are all less than 25%. The inclusion of random effects enabled the model to predict individual parameter values which provided a closer fit to the observed response. It is hoped that this model can be extended to include in vitro data of certain pathways that may potentially be linked with adverse events and provide a better understanding of TKI‐induced adverse events.

**Study Highlights**

**WHAT IS THE CURRENT KNOWLEDGE ON THE TOPIC?**

Knowledge of pathways between tyrosine kinase inhibitors (TKIs) and adverse events is often limited. In addition to this, there are limited human data available that focus on TKIs and different gene expressions and pathways. This limitation reduces the ability to construct potential adverse event pathways. However, there are many in vitro studies which do observe the changes in different gene expressions due to TKIs.

**WHAT QUESTION DID THIS STUDY ADDRESS?**

The development of an in vitro TKI model based on publicly available data and the application of nonlinear mixed effects modeling.

**WHAT DOES THIS STUDY ADD TO OUR KNOWLEDGE?**

With the aid of cell viability data and nonlinear mixed effects modeling, parameter estimates were obtained for an in vitro pharmacokinetic/pharmacodynamic model for the TKIs alectinib, crizotinib, erlotinib, and gefitinib.

**HOW MIGHT THIS CHANGE CLINICAL PHARMACOLOGY OR TRANSLATIONAL SCIENCE?**

This model can be further extended to model other potential pathways for adverse events using key observations made in in vitro studies.

## INTRODUCTION

Targeted therapy in non‐small cell lung cancer (NSCLC), has provided many patients with NSCLC with improved prognosis and survivability.^1^, ^2^ Tyrosine kinase inhibitors (TKIs) are one of the main classes of drugs that are used as a targeted therapy for NSCLC treatment.^2^ TKIs target receptor tyrosine kinases (RTKs), in an effort to reduce tumor cell proliferation, angiogenesis, and reduce the size of the tumor.^3^, ^4^, ^5^ Common examples of TKIs include erlotinib and gefitinib.

As with all medications, there is a chance that patients may experience adverse events with a TKI therapy. Serious adverse events may become life‐threatening, as the adverse event begins to interfere with their physiological system.^6^ Suspension of NSCLC treatment may also occur with serious adverse event diagnosis.^7^ This can provide an opportunity for the tumor to grow and potentially spread to other regions. Unfortunately, patients may also die due to a serious adverse event.^6^

For many adverse events, the mechanisms are unclear.^8^, ^9^ There is a need to investigate potential adverse event mechanisms, as many patients are affected by adverse events with unexplained mechanisms worldwide.^10^, ^11^, ^12^

There are limited in vivo studies that investigate the effects of TKIs on different protein expressions or the activation of certain genes. Most of the in vivo studies focus on tumor response to the TKIs. As for adverse event studies, many are observational analyses where certain biomarkers or laboratory measurements are taken prior to the treatment and are continuously recorded until an adverse event occurs.^13^, ^14^, ^15^ Although these studies are useful, it is often difficult to interpret how these factors could potentially be related to the adverse event.

Many in vitro studies, however, analyze different phosphorylated protein expressions and changes in gene expression.^16^, ^17^, ^18^, ^19^ These studies could provide potential insights into the mechanisms for different adverse events. Analysis on the phosphorylation pathways of RTKs can enable a model to be developed to simulate the activation or deactivation of certain growth factors which have been shown to be related to pulmonary adverse events.^20^ The developed in vitro model can then be transformed by considering a tumor compartment to generate an in vivo model to simulate the possible mechanism for pulmonary adverse events due to TKIs. In order to utilize these studies, it is imperative to capture the transition from when the drug is introduced to the medium and all the way to any effects the drug could have on the potential targets. The pharmacokinetic (PK) relationship among TKIs, NSCLC cells, and the cell medium remains limited.

Nonlinear mixed effects (NLME) modeling enables PK and pharmacodynamic (PD) parameter values to be estimated from an observed population.^21^ The use of in vitro data and NLME modeling will allow an in vitro PK/PD model to be developed for TKIs in NSCLC cell lines. This model can then potentially be expanded to simulate potential pathways for adverse events.

The aim of this paper is to develop an in vitro PK/PD model for TKIs in NSCLC cell lines with the use of in vitro data and NLME modeling. The Methods section will cover the data collection, model development (including the structural identifiability and sensitivity analysis), and the incorporation of NLME into the structural model. In the Results section, the sensitivity analysis plot is displayed along with the parameter estimates and model simulations. These results are then summarized and discussed in the Discussion section.

## METHODS

### In vitro data collection

In vitro data were extracted from cell viability studies that observed the changes in tumor cells' viability in response to TKIs over time. Studies were included if the following details were present in the study: TKI concentrations versus time, duration of the treatment, number of cells that were placed in each well, and the name of NSCLC cell lines that were used. TKI groups that had less than 10 viability analyses were removed and the treatment duration was also limited to 72 h only. The decisions for these additional criteria to be included were made to provide adequate populations for the NLME modeling and 72 h was the most common treatment duration. As a result, cell viability for the following TKIs were collected; alectinib (120 cell‐viability observations), crizotinib (129 cell‐viability observations), erlotinib (61 cell‐viability observations), and gefitinib (82 cell‐viability observations). WebPlotDigitizer was used to extrapolate the tumor cell viability response, the minimum concentration to produce 50% inhibition (IC_50_), the maximum inhibition response (*I*_MAX_), and in cases where there were extra TKI dosages, the TKI concentration.^22^

Electron micrographs of each of the cell lines that were included were used to estimate the initial cell volume which was based on sphere volume calculations. The image software tool ImageJ was used to estimate the cell volumes.^23^ The in vitro tumor cell viability data as well as the cell volumes that were estimated, along with their reference sources, can be found in Tables S1‐S5 in the Supplementary Materials.

### Structural model in vitro TKI PK/PD model

**FIGURE 1:**
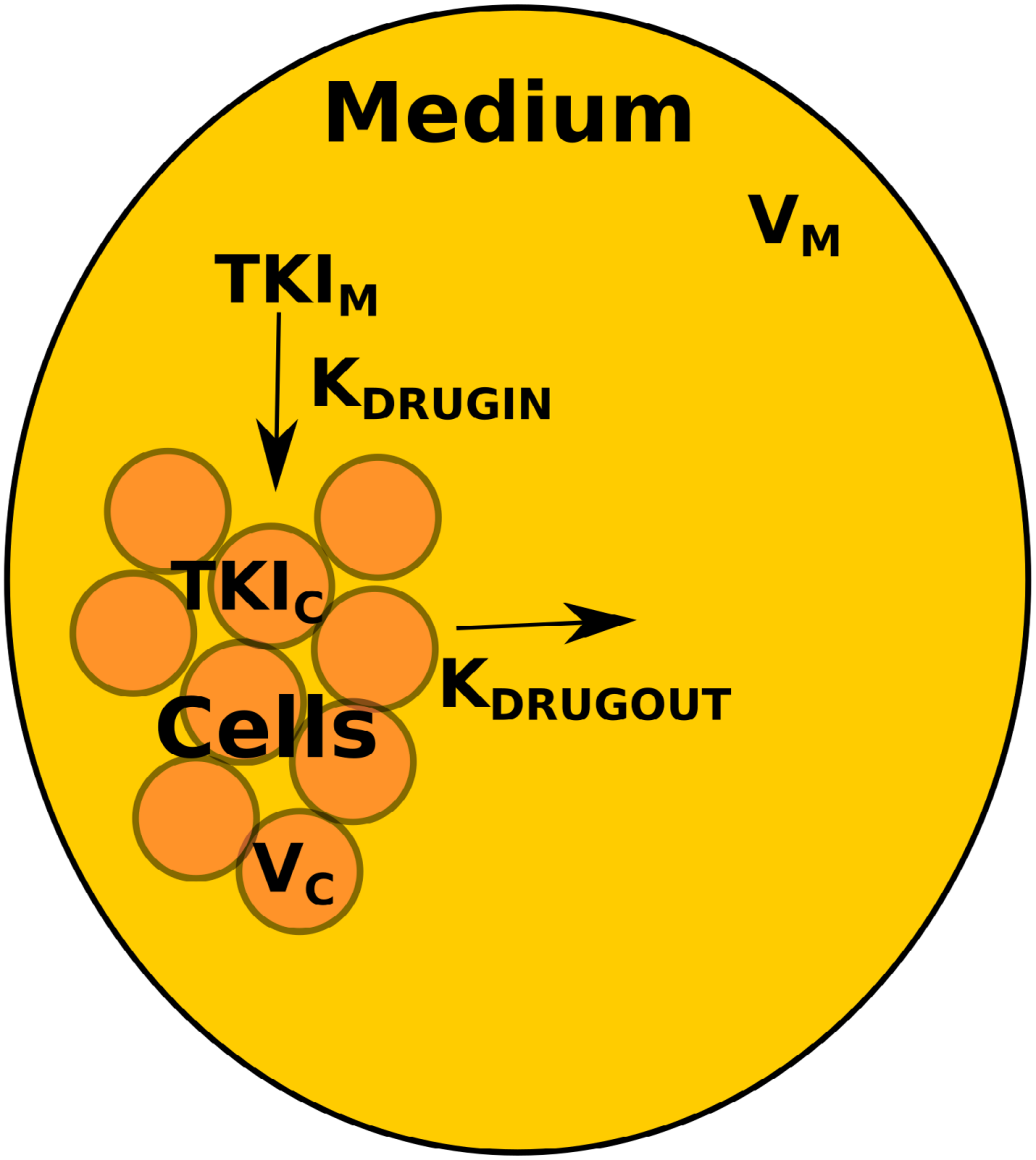
Pharmacokinetic process in an in vitro setting. TKI_C_ and TKI_*M*_ refers to the concentration of TKI in the cell and medium respectively. *K*_DRUGIN_ and *K*_DRUGOUT_ refers to the absorption rate of TKI form the medium into the cell and from the cell back into the medium respectively. *V*_*C*_ and *V*_*M*_ refer to the volume of the cell and medium, respectively. TKI, tyrosine kinase inhibitor.

In an in vitro setting, the main state variables for the PK process are the concentrations of the drug in the medium and the cell. Figure 1 provides a simplified schematic diagram of the PK process in an in vitro setting. Although the TKIs will produce their effects in cells, the TKIs are first introduced into the medium, thus the absorption process from the medium into the cell has to be captured. In Figure 1, the only parameters that are known are the initial concentration of the TKI in the medium and the initial volume of the cell. Note that, in this case, the volume of the cell refers to all the cells that are grouped together in a well.

The following ordinary differential equations (ODEs) characterize the changes in concentration of TKI in the medium and in the cell:

(1) $$\frac{d\left({\mathrm{TKI}}_{M}\right)}{\mathrm{dt}}=-\left(K_{\text{DRUGIN}}\times {\mathrm{TKI}}_{M}\right)+\left(\frac{K_{\text{DRUGOUT}}}{\frac{V_{M}}{V_{C\left(t\right)}}}\right)\times {\mathrm{TKI}}_{C}$$

(2) $$\frac{d\left({\mathrm{TKI}}_{C}\right)}{\mathrm{dt}}=\left(\frac{K_{\text{DRUGIN}}}{\frac{V_{C\left(t\right)}}{V_{M}}}\right)\times {\mathrm{TKI}}_{M}-\left(K_{\text{DRUGOUT}}\times {\mathrm{TKI}}_{C}\right)$$

The definitions for the parameters for all of the equations as well as their respective units can be found in Table 1. Note that the time “(*t*)” has been dropped for all the state variables for the sake of brevity.

**TABLE 1:** Definitions for all of the parameters and state variables.

States and parameters	Definitions	Initial values
TKI_*M*_ (*0*)	TKI concentration in the cell medium	Dose (μmol/L)
TKI_*C*_ (*0*)	TKI concentration in the cell	0 (μmol/L)
TumorCellViability (*0*)	Tumor cell viability	100 (%)
*K*_DRUGIN_ (h^−1^)	TKI absorption rate from medium into cell	
*K*_DRUGOUT_ (h^−1^)	TKI absorption rate from cell back into the medium	
*V*_*M*_ (L)	Volume of the medium	
*V*_*C(0)*_ (L)	Initial cell volume	
*K*_IN_ (h^−1^)	Tumor cell proliferation rate	
*I*_MAX_ (%)	Maximum tumor cell viability inhibition	
IC_*50*_ (μmol/L)	TKI concentration required to produce 50% tumor cell viability inhibition	
*K*_OUT_ (h^−1^)	Tumor cell decay rate	

Abbreviation: TKI, tyrosine kinase inhibitor.

One of the main effects of TKIs is the reduction of the tumor size, which would reduce the NSCLC cell volume. This reduction in volume would affect the concentration of the TKI in the cell. Therefore, the volume of the cell at time *t*, *V*_*C(t)*_, can be described as:

(3) $$V_{C\left(t\right)}=V_{C\left(0\right)}\times \frac{\text{TumorCellViability}\;\left(t\right)}{\text{TumorCellViability}\;\left(0\right)}$$

The PDs of TKIs in NSCLC cell lines can also be represented by a first order ODE.^24^ A first order indirect PD model was chosen to take into account the impact of TKI concentration on the cell viability and also to generate an appropriate dose response curve. The main effect of TKIs on NSCLC cell lines that is observed in cell viability studies is a reduction in cell viability which reduces the cell volume, due to inhibition of phosphorylated RTKs.^25^ Equation 4 was derived to characterize the change in tumor cell viability as a result of TKI administration, through the use of an indirect effect compartment model.^26^

(4) $$\frac{d\;\left(\text{TumorCellViability}\right)}{\mathrm{dt}}=K_{\text{IN}}\times \left(1-\frac{I_{\mathrm{MAX}}\times {\mathrm{TKI}}_{C}}{{\mathrm{IC}}_{50}+{\mathrm{TKI}}_{C}}\right)-K_{\text{OUT}}\times \text{TumorCellViability}\;$$

Note that for this system of ODEs, only the parameters *V*_*C*(0)_, *I*_MAX_, and IC_50_ are known. Moreover, TumorCellViability is the only state that is observed. Although the volume of the medium is not known, the volume ratio, $V_{R}$, between the cell volume and cell medium, *V*_*M*_, is known (between 1 and 8).^27^ Hence, *V*_*M*_ can be represented as:

(5) $$V_{M}=V_{C\left(0\right)}\times V_{R}$$

### Structural identifiability analysis

A structural identifiability analysis assesses whether the unknown parameters of a model can be identified uniquely or otherwise based on the structural model and the states of the model that are observed. Consider the following as a generalized ODE model formulation:

(6) $$\left\{\begin{array}{c} \frac{dx(t)}{dt}=f\left(x\left(t\right),\mu ,u\left(t\right)\right)\\ y\left(t\right)=g\left(x\left(t\right),\mu ,u\left(t\right)\right)\\ x\left(0\right)=x^{\mathrm{INT}} \end{array}\right.$$

where $dx_{i}\left(t\right)/dt$ denotes the rate of change of the states of the ODE. The function $f\left(x\left(t\right),\mu ,u\left(t\right)\right)$ encompasses the ODE dynamics for the vector with elements ($x\left(t\right)$), μ (which represents a vector of unobserved and unknown parameters), and $u\left(t\right)$, the input vector. The $y\left(t\right)$ represents the output and is considered as a smooth function with the same elements as the system states. Last, x(0) represents the vector of initial conditions for the states ($x^{\mathrm{INT}}$).

A specific parameter, μ_*i*_, is said to be globally identifiable if for all of *t*, the following condition is met:

(7) $$y\left(t,\hat{\mu }\right)=y\left(t,\mu^{*}\right)\Rightarrow {\hat{\mu }}_{i}={\mu_{i}}^{*}$$

In other words, if μ_*i*_ can be uniquely determined from $y\left(t\right)$, then μ_*i*_ is globally identifiable. Therefore, μ_*i*_ can still be classed as locally identifiable if for μ_*i*_ there exists a neighborhood, $V\left(\mu^{*}\right)$ where Equation 7 holds. However, if μ_*i*_ cannot be classed as either globally or locally identifiable, then μ_*i*_ is said to be unidentifiable.^28^, ^29^, ^30^, ^31^

For a model to be classed as at‐least locally identifiable, none of the parameters can be deemed as unidentifiable. Subsequent numerical estimations of unidentifiable parameters will not be reliable as there are infinitely many values that an unidentifiable parameter can take for the given observation.

In this study, the input–output relation and Taylor series approaches were used to assess the structural identifiability of the system. The Taylor series method utilizes the Taylor series coefficients of the observed output, to assess for structural identifiability.^30^, ^31^ This study utilizes the Lie derivatives of the observed output to generate the input–output relation. The coefficients of the monomials that were generated from the input–output relation approach are then used to assess for the structural identifiability of the model. These coefficients (Taylor series and input–output relations) include some (or in some cases all) of the parameters that are in the ODE system. These coefficients are also assumed to be equal to unique solutions, which can be used to identify whether a parameter is identifiable. Both methods were used to overcome the issues with computational memory limitations and model complexity.^32^ Based on the model ODEs and knowledge that the parameters *V*_*C*(0)_, *I*_MAX_, and IC_50_ are known, the model is deemed as locally identifiable. A detailed analysis of the structural identifiability of this model in an NLME context can be found in the supplementary materials. The Taylor series approach was performed in *MATHEMATICA* and the input–output approach was performed in *MAPLE*.^33^, ^34^

### Sensitivity analysis

Sensitivity analysis effectively analyses the impact that changes in the model parameters will have on the model output.^35^ This is useful for validating whether the model is truly characterizing the system that the model aims to represent. Moreover, for models that have many parameters, it can aid in the identification of parameters which could potentially be removed from the model, reducing its parameter complexity which may additionally improve the structural identifiability of the model.

Sensitivity analysis was performed on the model presented using the Sobol sensitivity indices method.^36^ This global sensitivity analysis method is based on analyzing the variance of the output state based on different parameter values. The variance is then decomposed into different contribution scores for each of the parameters and their impact on the output's variance. Any parameter which had a Sobol value of 0 would indicate that this particular parameter has no impact to the output of a model. The sensitivity analysis was performed on the software tool *R* using the packages *ODEsensitivity* and *ODEnetwork*.^37^, ^38^, ^39^

### Nonlinear mixed effects modeling

The NLME modeling comprises of both the population effects and the random effects. The population effects refer to the population parameter estimates, whereas, in this study, the random effects refer to the between subject variability (or interindividual variability) as well as the residual unknown variability (RUV).^21^ The general NLME predicted output form is given by:

(8) $$Y_{\mathrm{ij}}=f\left(x_{\mathrm{ij}},\psi_{i}\right)+g\left(x_{\mathrm{ij}},\psi_{i},\xi \right)\times \varepsilon_{\mathrm{ij}}$$

where *i* represents the individuals in the population and *j* represents the number of observations. The $x_{\mathrm{ij}}$ refers to the states in the model ODEs that describe the state for individual *i* and observation *j*. The $\psi_{i}$ refers to the individual estimated parameter values. The $g\left(x_{\mathrm{ij}},\psi_{i},\xi \right)\times \varepsilon_{\mathrm{ij}}$ represents the residual unknown variability. In this study, *MONOLIX* was used to perform the NLME modeling.^40^ *MONOLIX* utilizes the stochastic approximation expectation maximization algorithm in order to estimate population parameter values.^41^ In short, individual parameter values are drawn from a conditional probability which contains population parameter estimates that are set by the user. These individual parameter values are then assessed for the likelihood of being able to produce the observed output. New population estimates are then formed based on these individual parameter values and the process repeats until the estimated parameter values begin to converge.

## RESULTS

### Sensitivity analysis

**FIGURE 2:**
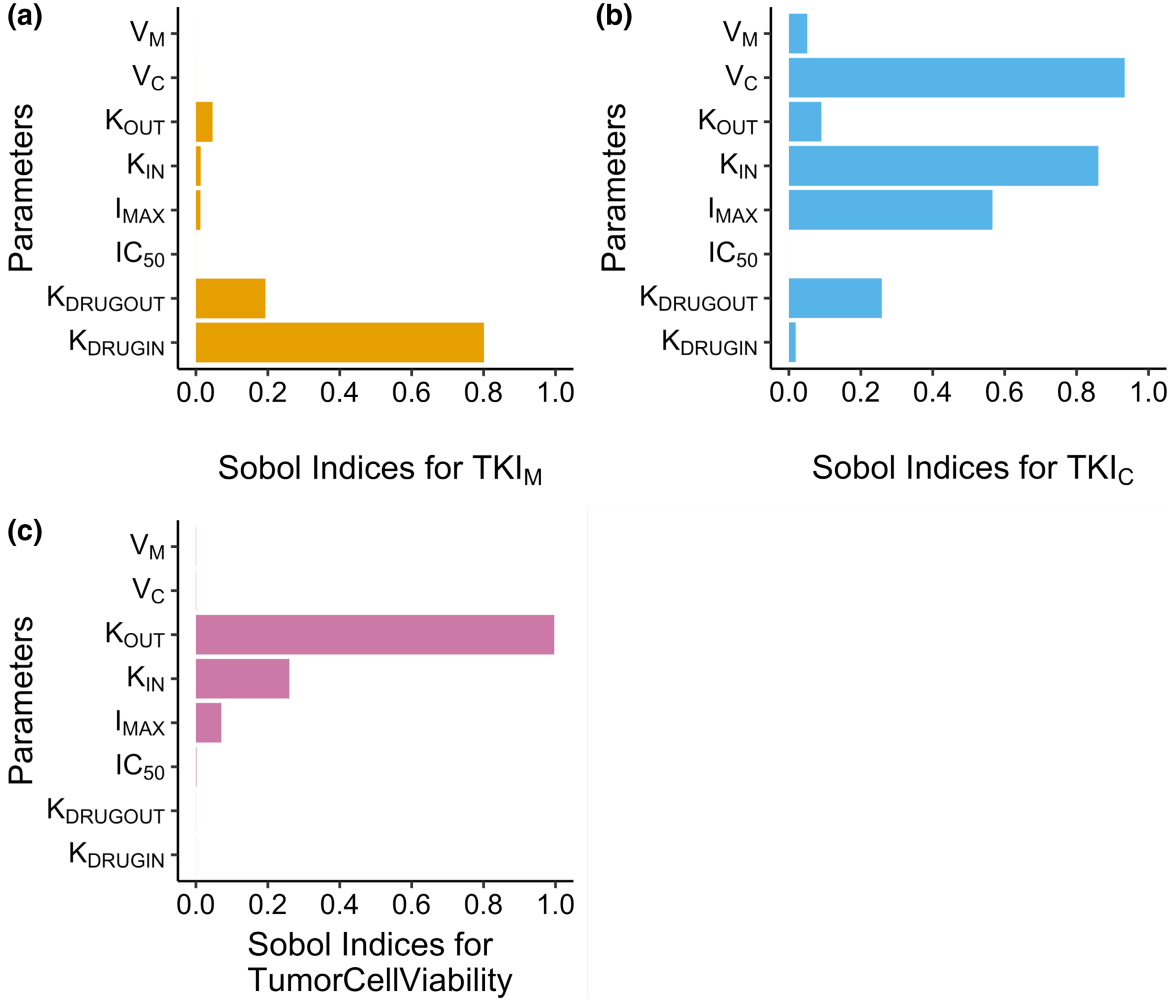
Sobol indices for the parameters in the model. (a) Sobol indices with respect to *TKI*_*M*_. (b) Sobol indices with respect to *TKI*_*C*_. (c) Sobol indices with respect to *TumorCellViability*. IC_50_, half‐maximal inhibitory concentration; *I*_max_, maximum inhibition; TKI, tyrosine kinase inhibitor.

Figure 2 visualizes the Sobol indices for each of the parameters in the model.

In Figure 2a, *K*_DRUGIN_ has the highest Sobol indices at 0.8. Other parameters that have Sobol indices greater than 0 include *K*_OUT_, *K*_IN_, and *I*_MAX_. For *TKI*_*C*_ (Figure 2b), *V*_*C*_, *K*_IN_, *I*_MAX_, *K*_OUT_, and *K*_DRUGOUT_ have higher Sobol indices compared to the other parameters. Last, for the observed output state (tumor cell viability), *K*_OUT_ has the highest Sobol indices of ~1. Other parameters with high Sobol indices are *K*_IN_ and *I*_MAX_ (Figure 2c).

### Parameter estimation

For each of the analyses, a constant error model was chosen as the best model to characterize the RUV. Equation 8 can therefore be represented as:

(9) $$Y_{\mathrm{ij}}=f\left(x_{\mathrm{ij}},\psi_{i}\right)+a\times \varepsilon_{\mathrm{ij}}$$

where *a* is a constant value (see Table 2) and $\varepsilon_{\mathrm{ij}}$ often represents a normal distribution with a mean 0 and variance of 1. Other error models were assessed, such as the proportional and combined error model. However, the constant error model obtained the lowest standard errors and relative standard errors (RSEs).

**TABLE 2:** Parameter estimates for the in vitro PK/PD models.

Parameters	TKIs						
	Mean	SE	RSE	Variance	SE	RSE (%)	Shrinkage (%)
**Alectinib**							
*K*_DRUGIN_ (h^−1^)	0.745	0.049	6.619	0.231	0.056	24.312	100
*K*_DRUGOUT_ (h^−1^)	0.096	0.017	17.536	0.112	0.471	420.116	100
*V*_*R*_	4.051	0.405	9.990	0.0348	0.434	1248.549	100
*K*_IN_ (h^−1^)	1.061	0.153	14.384	0.411	0.105	25.669	14.6
*K*_OUT_ (h^−1^)	0.021	0.002	10.353	0.082	0.294	356.845	92.5
*a*	16.196	1.032	6.370				
**Crizotinib**							
*K*_DRUGIN_ (h^−1^)	0.672	0.041	6.104	0.164	0.087	52.954	99.5
*K*_DRUGOUT_ (h^−*1*^)	0.298	0.052	17.460	0.052	1.414	2730.637	99.4
*V*_*R*_	4.234	0.951	22.452	0.100	1.544	1550.351	99.6
*K*_IN_ (h^−1^)	1.126	0.122	10.827	0.205	0.121	59.011	29.2
*K*_OUT_ (h^−1^)	0.026	0.003	12.737	0.931	0.076	8.151	−0.751
*a*	0.0136	0.001	6.226				
**Erlotinib**							
*K*_DRUGIN_ (h^−1^)	0.987	0.035	3.526	0.185	0.026	14.127	7.48
*K*_DRUGOUT_ (h^−1^)	0.051	0.011	22.590	0.362	0.212	58.553	28.4
*V*_*R*_	3.739	0.278	7.429	0.178	0.070	39.040	8.95
*K*_IN_ (h^−1^)	2.925	0.132	4.517	0.067	0.0384	57.353	10.4
*K*_OUT_ (h^−1^)	0.033	0.003	8.379	0.328	0.061	18.624	−1.1
*A*	0.003	0.000	9.054				
**Gefitinib**							
*K*_DRUGIN_ (h^−1^)	0.856	0.034	3.927	0.113	0.039	34.224	100
*K*_DRUGOUT_ (h^−1^)	0.313	0.055	17.533	0.355	0.196	55.268	100
*V*_*R*_	3.192	0.496	15.527	0.198	0.193	97.754	100
*K*_IN_ (h^−1^)	0.941	0.042	4.420	0.068	0.042	61.307	99.3
*K*_OUT_ (h^−1^)	0.034	0.004	10.693	0.760	0.077	10.113	−0.90
*A*	0.737	0.058	7.807				

*Note*: “a” corresponds to the (residual unknown variability) element which, in this case, is represented as a constant error model. Shrinkage values are based on the empirical Bayes' estimates.

Abbreviations: RSE, relative standard error; SE, standard error; TKI, tyrosine kinase inhibitor.

From Equation 5, *V*_*R*_ can be used to estimate *V*_*M*_. All of the estimates for *V*_*R*_ lie in the 1–8 range (Table 2). For all of the TKIs, the estimated values for *K*_DRUGIN_ are higher than the estimated value for *K*_DRUGOUT_. The model for the alectinib group has the lowest *K*_OUT_ value (0.021), whereas that for crizotinib has the highest parameter estimate value for *V*_*R*_, whereas the erlotinib estimates yield the highest *K*_IN_ value, as well as lower RSE values for the random effects compared to the other TKIs. The gefitinib estimates have the lowest population estimate for the parameter *K*_IN_. Overall, the erlotinib parameter estimates have the lowest shrinkage compared to those for the other TKIs.

### Prediction versus observation

**FIGURE 3:**
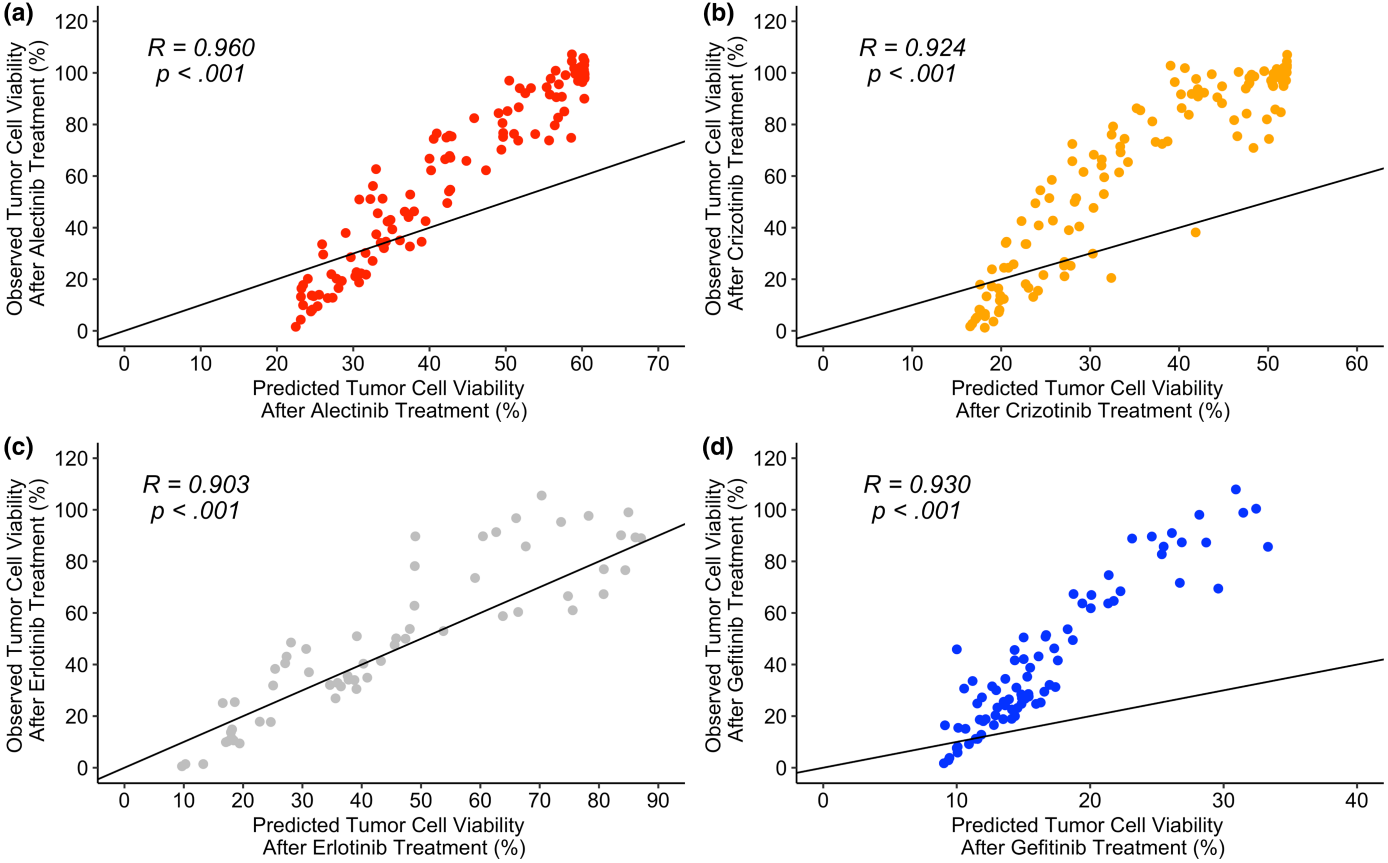
Predictions versus observations for tumor cell viability using population estimates only. (a) Alectinib model. (b) Crizotinib model. (c) Erlotinib model. (d) Gefitinib model. *R* refers to the correlation value with the *p* value (denoted as “*p*”). the *p* values for the alectinib, crizotinib, erlotinib, and gefitinib treatments are, respectively, as follows: 2.331 × 10^−67^, 1.021 × 10^−54^, 2.949 × 10^−23^, and 1.658 × 10^−36^.

**FIGURE 4:**
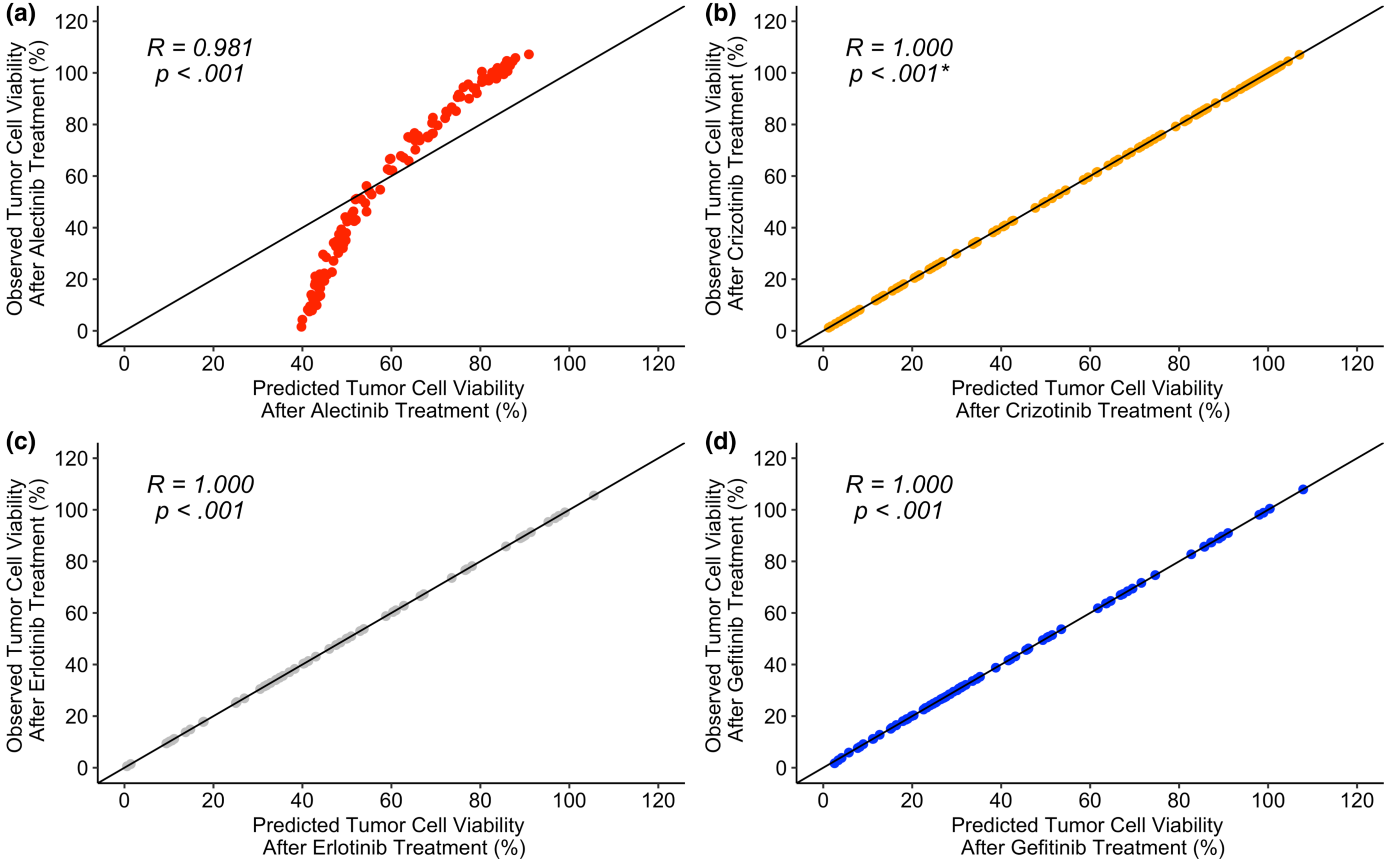
Predictions versus observations for tumor cell viability using individual parameter estimates. (a) Alectinib model. (b) Crizotinib model. (c) Erlotinib model. (d) Gefitinib model. The *p* values for the alectinib, crizotinib, erlotinib, and gefitinib treatments are, respectively, as follows: 1.068 × 10^−85^, 0*, 5.897 × 10^−169^, and 1.478 × 10^−184^. *Note that a value of 0 does not show that a value is zero but rather that the value is smaller than the minimum value allowed to be computed in *R*.

Figures 3 and 4 showcase the model predictions versus the observed data using the population estimates only (Figure 3) and the inclusion on random effects (Figure 4).

In Figure 3c, the model parameter estimates for the erlotinib group only using the population estimates only has the widest range for the predicted values between 0 and 90%. The other TKI groups in Figure 3 show a restriction between 0 and 70% or, in, the case of the gefitinib, only 0 and 40% (Figure 3d). For all of the plots, the observed tumor cell viability can reach above 100%.

Figure 4a, shows that the alectinib individual parameter estimates were still constrained to lie within 40 to 90% of tumor cell viability. The other TKI groups (Figure 4b‐d), have predicted cell tumor viability ranges that were the same as for the observed tumor cell viability ranges. Moreover, the crizotinib, erlotinib, and gefitinib groups each achieved a correlation value of 1.

### Simulated dose response curve

**FIGURE 5:**
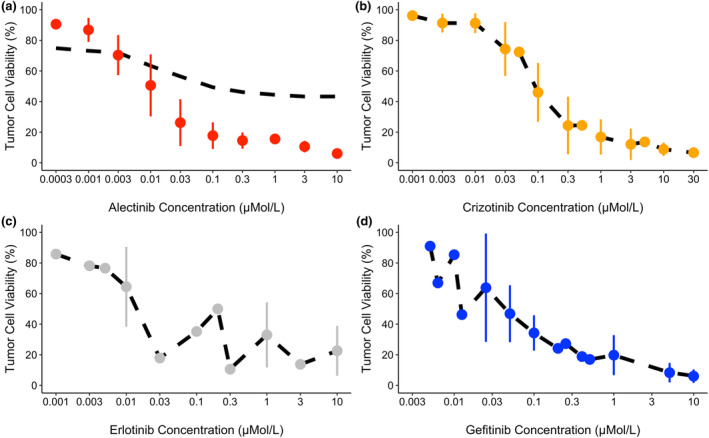
Simulated dose response curves for tumor cell viability using individual parameter estimates. (a) Alectinib model. (b) Crizotinib model. (c) Erlotinib model. (d) Gefitinib model. The black dashed line represents the model simulation, and the circles represent the observed values. Vertical lines represent the standard deviation ranges. For the alectinib and crizotinib models, the simulation was based on H3122 cells. For the erlotinib model, this was based on HCC827 cells and for the gefitinib model this was based on PC9 cells.

Figure 5 visualizes the simulated dose response curves based on the parameter estimates.

The specific cell lines were chosen as these cell lines had the most varied TKI concentrations and the most data associated with them. Similar to the previous plots, the alectinib simulation response lies between 50% and 80% of tumor cell viability (Figure 5a). The crizotinib observed response is the only plot which shows a gradual decrease in cell growth due to the increase in crizotinib concentration (Figure 5b). Both the erlotinib and gefitinib plots show that the average observed values for the NSCLC cell lines HCC827 and PC9 (respectively) can fluctuate as the concentration increases. The simulated dose response curve using only the population parameter estimates only can be found in Figure S1 in the Supplementary Materials.

## DISCUSSION

The sensitivity analysis for the parameter TKI_*M*_ (Figure 2a) shows that parameters *K*_DRUGIN_ and *K*_DRUGOUT_ are the most influential parameters. In Equation 1, both the volumes enter into the denominators for the latter part of the ODE, which implies that, unless the volumes are extremely small, the numerator (including the parameter *K*_DRUGOUT_) will have more of an impact. As for the parameter *K*_DRUGIN_, it is the only parameter that is multiplied by TKI_*M*_ in Equation 1. *K*_OUT_, *K*_IN_, and *I*_MAX_ are also seen as important parameters for TKI_*M*_ (Figure 2a). These parameters are also influential for TKI_*C*_ and tumor cell viability (Figure 2b,c). The state TKI_*C*_ also influences TKI_*M*_. The concentration of TKI in the cell is also greatly influenced by the volume of the cell which is affected by the tumor cell viability, where the parameters *K*_OUT_, *K*_IN_, and *I*_MAX_ have significant influence. The IC_50_ parameter, however, does not seem to influence any of the states to a notable effect. This may be due to the fact that, based on Equation 4, the *I*_MAX_ value always impacts on the tumor cell viability more than IC_50_. The parameters estimated are not dependent on the NSCLC cell line mutation status. This decision was made to reflect the fact that the IC_50_ and *I*_MAX_ values would reflect any mutation that was present. Although many PK/PD models often fix the *I*_MAX_ value at 1,^42^ the decision to include the individual *I*_MAX_ values was to aid the model to provide enhance characterization of the data that were observed.

All of the models predicted higher *K*_IN_ rates compared to the *K*_OUT_ rates, which are in line with what is known about tumors in their actively proliferating state. In the majority of cases, the *K*_DRUGOUT_ and *V*_*R*_ estimates had the highest RSE values. With the exception of the erlotinib parameter estimates, the shrinkage for the estimates for *K*_DRUGIN_, *K*_DRUGOUT_, and *V*_*R*_ are above 99%, which suggests that the individual parameter estimates will likely be similar to the population parameter estimates. The shrinkage values for the *K*_OUT_ estimates were lower for the crizotinib, erlotinib, and gefitinib estimates when compared to the other parameter shrinkage values. This indicates that there is less uncertainty with respect to the individual parameter estimates for *K*_OUT_. Given that *K*_OUT_ has significant influence on the tumor cell viability (Figure 2c), the individual estimates for *K*_OUT_ have an impact on the prediction versus observation response when individual estimates are included.

For the predictions versus observations plots (Figures 3 and 4), the alectinib plots have restricted ranges which could possibly be due to the restrictions placed on the *I*_MAX_ values, as discussed previously. In Figure 4, the crizotinib, erlotinib, and gefitinib plots all achieved a correlation value of 1 suggesting that the inclusion of random effects enables the model to capture the observed data efficiently. The shrinkage values for the alectinib parameter estimates were above 90% for all of the parameter estimates except for *K*_IN_, which suggests that the individual estimates were similar to the population parameter estimates. This could explain why, even with the inclusion of individual estimates, the prediction versus observation plot for alectinib (Figure 4a), remained similar to that for the population estimates only (Figure 3a). Moreover, this suggests that perhaps there are not enough data for the model to be able to fully predict the PK and PD processes for alectinib. Nonetheless, all of the plots had high positive correlation values of at least 0.9 which suggests that the model's assumed relationship between concentration and tumor viability inhibition is similar to the observed data.

In Figure 5, the average simulated dose response curves for most of the plots show that the inclusion of the random effects provided a closer fit for the model when compared to the data. In the alectinib plot (Figure 5a), the simulated response remains at 50% of tumor cell viability whereas the observed response actually decreases to less than 20%. This outcome may be due to the low *K*_OUT_ value compared to other TKI estimates (Table 2). Although the crizotinib model also has a similar predicted *K*_OUT_ value (0.026), the variance is a lot higher than compared to the alectinib variance for *K*_OUT_, which could suggest that other individual *K*_OUT_ values were higher, enabling a more accurate prediction. For the alectinib variance for *K*_OUT_ (0.082), this leads to the assumption that individual *K*_OUT_ estimates will most likely remain close to their population value. This could impede the ability of the predictions to be lower for the higher concentrations. Moreover, even though the *I*_MAX_ values for the H3122 NSCLC cell line were on average 0.9, Figure 2 shows that the most influential parameter for tumor cell viability response is actually K_OUT_. For Figure 5c,d, the tumor cell viability fluctuates as the concentration of the TKIs increases. Whereas the plots are based on the one specific cell line and specific concentrations, the data originate from different studies which have different cell viability response values at the same TKI concentrations. The model was able to capture the fluctuations as the individual estimates are based on the individual studies, from which the mean was taken to produce the simulated response.

This in vitro model shares many similarities with its in vivo model counterpart. Higher *K*_DRUGIN_ rates for erlotinib and gefitinib compared to the other TKIs are also present in in vivo studies.^43^, ^44^, ^45^, ^46^ The tumor cell volume is also considered, which for many models also incorporates this as part of the effect compartment.^47^

The next step for this model is to include the phosphorylation cascade in order to incorporate target growth factors that have been linked to pulmonary adverse events, such as interstitial lung disease (ILD). Common approaches for predicting ILD involve the use of artificial intelligence (AI).^48^, ^49^ However, although these approaches can achieve high prediction scores, there is limited information on why certain risk factors may lead to an increased likelihood of ILD. This unfortunately further confounds understanding of the mechanism behind ILD.^50^ The extended in vitro PK/PD model, includes the potential mechanistic pathway of ILD (based on the phosphorylation data from pulmonary cells). When the PD elements of the in vitro model are incorporated into a patient model, the different TKI medications as well as dosing regimens can be implemented and the likelihood of ILD onset will be mechanistically based on the TKI and dosing regimen for each patient.

The main limitations in this analysis to note are that the observations and predictions are based on data that were extracted digitally and thus are only an estimate and that the data obtained are based on different in vitro studies. Even in cases where the same NSCLC cell lines were used, there may still be differences in the experimental and growth conditions and proliferation rates. These differences will also impact on the *I*_*MAX*_ and IC_50_ values as well as the model parameter estimates. The estimates obtained through NLME modeling are dependent on the data used. Due to these confounding factors and the sparsity of the available data, the inclusion of more data would be necessary for the alectinib, crizotinib, and gefitinib models to be utilized in a robust predictive capacity. However, the erlotinib model demonstrates that it is feasible to capture the PK/PD process based on the population estimates alone (Figure 3c). This specific model could potentially be further explored to incorporate the RTK pathway and simulate the possible mechanism(s) for adverse events.

In addition to this, the in vitro model does not include other factors, such as protein binding, the number of receptors available, active TKI metabolites, the diffusion rate from the cell membrane to the cell nucleus, etc. The main reason for these exclusions was to reduce the complexity of the model and for the model to be structurally identifiable. To conclude this study, an in vitro TKI PK/PD model was developed with the use of NLME modeling. It is believed that this is the first instance where estimates for these parameters for these TKIs in an in vitro setting have been obtained without prior experimentation for data collection. It is hoped that this model can be furthered developed in order to model other potential pathways for adverse events.

## AUTHOR CONTRIBUTIONS

L.W., N.D.E., and M.J.C. wrote the manuscript. L.W., N.D.E., M.J., H.T., and M.J.C. designed the research. L.W. performed the research. L.W. analyzed the data.

## FUNDING INFORMATION

This project has been funded by the EPSRC grant award in collaboration with AstraZeneca (REF: 2038176).

## CONFLICT OF INTEREST STATEMENT

The authors declared no competing interests for this work.

## Supporting information


Data S1

## References

[1] Yuan M , Huang LL , Chen JH , Wu J , Xu Q . The emerging treatment landscape of targeted therapy in non‐small‐cell lung cancer. Sig Transduct Target Ther. 2019;4:61. 10.1038/s41392-019-0099-9 PMC6914774 31871778

[2] Chan BA , Hughes BG . Targeted therapy for non‐small cell lung cancer: current standards and the promise of the future. Transl Lung Cancer Res. 2015;4(1):36‐54. 25806345 10.3978/j.issn.2218-6751.2014.05.01 PMC4367711

[3] Hall Richard D , Le Tri M , Haggstrom Daniel E , Gentzler Ryan D . Angiogenesis inhibition as a therapeutic strategy in non‐small cell lung cancer (NSCLC). Transl Lung Cancer Res. 2015;4(5):515‐523. 26629420 10.3978/j.issn.2218-6751.2015.06.09 PMC4630516

[4] Franklin W , Veve R , Hirsch F , Helfrich B , Bunn P . Epidermal growth factor receptor family in lung cancer and premalignancy. Semin Oncol. 2002;29(1):3‐14. 10.1053/sonc.2002.31520 11894009

[5] Saraon P , Pathmanathan S , Snider J , Lyakisheva A , Wong V , Staglijar I . Receptor tyrosine kinases and cancer: oncogenic mechanisms and therapeutic approaches. Oncogene. 2021;40:4079‐4093. 34079087 10.1038/s41388-021-01841-2

[6] Cancer Therapy Evaluation Program . Protocol development website. Accessed 15 June, 2023. https://ctep.cancer.gov/protocoldevelopment/electronic_applications/ctc.htm

[7] Ota S , Matsukawa T , Yamamoto S , et al. Severe adverse events by tyrosine kinase inhibitors decrease survival rates in patients with newly diagnosed chronic‐phase chronic myeloid leukemia. Eur J Haematol. 2018;101(1):95‐105. 29660177 10.1111/ejh.13081

[8] Nawa H , Niimura T , Hamano H , et al. Evaluation of potential complications of interstitial lung disease associated with antiandrogens using data from databases reporting spontaneous adverse effects. Front Pharmacol. 2021;12:655605. 34177574 10.3389/fphar.2021.655605 PMC8220081

[9] Rana P , Aleo MD , Wen X , Kogut S . Hepatotoxicity reports in the FDA adverse event reporting system database: a comparison of drugs that cause injury via mitochondrial or other mechanisms. Acta Pharm. Sin. B. 2021;11(12):3857‐3868. 35024312 10.1016/j.apsb.2021.05.028 PMC8727782

[10] Schwendimann R , Blatter C , Dhaini S , Simon M , Ausserhofer D . The occurrence, types, consequences and preventability of in‐hospital adverse events – a scoping review. BMC Health Serv Res. 2018;18(1):521. 29973258 10.1186/s12913-018-3335-z PMC6032777

[11] Santomasso BD , Nastoupil LJ , Adkins S , et al. Management of immune‐related adverse events in patients treated with chimeric antigen receptor T‐cell therapy: ASCO guideline. J Clin Oncol. 2021;39(35):3978‐3992. 34724386 10.1200/JCO.21.01992

[12] Kim M , Yang YS , Ko Y , Choi M . Major adverse events in patients with peripheral artery disease after endovascular revascularization: a retrospective study. J Clin Med. 2022;11(9):2547. 35566674 10.3390/jcm11092547 PMC9102344

[13] Kawase S , Hattori N , Ishikawa N , et al. Change in serum KL‐6 level from baseline is useful for predicting life‐threatening EGFR‐TKIs induced interstitial lung disease. Respir Res. 2011;12:97. 21791074 10.1186/1465-9921-12-97 PMC3160959

[14] Valeyrie L , Bastuji‐Garin S , Revuz J , et al. Adverse cutaneous reactions to imatinib (STI571) in Philadelphia chromosome‐positive leukemias: a prospective study of 54 patients. J Am Acad Dermatol. 2003;48(2):201‐206. 12582389 10.1067/mjd.2003.44

[15] Lenihan DJ , Kowey PR . Overview and management of cardiac adverse events associated with tyrosine kinase inhibitors. Oncologist. 2013;18(8):900‐908. 23918069 10.1634/theoncologist.2012-0466 PMC3755926

[16] Kogita A , Togashi Y , Hayashi H , et al. Activated MET acts as a salvage signal after treatment with alectinib, a selective ALK inhibitor, in ALK‐positive non‐small cell lung cancer. Int J Oncol. 2014;46(3):1025‐1030. 25502629 10.3892/ijo.2014.2797

[17] Zhang X , Liu G , Kang Y , Dong Z , Qian Q , Ma X . N‐cadherin expression is associated with acquisition of EMT phenotype and with enhanced invasion in Erlotinib‐resistant lung cancer cell lines. PloS One. 2013;8(3):e57692. 23520479 10.1371/journal.pone.0057692 PMC3592915

[18] National Center Biotechnology Information (NCBI) . GEO, Series GSE116442 website. Accessed 15 June, 2023. https://www.ncbi.nlm.nih.gov/geo/query/acc.cgi?acc=GSE116442

[19] National Center Biotechnology Information (NCBI) . GEO, Series GSE34228 website. Accessed 15 June, 2023. https://www.ncbi.nlm.nih.gov/geo/query/acc.cgi?acc=GSE34228

[20] Saito A , Horie M , Nagase T . TGF‐β signaling in lung health and disease. Int J Mol Sci. 2018;19(8):2460. 30127261 10.3390/ijms19082460 PMC6121238

[21] Salahudeen MS , Nishtala PS . An overview of pharmacodynamic modelling, ligand‐binding approach and its application in clinical practice. Saudi Pharm J. 2017;25(2):165‐175. 28344466 10.1016/j.jsps.2016.07.002 PMC5355565

[22] Rohatgi A . Web Plot Digitizer website. Accessed 15 June, 2023. https://automeris.io/WebPlotDigitizer

[23] Schneider CA , Rasband WS , Eliceiri KW . NIH image to ImageJ: 25 years of image analysis. Nat Methods. 2012;9(7):671‐675. 22930834 10.1038/nmeth.2089 PMC5554542

[24] Nykamp D . An introduction to ordinary differential equations ‐ Math Insight website. Accessed 15 June, 2023. https://mathinsight.org/ordinary_differential_equation_ introduction

[25] Metibemu DS , Akinloye OA , Akamo AJ , Ojo DO , Okeowo OT , Omotuyi IO . Exploring receptor tyrosine kinases‐inhibitors in cancer treatments. Egypt J Med Hum Genet. 2019;20(1):20‐35.

[26] Dayneka NL , Garg V , Jusko WJ . Comparison of four basic models of indirect pharmacodynamic responses. J Pharmacokinet Biopharm. 1993;21(4):457‐478. 8133465 10.1007/BF01061691 PMC4207304

[27] Thermofisher . Useful numbers for cell culture. Thermo Fisher Scientific – UK website. Accessed 15 June, 2023. https://www.thermofisher.com/uk/en/home/references/gibco‐cell‐culture‐basics/cell‐culture‐protocols/cell‐culture‐useful‐numbers.html

[28] Yates J , Evans N , Chappell M . Structural identifiability analysis via symmetries of differential equations. Automatica. 2009;45(11):2585‐2591.

[29] Janzén D , Jirstrand M , Chappell M , Evans N . Three novel approaches to structural identifiability analysis in mixed‐effects models. Comput Methods Programs Biomed. 2019;171:141‐152. 27181677 10.1016/j.cmpb.2016.04.024

[30] Janzén D , Jirstrand M , Chappell M , Evans N . Extending existing structural identifiability analysis methods to mixed‐effects models. Math Biosci. 2018;295:1‐10. 29107004 10.1016/j.mbs.2017.10.009

[31] Bearup D , Evans N , Chappell M . The input‐output relationship approach to structural Identifiability analysis. UKACC International Conference on CONTROL 2010. 10.1016/j.cmpb.2012.10.012 23228562

[32] Chis O , Banga J , Balsa‐Canto E . Structural Identifiability of systems biology models: a critical comparison of methods. PloS One. 2011;6(11):e27755. 22132135 10.1371/journal.pone.0027755 PMC3222653

[33] Wolfram Research, Inc . Mathematica, Version 13.0.0, Champaign, IL. 2021.

[34] Monagan MB , Geddes KO , Heal KM , Labahn G , Vorkoetter S , Devitt J . Maple V Programming Guide: For Release 5. Springer Science & Business Media. Accessed: July 2022. https://www.maplesoft.com/products/Maple/; 2012.

[35] Jarrett AM , Liu Y , Cogan NG , Hussaini MY . Global sensitivity analysis used to interpret biological experimental results. J Math Biol. 2015;71:151‐170. 25059426 10.1007/s00285-014-0818-3

[36] Nossent J , Elsen P , Bauwens W . Sobol’ sensitivity analysis of a complex environmental model. Environ Model Softw. 2011;26(12):1515‐1525.

[37] R Core Team . R: A Language and Environment for Statistical Computing. R Foundation for Statistical Computing; 2022. website. Accessed 15 June, 2023. https://www.R‐project.org/

[38] Weber F , Theers S . ODEsensitivity: Sensitivity analysis of ordinary differential equations. R package version 1.1.2 website. Accessed 15 June, 2023. https://CRAN.R‐project.org/package=ODEsensitivity

[39] Surmann D . ODEnetwork: Network of differential equations_ R package version 1.3.2 website. Accessed 15 June, 2023. https://CRAN.R‐project.org/package=ODEnetwork

[40] Monolix version 2019R1. Antony, France: Lixoft SAS website. Accessed 15 June, 2023. http://lixoft.com/products/monolix/

[41] Lixoft, Monolix methodology version 4.3.3 website. Accessed 15 June, 2023. http://lixoft.com/wp‐content/ uploads/2016/03/monolixMethodology.pdf

[42] Gabrielsson J , Andersson R , Jirstrand M , Hjorth S . Dose‐response‐time data analysis: an underexploited trinity. Pharmacol Rev. 2018;71(1):89‐122. 10.1124/pr.118.015750 30587536

[43] TARCEVA . Accessdata.fda.gov website. (erlotinib) FDA. Accessed 15 June, 2023. https://www.accessdata.fda.gov/drugsatfda_docs/label/2010/021743s14s16lbl.pdf

[44] Gefitinib FDA . Accessdata***.fda.gov website. Accessed 15 June, 2023. https://www.accessdata.fda.gov/drugsatfda_docs/nda/2015/206995orig1s000clinpharmr.pdf

[45] ALENCESA . Accessdata***.fda.gov website. (alectinib) FDA. Accessed 15 June, 2023. https://www.accessdata.fda.gov/drugsatfda_docs/label/2016/208434s001lbl.pdf

[46] XALKORI . Accessdata***.fda.gov website. (crizotinib) FDA. Accessed 15 June, 2023. https://www.accessdata.fda.gov/drugsatfda_docs/label/2021/202570s030lbl.pdf

[47] Eigenmann MJ , Frances N , Hoffmann G , Lavé T , Walz A . Combining nonclinical experiments with translational PKPD modeling to differentiate erlotinib and gefitinib. Mol Cancer Ther. 2016;15(12):3110‐3119. 27638857 10.1158/1535-7163.MCT-16-0076

[48] Xu W , Wu W , Zhang D , et al. A novel CT scoring method predicts the prognosis of interstitial lung disease associated with anti‐MDA5 positive dermatomyositis. Sci Rep. 2021;11:17070. 34426622 10.1038/s41598-021-96292-w PMC8382835

[49] Exarchos KP , Gkrepi G , Kostikas K , Gogali A . Recent advances of artificial intelligence applications in interstitial lung diseases. Diagnostics. 2023;13(13):2303. 37443696 10.3390/diagnostics13132303 PMC10340709

[50] Zhang J , Qiu T , Zhou Y , Wu S , Chen E . Tyrosine kinase inhibitors‐associated interstitial lung disease used in non‐small cell lung cancer: a pharmacovigilance analysis based on the FDA adverse event reporting system database. Expert Opin Drug Saf. 2023;22(9):849‐856. 37026465 10.1080/14740338.2023.2193392

