# Supplementary material for: In vitro PK/PD modeling of tyrosine kinase inhibitors in non‐small cell lung cancer cell lines
Source: Clin Transl Sci. 2024 Mar 13;17(3):e13714. doi: 10.1111/cts.13714 (PMC10933606; doi:10.1111/cts.13714)
Supplement: Supplementary file 1 — Data S1 [file CTS-17-e13714-s001.docx]

**Dose response curves based on population estimates only**


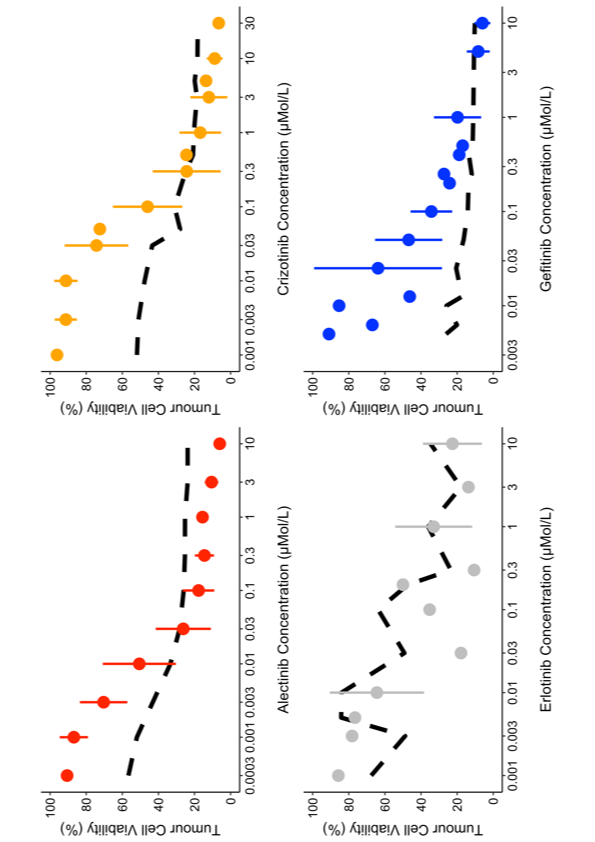


Figure S1: (a) alectinib model. (b) crizotinib model. (c) erlotinib model. (d) gefitinib model. The black dashed line represents the model simulation, circles represent the observed values. Vertical lines represent the standard deviation ranges. For the alectinib and crizotinib models the simulation was based on H3122 cells. For the erlotinib model this was based on HCC827 cells and for the gefitinib model this was based on PC9 cells.

**Table S1: Tumour cell viability data for alectinib**

| ID | Cell Line | Conc (μM) | Cell Viability (%) | Time (hr) | *IC_50_* (μM) | *I_MAX_* | *V_C(0)_* (L) | Ref |
| --- | --- | --- | --- | --- | --- | --- | --- | --- |
| 1 | H3122 | 0.0003 | 100 | 0 | 0.0102 | 0.95675676 | 6.43E-09 | 1 |
| 1 | H3122 | 0.0003 | 90.578755 | 72 | 0.0102 | 0.95675676 | 6.43E-09 | 1 |
| 2 | H3122 | 0.001 | 100 | 0 | 0.0102 | 0.95675676 | 6.43E-09 | 1 |
| 2 | H3122 | 0.001 | 85.1988259 | 72 | 0.0102 | 0.95675676 | 6.43E-09 | 1 |
| 3 | H3122 | 0.003 | 100 | 0 | 0.0102 | 0.95675676 | 6.43E-09 | 1 |
| 3 | H3122 | 0.003 | 74.4151529 | 72 | 0.0102 | 0.95675676 | 6.43E-09 | 1 |
| 4 | H3122 | 0.01 | 100 | 0 | 0.0102 | 0.95675676 | 6.43E-09 | 1 |
| 4 | H3122 | 0.01 | 50.9265618 | 72 | 0.0102 | 0.95675676 | 6.43E-09 | 1 |
| 5 | H3122 | 0.03 | 100 | 0 | 0.0102 | 0.95675676 | 6.43E-09 | 1 |
| 5 | H3122 | 0.03 | 29.6001329 | 72 | 0.0102 | 0.95675676 | 6.43E-09 | 1 |
| 6 | H3122 | 0.1 | 100 | 0 | 0.0102 | 0.95675676 | 6.43E-09 | 1 |
| 6 | H3122 | 0.1 | 20.1678113 | 72 | 0.0102 | 0.95675676 | 6.43E-09 | 1 |
| 7 | H3122 | 0.3 | 100 | 0 | 0.0102 | 0.95675676 | 6.43E-09 | 1 |
| 7 | H3122 | 0.3 | 17.7603013 | 72 | 0.0102 | 0.95675676 | 6.43E-09 | 1 |
| 8 | H3122 | 1 | 100 | 0 | 0.0102 | 0.95675676 | 6.43E-09 | 1 |
| 8 | H3122 | 1 | 16.4360877 | 72 | 0.0102 | 0.95675676 | 6.43E-09 | 1 |
| 9 | H3122 | 3 | 100 | 0 | 0.0102 | 0.95675676 | 6.43E-09 | 1 |
| 9 | H3122 | 3 | 13.2188746 | 72 | 0.0102 | 0.95675676 | 6.43E-09 | 1 |
| 10 | H3122 | 10 | 100 | 0 | 0.0102 | 0.95675676 | 6.43E-09 | 1 |
| 10 | H3122 | 10 | 4.32432432 | 72 | 0.0102 | 0.95675676 | 6.43E-09 | 1 |
| 11 | A549 | 0.0003 | 100 | 0 | 5.52 | 0.67296744 | 1.27E-08 | 1 |
| 11 | A549 | 0.0003 | 98.1435534 | 72 | 5.52 | 0.67296744 | 1.27E-08 | 1 |
| 12 | A549 | 0.001 | 100 | 0 | 5.52 | 0.67296744 | 1.27E-08 | 1 |
| 12 | A549 | 0.001 | 104.658839 | 72 | 5.52 | 0.67296744 | 1.27E-08 | 1 |
| 13 | A549 | 0.003 | 100 | 0 | 5.52 | 0.67296744 | 1.27E-08 | 1 |
| 13 | A549 | 0.003 | 103.061032 | 72 | 5.52 | 0.67296744 | 1.27E-08 | 1 |
| 14 | A549 | 0.01 | 100 | 0 | 5.52 | 0.67296744 | 1.27E-08 | 1 |
| 14 | A549 | 0.01 | 105.790873 | 72 | 5.52 | 0.67296744 | 1.27E-08 | 1 |
| 15 | A549 | 0.03 | 100 | 0 | 5.52 | 0.67296744 | 1.27E-08 | 1 |
| 15 | A549 | 0.03 | 102.30339 | 72 | 5.52 | 0.67296744 | 1.27E-08 | 1 |
| 16 | A549 | 0.1 | 100 | 0 | 5.52 | 0.67296744 | 1.27E-08 | 1 |
| 16 | A549 | 0.1 | 107.193177 | 72 | 5.52 | 0.67296744 | 1.27E-08 | 1 |
| 17 | A549 | 0.3 | 100 | 0 | 5.52 | 0.67296744 | 1.27E-08 | 1 |
| 17 | A549 | 0.3 | 97.7586398 | 72 | 5.52 | 0.67296744 | 1.27E-08 | 1 |
| 18 | A549 | 1 | 100 | 0 | 5.52 | 0.67296744 | 1.27E-08 | 1 |
| 18 | A549 | 1 | 76.7046965 | 72 | 5.52 | 0.67296744 | 1.27E-08 | 1 |
| 19 | A549 | 3 | 100 | 0 | 5.52 | 0.67296744 | 1.27E-08 | 1 |
| 19 | A549 | 3 | 67.8101462 | 72 | 5.52 | 0.67296744 | 1.27E-08 | 1 |
| 20 | A549 | 10 | 100 | 0 | 5.52 | 0.67296744 | 1.27E-08 | 1 |
| 20 | A549 | 10 | 32.7032565 | 72 | 5.52 | 0.67296744 | 1.27E-08 | 1 |
| 21 | H460 | 0.0003 | 100 | 0 | 7.38 | 0.65458573 | 2.69E-08 | 1 |
| 21 | H460 | 0.0003 | 99.4605671 | 72 | 7.38 | 0.65458573 | 2.69E-08 | 1 |
| 22 | H460 | 0.001 | 100 | 0 | 7.38 | 0.65458573 | 2.69E-08 | 1 |
| 22 | H460 | 0.001 | 97.8766061 | 72 | 7.38 | 0.65458573 | 2.69E-08 | 1 |
| 23 | H460 | 0.003 | 100 | 0 | 7.38 | 0.65458573 | 2.69E-08 | 1 |
| 23 | H460 | 0.003 | 100.062583 | 72 | 7.38 | 0.65458573 | 2.69E-08 | 1 |
| 24 | H460 | 0.01 | 100 | 0 | 7.38 | 0.65458573 | 2.69E-08 | 1 |
| 24 | H460 | 0.01 | 101.169141 | 72 | 7.38 | 0.65458573 | 2.69E-08 | 1 |
| 25 | H460 | 0.03 | 100 | 0 | 7.38 | 0.65458573 | 2.69E-08 | 1 |
| 25 | H460 | 0.03 | 100.923239 | 72 | 7.38 | 0.65458573 | 2.69E-08 | 1 |
| 26 | H460 | 0.1 | 100 | 0 | 7.38 | 0.65458573 | 2.69E-08 | 1 |
| 26 | H460 | 0.1 | 99.5979176 | 72 | 7.38 | 0.65458573 | 2.69E-08 | 1 |
| 27 | H460 | 0.3 | 100 | 0 | 7.38 | 0.65458573 | 2.69E-08 | 1 |
| 27 | H460 | 0.3 | 95.5710013 | 72 | 7.38 | 0.65458573 | 2.69E-08 | 1 |
| 28 | H460 | 1 | 100 | 0 | 7.38 | 0.65458573 | 2.69E-08 | 1 |
| 28 | H460 | 1 | 86.6775587 | 72 | 7.38 | 0.65458573 | 2.69E-08 | 1 |
| 29 | H460 | 3 | 100 | 0 | 7.38 | 0.65458573 | 2.69E-08 | 1 |
| 29 | H460 | 3 | 65.8822552 | 72 | 7.38 | 0.65458573 | 2.69E-08 | 1 |
| 30 | H460 | 10 | 100 | 0 | 7.38 | 0.65458573 | 2.69E-08 | 1 |
| 30 | H460 | 10 | 34.5414267 | 72 | 7.38 | 0.65458573 | 2.69E-08 | 1 |
| 31 | HCC827 | 0.0003 | 100 | 0 | 1.249 | 0.77724589 | 2.61E-08 | 1 |
| 31 | HCC827 | 0.0003 | 97.8167158 | 72 | 1.249 | 0.77724589 | 2.61E-08 | 1 |
| 32 | HCC827 | 0.001 | 100 | 0 | 1.249 | 0.77724589 | 2.61E-08 | 1 |
| 32 | HCC827 | 0.001 | 101.166772 | 72 | 1.249 | 0.77724589 | 2.61E-08 | 1 |
| 33 | HCC827 | 0.003 | 100 | 0 | 1.249 | 0.77724589 | 2.61E-08 | 1 |
| 33 | HCC827 | 0.003 | 100.472949 | 72 | 1.249 | 0.77724589 | 2.61E-08 | 1 |
| 34 | HCC827 | 0.01 | 100 | 0 | 1.249 | 0.77724589 | 2.61E-08 | 1 |
| 34 | HCC827 | 0.01 | 100.673296 | 72 | 1.249 | 0.77724589 | 2.61E-08 | 1 |
| 35 | HCC827 | 0.03 | 100 | 0 | 1.249 | 0.77724589 | 2.61E-08 | 1 |
| 35 | HCC827 | 0.03 | 99.1173259 | 72 | 1.249 | 0.77724589 | 2.61E-08 | 1 |
| 36 | HCC827 | 0.1 | 100 | 0 | 1.249 | 0.77724589 | 2.61E-08 | 1 |
| 36 | HCC827 | 0.1 | 94.0930626 | 72 | 1.249 | 0.77724589 | 2.61E-08 | 1 |
| 37 | HCC827 | 0.3 | 100 | 0 | 1.249 | 0.77724589 | 2.61E-08 | 1 |
| 37 | HCC827 | 0.3 | 82.4483328 | 72 | 1.249 | 0.77724589 | 2.61E-08 | 1 |
| 38 | HCC827 | 1 | 100 | 0 | 1.249 | 0.77724589 | 2.61E-08 | 1 |
| 38 | HCC827 | 1 | 52.8397474 | 72 | 1.249 | 0.77724589 | 2.61E-08 | 1 |
| 39 | HCC827 | 3 | 100 | 0 | 1.249 | 0.77724589 | 2.61E-08 | 1 |
| 39 | HCC827 | 3 | 37.4130669 | 72 | 1.249 | 0.77724589 | 2.61E-08 | 1 |
| 40 | HCC827 | 10 | 100 | 0 | 1.249 | 0.77724589 | 2.61E-08 | 1 |
| 40 | HCC827 | 10 | 22.2754108 | 72 | 1.249 | 0.77724589 | 2.61E-08 | 1 |
| 41 | PC9 | 0.0003 | 100 | 0 | 0.8 | 0.78880687 | 5.69E-09 | 1 |
| 41 | PC9 | 0.0003 | 90 | 72 | 0.8 | 0.78880687 | 5.69E-09 | 1 |
| 42 | PC9 | 0.001 | 100 | 0 | 0.8 | 0.78880687 | 5.69E-09 | 1 |
| 42 | PC9 | 0.001 | 102.913235 | 72 | 0.8 | 0.78880687 | 5.69E-09 | 1 |
| 43 | PC9 | 0.003 | 100 | 0 | 0.8 | 0.78880687 | 5.69E-09 | 1 |
| 43 | PC9 | 0.003 | 101.937778 | 72 | 0.8 | 0.78880687 | 5.69E-09 | 1 |
| 44 | PC9 | 0.01 | 100 | 0 | 0.8 | 0.78880687 | 5.69E-09 | 1 |
| 44 | PC9 | 0.01 | 101.81954 | 72 | 0.8 | 0.78880687 | 5.69E-09 | 1 |
| 45 | PC9 | 0.03 | 100 | 0 | 0.8 | 0.78880687 | 5.69E-09 | 1 |
| 45 | PC9 | 0.03 | 100.853936 | 72 | 0.8 | 0.78880687 | 5.69E-09 | 1 |
| 46 | PC9 | 0.1 | 100 | 0 | 0.8 | 0.78880687 | 5.69E-09 | 1 |
| 46 | PC9 | 0.1 | 96.9980869 | 72 | 0.8 | 0.78880687 | 5.69E-09 | 1 |
| 47 | PC9 | 0.3 | 100 | 0 | 0.8 | 0.78880687 | 5.69E-09 | 1 |
| 47 | PC9 | 0.3 | 74.9238437 | 72 | 0.8 | 0.78880687 | 5.69E-09 | 1 |
| 48 | PC9 | 1 | 100 | 0 | 0.8 | 0.78880687 | 5.69E-09 | 1 |
| 48 | PC9 | 1 | 43.0178423 | 72 | 0.8 | 0.78880687 | 5.69E-09 | 1 |
| 49 | PC9 | 3 | 100 | 0 | 0.8 | 0.78880687 | 5.69E-09 | 1 |
| 49 | PC9 | 3 | 30.1899186 | 72 | 0.8 | 0.78880687 | 5.69E-09 | 1 |
| 50 | PC9 | 10 | 100 | 0 | 0.8 | 0.78880687 | 5.69E-09 | 1 |
| 50 | PC9 | 10 | 21.1193129 | 72 | 0.8 | 0.78880687 | 5.69E-09 | 1 |
| 51 | H228 | 0.001 | 100 | 0 | 0.278 | 0.57479452 | 3.91E-09 | 2 |
| 51 | H228 | 0.001 | 96.4383562 | 72 | 0.278 | 0.57479452 | 3.91E-09 | 2 |
| 52 | H228 | 0.003 | 100 | 0 | 0.278 | 0.57479452 | 3.91E-09 | 2 |
| 52 | H228 | 0.003 | 96.8767123 | 72 | 0.278 | 0.57479452 | 3.91E-09 | 2 |
| 53 | H228 | 0.01 | 100 | 0 | 0.278 | 0.57479452 | 3.91E-09 | 2 |
| 53 | H228 | 0.01 | 85.0410959 | 72 | 0.278 | 0.57479452 | 3.91E-09 | 2 |
| 54 | H228 | 0.03 | 100 | 0 | 0.278 | 0.57479452 | 3.91E-09 | 2 |
| 54 | H228 | 0.03 | 76.2739726 | 72 | 0.278 | 0.57479452 | 3.91E-09 | 2 |
| 55 | H228 | 0.1 | 100 | 0 | 0.278 | 0.57479452 | 3.91E-09 | 2 |
| 55 | H228 | 0.1 | 62.2465753 | 72 | 0.278 | 0.57479452 | 3.91E-09 | 2 |
| 56 | H228 | 0.3 | 100 | 0 | 0.278 | 0.57479452 | 3.91E-09 | 2 |
| 56 | H228 | 0.3 | 49.5342466 | 72 | 0.278 | 0.57479452 | 3.91E-09 | 2 |
| 57 | H228 | 1 | 100 | 0 | 0.278 | 0.57479452 | 3.91E-09 | 2 |
| 57 | H228 | 1 | 42.520548 | 72 | 0.278 | 0.57479452 | 3.91E-09 | 2 |
| 58 | H3122 | 0.001 | 100 | 0 | 0.023 | 0.8342711 | 5.14E-09 | 2 |
| 58 | H3122 | 0.001 | 91.6112532 | 72 | 0.023 | 0.8342711 | 5.14E-09 | 2 |
| 59 | H3122 | 0.003 | 100 | 0 | 0.023 | 0.8342711 | 5.14E-09 | 2 |
| 59 | H3122 | 0.003 | 80.5626599 | 72 | 0.023 | 0.8342711 | 5.14E-09 | 2 |
| 60 | H3122 | 0.01 | 100 | 0 | 0.023 | 0.8342711 | 5.14E-09 | 2 |
| 60 | H3122 | 0.01 | 66.7519182 | 72 | 0.023 | 0.8342711 | 5.14E-09 | 2 |
| 61 | H3122 | 0.03 | 100 | 0 | 0.023 | 0.8342711 | 5.14E-09 | 2 |
| 61 | H3122 | 0.03 | 45.5754476 | 72 | 0.023 | 0.8342711 | 5.14E-09 | 2 |
| 62 | H3122 | 0.1 | 100 | 0 | 0.023 | 0.8342711 | 5.14E-09 | 2 |
| 62 | H3122 | 0.1 | 28.5421995 | 72 | 0.023 | 0.8342711 | 5.14E-09 | 2 |
| 63 | H3122 | 0.3 | 100 | 0 | 0.023 | 0.8342711 | 5.14E-09 | 2 |
| 63 | H3122 | 0.3 | 19.3350384 | 72 | 0.023 | 0.8342711 | 5.14E-09 | 2 |
| 64 | H3122 | 1 | 100 | 0 | 0.023 | 0.8342711 | 5.14E-09 | 2 |
| 64 | H3122 | 1 | 16.57289 | 72 | 0.023 | 0.8342711 | 5.14E-09 | 2 |
| 65 | A925LPE3 | 0.01 | 100 | 0 | 0.446 | 0.9843 | 8.98E-10 | 3 |
| 65 | A925LPE3 | 0.01 | 90.7534247 | 72 | 0.446 | 0.9843 | 8.98E-10 | 3 |
| 66 | A925LPE3 | 0.03 | 100 | 0 | 0.446 | 0.9843 | 8.98E-10 | 3 |
| 66 | A925LPE3 | 0.03 | 92.1232877 | 72 | 0.446 | 0.9843 | 8.98E-10 | 3 |
| 67 | A925LPE3 | 0.1 | 100 | 0 | 0.446 | 0.9843 | 8.98E-10 | 3 |
| 67 | A925LPE3 | 0.1 | 75.6849315 | 72 | 0.446 | 0.9843 | 8.98E-10 | 3 |
| 68 | A925LPE3 | 0.3 | 100 | 0 | 0.446 | 0.9843 | 8.98E-10 | 3 |
| 68 | A925LPE3 | 0.3 | 56.1643836 | 72 | 0.446 | 0.9843 | 8.98E-10 | 3 |
| 69 | A925LPE3 | 1 | 100 | 0 | 0.446 | 0.9843 | 8.98E-10 | 3 |
| 69 | A925LPE3 | 1 | 33.5616438 | 72 | 0.446 | 0.9843 | 8.98E-10 | 3 |
| 70 | A925LPE3 | 3 | 100 | 0 | 0.446 | 0.9843 | 8.98E-10 | 3 |
| 70 | A925LPE3 | 3 | 9.93150685 | 72 | 0.446 | 0.9843 | 8.98E-10 | 3 |
| 71 | A925LPE3 | 10 | 100 | 0 | 0.446 | 0.9843 | 8.98E-10 | 3 |
| 71 | A925LPE3 | 10 | 1.57 | 72 | 0.446 | 0.9843 | 8.98E-10 | 3 |
| 72 | H228 | 0.01 | 100 | 0 | 0.056 | 0.67944991 | 9.79E-09 | 4 |
| 72 | H228 | 0.01 | 75.1733005 | 72 | 0.056 | 0.67944991 | 9.79E-09 | 4 |
| 73 | H228 | 0.03 | 100 | 0 | 0.056 | 0.67944991 | 9.79E-09 | 4 |
| 73 | H228 | 0.03 | 54.0557916 | 72 | 0.056 | 0.67944991 | 9.79E-09 | 4 |
| 74 | H228 | 0.1 | 100 | 0 | 0.056 | 0.67944991 | 9.79E-09 | 4 |
| 74 | H228 | 0.1 | 44.0993962 | 72 | 0.056 | 0.67944991 | 9.79E-09 | 4 |
| 75 | H228 | 0.3 | 100 | 0 | 0.056 | 0.67944991 | 9.79E-09 | 4 |
| 75 | H228 | 0.3 | 39.3392218 | 72 | 0.056 | 0.67944991 | 9.79E-09 | 4 |
| 76 | H228 | 1 | 100 | 0 | 0.056 | 0.67944991 | 9.79E-09 | 4 |
| 76 | H228 | 1 | 34.5832402 | 72 | 0.056 | 0.67944991 | 9.79E-09 | 4 |
| 77 | H228 | 3 | 100 | 0 | 0.056 | 0.67944991 | 9.79E-09 | 4 |
| 77 | H228 | 3 | 32.0550089 | 72 | 0.056 | 0.67944991 | 9.79E-09 | 4 |
| 78 | LC-2 ad | 0.001 | 100 | 0 | 0.19 | 0.77240857 | 5.93E-09 | 5 |
| 78 | LC-2 ad | 0.001 | 100.10171 | 72 | 0.19 | 0.77240857 | 5.93E-09 | 5 |
| 79 | LC-2 ad | 0.003 | 100 | 0 | 0.19 | 0.77240857 | 5.93E-09 | 5 |
| 79 | LC-2 ad | 0.003 | 104.502272 | 72 | 0.19 | 0.77240857 | 5.93E-09 | 5 |
| 80 | LC-2 ad | 0.01 | 100 | 0 | 0.19 | 0.77240857 | 5.93E-09 | 5 |
| 80 | LC-2 ad | 0.01 | 94.4395153 | 72 | 0.19 | 0.77240857 | 5.93E-09 | 5 |
| 81 | LC-2 ad | 0.03 | 100 | 0 | 0.19 | 0.77240857 | 5.93E-09 | 5 |
| 81 | LC-2 ad | 0.03 | 84.3767583 | 72 | 0.19 | 0.77240857 | 5.93E-09 | 5 |
| 82 | LC-2 ad | 0.1 | 100 | 0 | 0.19 | 0.77240857 | 5.93E-09 | 5 |
| 82 | LC-2 ad | 0.1 | 62.2181346 | 72 | 0.19 | 0.77240857 | 5.93E-09 | 5 |
| 83 | LC-2 ad | 0.3 | 100 | 0 | 0.19 | 0.77240857 | 5.93E-09 | 5 |
| 83 | LC-2 ad | 0.3 | 42.3901753 | 72 | 0.19 | 0.77240857 | 5.93E-09 | 5 |
| 84 | LC-2 ad | 1 | 100 | 0 | 0.19 | 0.77240857 | 5.93E-09 | 5 |
| 84 | LC-2 ad | 1 | 21.7874919 | 72 | 0.19 | 0.77240857 | 5.93E-09 | 5 |
| 85 | LC-2 ad | 3 | 100 | 0 | 0.19 | 0.77240857 | 5.93E-09 | 5 |
| 85 | LC-2 ad | 3 | 18.7578446 | 72 | 0.19 | 0.77240857 | 5.93E-09 | 5 |
| 86 | LC-2 ad | 10 | 100 | 0 | 0.19 | 0.77240857 | 5.93E-09 | 5 |
| 86 | LC-2 ad | 10 | 22.759143 | 72 | 0.19 | 0.77240857 | 5.93E-09 | 5 |
| 87 | H3122 | 0.001 | 100 | 0 | 0.0122 | 0.92231831 | 6.43E-09 | 6 |
| 87 | H3122 | 0.001 | 94.040274 | 72 | 0.0122 | 0.92231831 | 6.43E-09 | 6 |
| 88 | H3122 | 0.003 | 100 | 0 | 0.0122 | 0.92231831 | 6.43E-09 | 6 |
| 88 | H3122 | 0.003 | 75.3680043 | 72 | 0.0122 | 0.92231831 | 6.43E-09 | 6 |
| 89 | H3122 | 0.01 | 100 | 0 | 0.0122 | 0.92231831 | 6.43E-09 | 6 |
| 89 | H3122 | 0.01 | 62.6894676 | 72 | 0.0122 | 0.92231831 | 6.43E-09 | 6 |
| 90 | H3122 | 0.03 | 100 | 0 | 0.0122 | 0.92231831 | 6.43E-09 | 6 |
| 90 | H3122 | 0.03 | 20.2025845 | 72 | 0.0122 | 0.92231831 | 6.43E-09 | 6 |
| 91 | H3122 | 0.1 | 100 | 0 | 0.0122 | 0.92231831 | 6.43E-09 | 6 |
| 91 | H3122 | 0.1 | 13.9938787 | 72 | 0.0122 | 0.92231831 | 6.43E-09 | 6 |
| 92 | H3122 | 0.3 | 100 | 0 | 0.0122 | 0.92231831 | 6.43E-09 | 6 |
| 92 | H3122 | 0.3 | 13.4424796 | 72 | 0.0122 | 0.92231831 | 6.43E-09 | 6 |
| 93 | H3122 | 1 | 100 | 0 | 0.0122 | 0.92231831 | 6.43E-09 | 6 |
| 93 | H3122 | 1 | 13.7157501 | 72 | 0.0122 | 0.92231831 | 6.43E-09 | 6 |
| 94 | H3122 | 3 | 100 | 0 | 0.0122 | 0.92231831 | 6.43E-09 | 6 |
| 94 | H3122 | 3 | 7.92241547 | 72 | 0.0122 | 0.92231831 | 6.43E-09 | 6 |
| 95 | H3122 | 10 | 100 | 0 | 0.0122 | 0.92231831 | 6.43E-09 | 6 |
| 95 | H3122 | 10 | 7.76816945 | 72 | 0.0122 | 0.92231831 | 6.43E-09 | 6 |
| 96 | A925L | 0.001 | 100 | 0 | 0.0235 | 0.72887638 | 8.98E-10 | 7 |
| 96 | A925L | 0.001 | 79.6636996 | 72 | 0.0235 | 0.72887638 | 8.98E-10 | 7 |
| 97 | A925L | 0.003 | 100 | 0 | 0.0235 | 0.72887638 | 8.98E-10 | 7 |
| 97 | A925L | 0.003 | 76.3642759 | 72 | 0.0235 | 0.72887638 | 8.98E-10 | 7 |
| 98 | A925L | 0.01 | 100 | 0 | 0.0235 | 0.72887638 | 8.98E-10 | 7 |
| 98 | A925L | 0.01 | 67.1045156 | 72 | 0.0235 | 0.72887638 | 8.98E-10 | 7 |
| 99 | A925L | 0.03 | 100 | 0 | 0.0235 | 0.72887638 | 8.98E-10 | 7 |
| 99 | A925L | 0.03 | 46.2000487 | 72 | 0.0235 | 0.72887638 | 8.98E-10 | 7 |
| 100 | A925L | 0.1 | 100 | 0 | 0.0235 | 0.72887638 | 8.98E-10 | 7 |
| 100 | A925L | 0.1 | 34.1021617 | 72 | 0.0235 | 0.72887638 | 8.98E-10 | 7 |
| 101 | A925L | 0.3 | 100 | 0 | 0.0235 | 0.72887638 | 8.98E-10 | 7 |
| 101 | A925L | 0.3 | 27.1123617 | 72 | 0.0235 | 0.72887638 | 8.98E-10 | 7 |
| 102 | A925LPE3 | 0.001 | 100 | 0 | 0.05 | 0.64931955 | 8.98E-10 | 7 |
| 102 | A925LPE3 | 0.001 | 74.8302265 | 72 | 0.05 | 0.64931955 | 8.98E-10 | 7 |
| 103 | A925LPE3 | 0.003 | 100 | 0 | 0.05 | 0.64931955 | 8.98E-10 | 7 |
| 103 | A925LPE3 | 0.003 | 73.8088796 | 72 | 0.05 | 0.64931955 | 8.98E-10 | 7 |
| 104 | A925LPE3 | 0.01 | 100 | 0 | 0.05 | 0.64931955 | 8.98E-10 | 7 |
| 104 | A925LPE3 | 0.01 | 70.2321366 | 72 | 0.05 | 0.64931955 | 8.98E-10 | 7 |
| 105 | A925LPE3 | 0.03 | 100 | 0 | 0.05 | 0.64931955 | 8.98E-10 | 7 |
| 105 | A925LPE3 | 0.03 | 54.7238982 | 72 | 0.05 | 0.64931955 | 8.98E-10 | 7 |
| 106 | A925LPE3 | 0.1 | 100 | 0 | 0.05 | 0.64931955 | 8.98E-10 | 7 |
| 106 | A925LPE3 | 0.1 | 46.3150348 | 72 | 0.05 | 0.64931955 | 8.98E-10 | 7 |
| 107 | A925LPE3 | 0.3 | 100 | 0 | 0.05 | 0.64931955 | 8.98E-10 | 7 |
| 107 | A925LPE3 | 0.3 | 35.0680447 | 72 | 0.05 | 0.64931955 | 8.98E-10 | 7 |
| 108 | H3122 | 0.001 | 100 | 0 | 0.0033 | 0.92488028 | 5.14E-09 | 7 |
| 108 | H3122 | 0.001 | 76.5347258 | 72 | 0.0033 | 0.92488028 | 5.14E-09 | 7 |
| 109 | H3122 | 0.003 | 100 | 0 | 0.0033 | 0.92488028 | 5.14E-09 | 7 |
| 109 | H3122 | 0.003 | 51.0862801 | 72 | 0.0033 | 0.92488028 | 5.14E-09 | 7 |
| 110 | H3122 | 0.01 | 100 | 0 | 0.0033 | 0.92488028 | 5.14E-09 | 7 |
| 110 | H3122 | 0.01 | 21.9406943 | 72 | 0.0033 | 0.92488028 | 5.14E-09 | 7 |
| 111 | H3122 | 0.03 | 100 | 0 | 0.0033 | 0.92488028 | 5.14E-09 | 7 |
| 111 | H3122 | 0.03 | 9.55331295 | 72 | 0.0033 | 0.92488028 | 5.14E-09 | 7 |
| 112 | H3122 | 0.1 | 100 | 0 | 0.0033 | 0.92488028 | 5.14E-09 | 7 |
| 112 | H3122 | 0.1 | 8.2478829 | 72 | 0.0033 | 0.92488028 | 5.14E-09 | 7 |
| 113 | H3122 | 0.3 | 100 | 0 | 0.0033 | 0.92488028 | 5.14E-09 | 7 |
| 113 | H3122 | 0.3 | 7.51197208 | 72 | 0.0033 | 0.92488028 | 5.14E-09 | 7 |
| 114 | A925 | 0.01 | 100 | 0 | 0.33 | 0.87297439 | 8.98E-10 | 8 |
| 114 | A925 | 0.01 | 82.6557085 | 72 | 0.33 | 0.87297439 | 8.98E-10 | 8 |
| 115 | A925 | 0.03 | 100 | 0 | 0.33 | 0.87297439 | 8.98E-10 | 8 |
| 115 | A925 | 0.03 | 73.7594626 | 72 | 0.33 | 0.87297439 | 8.98E-10 | 8 |
| 116 | A925 | 0.1 | 100 | 0 | 0.33 | 0.87297439 | 8.98E-10 | 8 |
| 116 | A925 | 0.1 | 66.5427581 | 72 | 0.33 | 0.87297439 | 8.98E-10 | 8 |
| 117 | A925 | 0.3 | 100 | 0 | 0.33 | 0.87297439 | 8.98E-10 | 8 |
| 117 | A925 | 0.3 | 51.2332772 | 72 | 0.33 | 0.87297439 | 8.98E-10 | 8 |
| 118 | A925 | 1 | 100 | 0 | 0.33 | 0.87297439 | 8.98E-10 | 8 |
| 118 | A925 | 1 | 37.9334379 | 72 | 0.33 | 0.87297439 | 8.98E-10 | 8 |
| 119 | A925 | 3 | 100 | 0 | 0.33 | 0.87297439 | 8.98E-10 | 8 |
| 119 | A925 | 3 | 12.8303421 | 72 | 0.33 | 0.87297439 | 8.98E-10 | 8 |
| 120 | A925 | 10 | 100 | 0 | 0.33 | 0.87297439 | 8.98E-10 | 8 |
| 120 | A925 | 10 | 12.7025614 | 72 | 0.33 | 0.87297439 | 8.98E-10 | 8 |

Table S1: Cell viability data for NSCLC cell lines that were treated with alectinib. Note that the expression “E-x” refers to x10^-x^, Monolix uses this format to express powers. The cell volume is calculated based on the cell micrographs and the number of cells in each well. Conc= Concentration.

Note: The data presented is based on the data extracted from these plots using WebPlotDigitizer (https://automeris.io/WebPlotDigitizer). The reference number refers to the original source for the cell viability curves:

1. Katayama R, et al. Two Novel ALK Mutations Mediate Acquired Resistance to the Next-Generation ALK Inhibitor Alectinib. *Clinical Cancer Research*. 20(22):5686-5696 (2014).
2. Tanimoto A, et al. Receptor ligand-triggered resistance to alectinib and its circumvention by Hsp90 inhibition in EML4-ALK lung cancer cells. *Oncotarget*. 5(13):4920-4928 (2014).
3. Arai S, et al. Osimertinib Overcomes Alectinib Resistance Caused by Amphiregulin in a Leptomeningeal Carcinomatosis Model of ALK-Rearranged Lung Cancer. *Journal of Thoracic Oncology*. 15(5):752-765 (2020).
4. Tsuji T, et al. Alectinib Resistance in ALK-Rearranged Lung Cancer by Dual Salvage Signaling in a Clinically Paired Resistance Model. *Molecular Cancer Research*. 17(1):212-224 (2018).
5. Arai S, et al. In vitro and in vivo anti-tumor activity of alectinib in tumor cells with NCOA4-RET. *Oncotarget*. 8(43):73766-73773 (2017).
6. Katayama R, et al. P-glycoprotein Mediates Ceritinib Resistance in Anaplastic Lymphoma Kinase-rearranged Non-small Cell Lung Cancer. *EBioMedicine*. 3:54-66 (2016).
7. Nanjo S, et al. In vivo imaging models of bone and brain metastases and pleural carcinomatosis with a novel human EML 4‐ ALK lung cancer cell line. *Cancer Science.* 106(3):244-252 (2015).
8. Fukuda K, et al. Epithelial-to-Mesenchymal Transition Is a Mechanism of ALK Inhibitor Resistance in Lung Cancer Independent of ALK Mutation Status. *Cancer Research*. 79(7):1658-1670 (2019).

**Table S2: Tumour cell viability data for crizotinib**

| ID | Cell Line | Conc (μM) | Cell Viability (%) | Time (hr) | *IC_50_* (μM) | *I_MAX_* | *V_C(0)_* (L) | Ref |
| --- | --- | --- | --- | --- | --- | --- | --- | --- |
| 1 | H3122 | 0.001 | 100 | 0 | 0.845 | 0.94307639 | 6.43E-09 | 1 |
| 1 | H3122 | 0.001 | 97.0755545 | 72 | 0.845 | 0.94307639 | 6.43E-09 | 1 |
| 2 | H3122 | 0.003 | 100 | 0 | 0.845 | 0.94307639 | 6.43E-09 | 1 |
| 2 | H3122 | 0.003 | 94.891765 | 72 | 0.845 | 0.94307639 | 6.43E-09 | 1 |
| 3 | H3122 | 0.01 | 100 | 0 | 0.845 | 0.94307639 | 6.43E-09 | 1 |
| 3 | H3122 | 0.01 | 85.8282747 | 72 | 0.845 | 0.94307639 | 6.43E-09 | 1 |
| 4 | H3122 | 0.03 | 100 | 0 | 0.845 | 0.94307639 | 6.43E-09 | 1 |
| 4 | H3122 | 0.03 | 70.9197114 | 72 | 0.845 | 0.94307639 | 6.43E-09 | 1 |
| 5 | H3122 | 0.1 | 100 | 0 | 0.845 | 0.94307639 | 6.43E-09 | 1 |
| 5 | H3122 | 0.1 | 38.1552323 | 72 | 0.845 | 0.94307639 | 6.43E-09 | 1 |
| 6 | H3122 | 0.3 | 100 | 0 | 0.845 | 0.94307639 | 6.43E-09 | 1 |
| 6 | H3122 | 0.3 | 20.5016608 | 72 | 0.845 | 0.94307639 | 6.43E-09 | 1 |
| 7 | H3122 | 1 | 100 | 0 | 0.845 | 0.94307639 | 6.43E-09 | 1 |
| 7 | H3122 | 1 | 13.1561868 | 72 | 0.845 | 0.94307639 | 6.43E-09 | 1 |
| 8 | H3122 | 3 | 100 | 0 | 0.845 | 0.94307639 | 6.43E-09 | 1 |
| 8 | H3122 | 3 | 7.18894361 | 72 | 0.845 | 0.94307639 | 6.43E-09 | 1 |
| 9 | H3122 | 10 | 100 | 0 | 0.845 | 0.94307639 | 6.43E-09 | 1 |
| 9 | H3122 | 10 | 5.69236055 | 72 | 0.845 | 0.94307639 | 6.43E-09 | 1 |
| 10 | A549 | 0.001 | 100 | 0 | 1.81 | 0.91910052 | 1.27E-08 | 1 |
| 10 | A549 | 0.001 | 100.167984 | 72 | 1.81 | 0.91910052 | 1.27E-08 | 1 |
| 11 | A549 | 0.003 | 100 | 0 | 1.81 | 0.91910052 | 1.27E-08 | 1 |
| 11 | A549 | 0.003 | 99.6945749 | 72 | 1.81 | 0.91910052 | 1.27E-08 | 1 |
| 12 | A549 | 0.01 | 100 | 0 | 1.81 | 0.91910052 | 1.27E-08 | 1 |
| 12 | A549 | 0.01 | 100.603215 | 72 | 1.81 | 0.91910052 | 1.27E-08 | 1 |
| 13 | A549 | 0.03 | 100 | 0 | 1.81 | 0.91910052 | 1.27E-08 | 1 |
| 13 | A549 | 0.03 | 97.0373764 | 72 | 1.81 | 0.91910052 | 1.27E-08 | 1 |
| 14 | A549 | 0.1 | 100 | 0 | 1.81 | 0.91910052 | 1.27E-08 | 1 |
| 14 | A549 | 0.1 | 100.343603 | 72 | 1.81 | 0.91910052 | 1.27E-08 | 1 |
| 15 | A549 | 0.3 | 100 | 0 | 1.81 | 0.91910052 | 1.27E-08 | 1 |
| 15 | A549 | 0.3 | 96.4456152 | 72 | 1.81 | 0.91910052 | 1.27E-08 | 1 |
| 16 | A549 | 1 | 100 | 0 | 1.81 | 0.91910052 | 1.27E-08 | 1 |
| 16 | A549 | 1 | 61.6042454 | 72 | 1.81 | 0.91910052 | 1.27E-08 | 1 |
| 17 | A549 | 3 | 100 | 0 | 1.81 | 0.91910052 | 1.27E-08 | 1 |
| 17 | A549 | 3 | 33.6425763 | 72 | 1.81 | 0.91910052 | 1.27E-08 | 1 |
| 18 | A549 | 10 | 100 | 0 | 1.81 | 0.91910052 | 1.27E-08 | 1 |
| 18 | A549 | 10 | 8.0899477 | 72 | 1.81 | 0.91910052 | 1.27E-08 | 1 |
| 19 | H460 | 0.001 | 100 | 0 | 2.9743 | 0.96388348 | 2.69E-08 | 1 |
| 19 | H460 | 0.001 | 102.91681 | 72 | 2.9743 | 0.96388348 | 2.69E-08 | 1 |
| 20 | H460 | 0.003 | 100 | 0 | 2.9743 | 0.96388348 | 2.69E-08 | 1 |
| 20 | H460 | 0.003 | 101.416409 | 72 | 2.9743 | 0.96388348 | 2.69E-08 | 1 |
| 21 | H460 | 0.01 | 100 | 0 | 2.9743 | 0.96388348 | 2.69E-08 | 1 |
| 21 | H460 | 0.01 | 101.981445 | 72 | 2.9743 | 0.96388348 | 2.69E-08 | 1 |
| 22 | H460 | 0.03 | 100 | 0 | 2.9743 | 0.96388348 | 2.69E-08 | 1 |
| 22 | H460 | 0.03 | 101.511854 | 72 | 2.9743 | 0.96388348 | 2.69E-08 | 1 |
| 23 | H460 | 0.1 | 100 | 0 | 2.9743 | 0.96388348 | 2.69E-08 | 1 |
| 23 | H460 | 0.1 | 98.625587 | 72 | 2.9743 | 0.96388348 | 2.69E-08 | 1 |
| 24 | H460 | 0.3 | 100 | 0 | 2.9743 | 0.96388348 | 2.69E-08 | 1 |
| 24 | H460 | 0.3 | 92.3185584 | 72 | 2.9743 | 0.96388348 | 2.69E-08 | 1 |
| 25 | H460 | 1 | 100 | 0 | 2.9743 | 0.96388348 | 2.69E-08 | 1 |
| 25 | H460 | 1 | 76.0432177 | 72 | 2.9743 | 0.96388348 | 2.69E-08 | 1 |
| 26 | H460 | 3 | 100 | 0 | 2.9743 | 0.96388348 | 2.69E-08 | 1 |
| 26 | H460 | 3 | 49.4521437 | 72 | 2.9743 | 0.96388348 | 2.69E-08 | 1 |
| 27 | H460 | 10 | 100 | 0 | 2.9743 | 0.96388348 | 2.69E-08 | 1 |
| 27 | H460 | 10 | 3.61165197 | 72 | 2.9743 | 0.96388348 | 2.69E-08 | 1 |
| 28 | HCC827 | 0.001 | 100 | 0 | 6.06973094 | 0.78853129 | 2.61E-08 | 1 |
| 28 | HCC827 | 0.001 | 107.036231 | 72 | 6.06973094 | 0.78853129 | 2.61E-08 | 1 |
| 29 | HCC827 | 0.003 | 100 | 0 | 6.06973094 | 0.78853129 | 2.61E-08 | 1 |
| 30 | HCC827 | 0.01 | 100 | 0 | 6.06973094 | 0.78853129 | 2.61E-08 | 1 |
| 30 | HCC827 | 0.01 | 102.321231 | 72 | 6.06973094 | 0.78853129 | 2.61E-08 | 1 |
| 31 | HCC827 | 0.03 | 100 | 0 | 6.06973094 | 0.78853129 | 2.61E-08 | 1 |
| 31 | HCC827 | 0.03 | 101.156798 | 72 | 6.06973094 | 0.78853129 | 2.61E-08 | 1 |
| 32 | HCC827 | 0.1 | 100 | 0 | 6.06973094 | 0.78853129 | 2.61E-08 | 1 |
| 32 | HCC827 | 0.1 | 99.6678502 | 72 | 6.06973094 | 0.78853129 | 2.61E-08 | 1 |
| 33 | HCC827 | 0.3 | 100 | 0 | 6.06973094 | 0.78853129 | 2.61E-08 | 1 |
| 33 | HCC827 | 0.3 | 96.0829229 | 72 | 6.06973094 | 0.78853129 | 2.61E-08 | 1 |
| 34 | HCC827 | 1 | 100 | 0 | 6.06973094 | 0.78853129 | 2.61E-08 | 1 |
| 34 | HCC827 | 1 | 91.8413317 | 72 | 6.06973094 | 0.78853129 | 2.61E-08 | 1 |
| 35 | HCC827 | 3 | 100 | 0 | 6.06973094 | 0.78853129 | 2.61E-08 | 1 |
| 35 | HCC827 | 3 | 71.4427519 | 72 | 6.06973094 | 0.78853129 | 2.61E-08 | 1 |
| 36 | HCC827 | 10 | 100 | 0 | 6.06973094 | 0.78853129 | 2.61E-08 | 1 |
| 36 | HCC827 | 10 | 21.1468713 | 72 | 6.06973094 | 0.78853129 | 2.61E-08 | 1 |
| 37 | PC9 | 0.001 | 100 | 0 | 2.792 | 0.987783 | 5.69E-09 | 1 |
| 37 | PC9 | 0.001 | 102.229603 | 72 | 2.792 | 0.987783 | 5.69E-09 | 1 |
| 38 | PC9 | 0.003 | 100 | 0 | 2.792 | 0.987783 | 5.69E-09 | 1 |
| 38 | PC9 | 0.003 | 101.416409 | 72 | 2.792 | 0.987783 | 5.69E-09 | 1 |
| 39 | PC9 | 0.01 | 100 | 0 | 2.792 | 0.987783 | 5.69E-09 | 1 |
| 39 | PC9 | 0.01 | 99.221166 | 72 | 2.792 | 0.987783 | 5.69E-09 | 1 |
| 40 | PC9 | 0.03 | 100 | 0 | 2.792 | 0.987783 | 5.69E-09 | 1 |
| 40 | PC9 | 0.03 | 100.137441 | 72 | 2.792 | 0.987783 | 5.69E-09 | 1 |
| 41 | PC9 | 0.1 | 100 | 0 | 2.792 | 0.987783 | 5.69E-09 | 1 |
| 41 | PC9 | 0.1 | 99.3127935 | 72 | 2.792 | 0.987783 | 5.69E-09 | 1 |
| 42 | PC9 | 0.3 | 100 | 0 | 2.792 | 0.987783 | 5.69E-09 | 1 |
| 42 | PC9 | 0.3 | 93.700607 | 72 | 2.792 | 0.987783 | 5.69E-09 | 1 |
| 43 | PC9 | 1 | 100 | 0 | 2.792 | 0.987783 | 5.69E-09 | 1 |
| 43 | PC9 | 1 | 66.4223266 | 72 | 2.792 | 0.987783 | 5.69E-09 | 1 |
| 44 | PC9 | 3 | 100 | 0 | 2.792 | 0.987783 | 5.69E-09 | 1 |
| 44 | PC9 | 3 | 33.6463941 | 72 | 2.792 | 0.987783 | 5.69E-09 | 1 |
| 45 | PC9 | 10 | 100 | 0 | 2.792 | 0.987783 | 5.69E-09 | 1 |
| 45 | PC9 | 10 | 1.22170045 | 72 | 2.792 | 0.987783 | 5.69E-09 | 1 |
| 46 | A925 | 0.01 | 100 | 0 | 0.57 | 0.9825296 | 8.98E-10 | 2 |
| 46 | A925 | 0.01 | 74.377275 | 72 | 0.57 | 0.9825296 | 8.98E-10 | 2 |
| 47 | A925 | 0.03 | 100 | 0 | 0.57 | 0.9825296 | 8.98E-10 | 2 |
| 47 | A925 | 0.03 | 75.4518275 | 72 | 0.57 | 0.9825296 | 8.98E-10 | 2 |
| 48 | A925 | 0.1 | 100 | 0 | 0.57 | 0.9825296 | 8.98E-10 | 2 |
| 48 | A925 | 0.1 | 72.4621131 | 72 | 0.57 | 0.9825296 | 8.98E-10 | 2 |
| 49 | A925 | 0.3 | 100 | 0 | 0.57 | 0.9825296 | 8.98E-10 | 2 |
| 49 | A925 | 0.3 | 65.7649942 | 72 | 0.57 | 0.9825296 | 8.98E-10 | 2 |
| 50 | A925 | 1 | 100 | 0 | 0.57 | 0.9825296 | 8.98E-10 | 2 |
| 50 | A925 | 1 | 34.0645049 | 72 | 0.57 | 0.9825296 | 8.98E-10 | 2 |
| 51 | A925 | 3 | 100 | 0 | 0.57 | 0.9825296 | 8.98E-10 | 2 |
| 51 | A925 | 3 | 17.9105306 | 72 | 0.57 | 0.9825296 | 8.98E-10 | 2 |
| 52 | A925 | 10 | 100 | 0 | 0.57 | 0.9825296 | 8.98E-10 | 2 |
| 52 | A925 | 10 | 1.74704002 | 72 | 0.57 | 0.9825296 | 8.98E-10 | 2 |
| 53 | H3122 | 0.001 | 100 | 0 | 0.06971 | 0.95482816 | 6.43E-09 | 3 |
| 53 | H3122 | 0.001 | 95.2680826 | 72 | 0.06971 | 0.95482816 | 6.43E-09 | 3 |
| 54 | H3122 | 0.003 | 100 | 0 | 0.06971 | 0.95482816 | 6.43E-09 | 3 |
| 54 | H3122 | 0.003 | 84.2087696 | 72 | 0.06971 | 0.95482816 | 6.43E-09 | 3 |
| 55 | H3122 | 0.01 | 100 | 0 | 0.06971 | 0.95482816 | 6.43E-09 | 3 |
| 55 | H3122 | 0.01 | 91.6943891 | 72 | 0.06971 | 0.95482816 | 6.43E-09 | 3 |
| 56 | H3122 | 0.03 | 100 | 0 | 0.06971 | 0.95482816 | 6.43E-09 | 3 |
| 56 | H3122 | 0.03 | 68.2740128 | 72 | 0.06971 | 0.95482816 | 6.43E-09 | 3 |
| 57 | H3122 | 0.1 | 100 | 0 | 0.06971 | 0.95482816 | 6.43E-09 | 3 |
| 57 | H3122 | 0.1 | 42.5472024 | 72 | 0.06971 | 0.95482816 | 6.43E-09 | 3 |
| 58 | H3122 | 0.3 | 100 | 0 | 0.06971 | 0.95482816 | 6.43E-09 | 3 |
| 58 | H3122 | 0.3 | 17.2131421 | 72 | 0.06971 | 0.95482816 | 6.43E-09 | 3 |
| 59 | H3122 | 1 | 100 | 0 | 0.06971 | 0.95482816 | 6.43E-09 | 3 |
| 59 | H3122 | 1 | 8.09755338 | 72 | 0.06971 | 0.95482816 | 6.43E-09 | 3 |
| 60 | H3122 | 3 | 100 | 0 | 0.06971 | 0.95482816 | 6.43E-09 | 3 |
| 60 | H3122 | 3 | 5.1558206 | 72 | 0.06971 | 0.95482816 | 6.43E-09 | 3 |
| 61 | H3122 | 10 | 100 | 0 | 0.06971 | 0.95482816 | 6.43E-09 | 3 |
| 61 | H3122 | 10 | 4.51718421 | 72 | 0.06971 | 0.95482816 | 6.43E-09 | 3 |
| 62 | A925L | 0.003 | 100 | 0 | 0.2 | 0.78371422 | 8.98E-10 | 4 |
| 62 | A925L | 0.003 | 99.9951736 | 72 | 0.2 | 0.78371422 | 8.98E-10 | 4 |
| 63 | A925L | 0.01 | 100 | 0 | 0.2 | 0.78371422 | 8.98E-10 | 4 |
| 63 | A925L | 0.01 | 98.1611525 | 72 | 0.2 | 0.78371422 | 8.98E-10 | 4 |
| 64 | A925L | 0.03 | 100 | 0 | 0.2 | 0.78371422 | 8.98E-10 | 4 |
| 64 | A925L | 0.03 | 90.8435693 | 72 | 0.2 | 0.78371422 | 8.98E-10 | 4 |
| 65 | A925L | 0.1 | 100 | 0 | 0.2 | 0.78371422 | 8.98E-10 | 4 |
| 65 | A925L | 0.1 | 69.197294 | 72 | 0.2 | 0.78371422 | 8.98E-10 | 4 |
| 66 | A925L | 0.3 | 100 | 0 | 0.2 | 0.78371422 | 8.98E-10 | 4 |
| 66 | A925L | 0.3 | 39.0187987 | 72 | 0.2 | 0.78371422 | 8.98E-10 | 4 |
| 67 | A925L | 1 | 100 | 0 | 0.2 | 0.78371422 | 8.98E-10 | 4 |
| 67 | A925L | 1 | 21.6285786 | 72 | 0.2 | 0.78371422 | 8.98E-10 | 4 |
| 68 | A925LPE3 | 0.003 | 100 | 0 | 0.38 | 0.75 | 8.98E-10 | 4 |
| 68 | A925LPE3 | 0.003 | 84.7518843 | 72 | 0.38 | 0.75 | 8.98E-10 | 4 |
| 69 | A925LPE3 | 0.01 | 100 | 0 | 0.38 | 0.75 | 8.98E-10 | 4 |
| 69 | A925LPE3 | 0.01 | 81.9911999 | 72 | 0.38 | 0.75 | 8.98E-10 | 4 |
| 70 | A925LPE3 | 0.03 | 100 | 0 | 0.38 | 0.75 | 8.98E-10 | 4 |
| 70 | A925LPE3 | 0.03 | 81.6935737 | 72 | 0.38 | 0.75 | 8.98E-10 | 4 |
| 71 | A925LPE3 | 0.1 | 100 | 0 | 0.38 | 0.75 | 8.98E-10 | 4 |
| 71 | A925LPE3 | 0.1 | 73.4533491 | 72 | 0.38 | 0.75 | 8.98E-10 | 4 |
| 72 | A925LPE3 | 0.3 | 100 | 0 | 0.38 | 0.75 | 8.98E-10 | 4 |
| 72 | A925LPE3 | 0.3 | 53.0184931 | 72 | 0.38 | 0.75 | 8.98E-10 | 4 |
| 73 | A925LPE3 | 1 | 100 | 0 | 0.38 | 0.75 | 8.98E-10 | 4 |
| 73 | A925LPE3 | 1 | 25.2990339 | 72 | 0.38 | 0.75 | 8.98E-10 | 4 |
| 74 | H3122 | 0.003 | 100 | 0 | 0.041 | 0.97265056 | 5.14E-09 | 4 |
| 74 | H3122 | 0.003 | 94.8124553 | 72 | 0.041 | 0.97265056 | 5.14E-09 | 4 |
| 75 | H3122 | 0.01 | 100 | 0 | 0.041 | 0.97265056 | 5.14E-09 | 4 |
| 75 | H3122 | 0.01 | 86.2713869 | 72 | 0.041 | 0.97265056 | 5.14E-09 | 4 |
| 76 | H3122 | 0.03 | 100 | 0 | 0.041 | 0.97265056 | 5.14E-09 | 4 |
| 76 | H3122 | 0.03 | 58.51573 | 72 | 0.041 | 0.97265056 | 5.14E-09 | 4 |
| 77 | H3122 | 0.1 | 100 | 0 | 0.041 | 0.97265056 | 5.14E-09 | 4 |
| 77 | H3122 | 0.1 | 16.447469 | 72 | 0.041 | 0.97265056 | 5.14E-09 | 4 |
| 78 | H3122 | 0.3 | 100 | 0 | 0.041 | 0.97265056 | 5.14E-09 | 4 |
| 78 | H3122 | 0.3 | 8.20724438 | 72 | 0.041 | 0.97265056 | 5.14E-09 | 4 |
| 79 | H3122 | 1 | 100 | 0 | 0.041 | 0.97265056 | 5.14E-09 | 4 |
| 79 | H3122 | 1 | 2.73494373 | 72 | 0.041 | 0.97265056 | 5.14E-09 | 4 |
| 80 | A925 | 0.01 | 100 | 0 | 0.62 | 0.88 | 8.98E-10 | 5 |
| 80 | A925 | 0.01 | 95.9961283 | 72 | 0.62 | 0.88 | 8.98E-10 | 5 |
| 81 | A925 | 0.03 | 100 | 0 | 0.62 | 0.88 | 8.98E-10 | 5 |
| 81 | A925 | 0.03 | 93.9355583 | 72 | 0.62 | 0.88 | 8.98E-10 | 5 |
| 82 | A925 | 0.1 | 100 | 0 | 0.62 | 0.88 | 8.98E-10 | 5 |
| 82 | A925 | 0.1 | 86.3522 | 72 | 0.62 | 0.88 | 8.98E-10 | 5 |
| 83 | A925 | 0.3 | 100 | 0 | 0.62 | 0.88 | 8.98E-10 | 5 |
| 83 | A925 | 0.3 | 64.0991754 | 72 | 0.62 | 0.88 | 8.98E-10 | 5 |
| 84 | A925 | 1 | 100 | 0 | 0.62 | 0.88 | 8.98E-10 | 5 |
| 84 | A925 | 1 | 40.8726244 | 72 | 0.62 | 0.88 | 8.98E-10 | 5 |
| 85 | A925 | 3 | 100 | 0 | 0.62 | 0.88 | 8.98E-10 | 5 |
| 85 | A925 | 3 | 25.7971446 | 72 | 0.62 | 0.88 | 8.98E-10 | 5 |
| 86 | A925 | 10 | 100 | 0 | 0.62 | 0.88 | 8.98E-10 | 5 |
| 86 | A925 | 10 | 12.3466672 | 72 | 0.62 | 0.88 | 8.98E-10 | 5 |
| 87 | H3122 | 0.01 | 100 | 0 | 0.1 | 0.82 | 6.43E-09 | 6 |
| 87 | H3122 | 0.01 | 90.5349794 | 72 | 0.1 | 0.82 | 6.43E-09 | 6 |
| 88 | H3122 | 0.1 | 100 | 0 | 0.1 | 0.82 | 6.43E-09 | 6 |
| 88 | H3122 | 0.1 | 50 | 72 | 0.1 | 0.82 | 6.43E-09 | 6 |
| 89 | H3122 | 1 | 100 | 0 | 0.1 | 0.82 | 6.43E-09 | 6 |
| 89 | H3122 | 1 | 18.1069959 | 72 | 0.1 | 0.82 | 6.43E-09 | 6 |
| 90 | H3122 | 0.01 | 100 | 0 | 0.065 | 0.88 | 1.29E-08 | 7 |
| 90 | H3122 | 0.01 | 101.857585 | 72 | 0.065 | 0.88 | 1.29E-08 | 7 |
| 91 | H3122 | 0.05 | 100 | 0 | 0.065 | 0.88 | 1.29E-08 | 7 |
| 91 | H3122 | 0.05 | 72.4458204 | 72 | 0.065 | 0.88 | 1.29E-08 | 7 |
| 92 | H3122 | 0.1 | 100 | 0 | 0.065 | 0.88 | 1.29E-08 | 7 |
| 92 | H3122 | 0.1 | 54.4891641 | 72 | 0.065 | 0.88 | 1.29E-08 | 7 |
| 93 | H3122 | 0.5 | 100 | 0 | 0.065 | 0.88 | 1.29E-08 | 7 |
| 93 | H3122 | 0.5 | 24.4582043 | 72 | 0.065 | 0.88 | 1.29E-08 | 7 |
| 94 | H3122 | 1 | 100 | 0 | 0.065 | 0.88 | 1.29E-08 | 7 |
| 94 | H3122 | 1 | 24.4582043 | 72 | 0.065 | 0.88 | 1.29E-08 | 7 |
| 95 | H3122 | 5 | 100 | 0 | 0.065 | 0.88 | 1.29E-08 | 7 |
| 95 | H3122 | 5 | 13.622291 | 72 | 0.065 | 0.88 | 1.29E-08 | 7 |
| 96 | H3122 | 10 | 100 | 0 | 0.065 | 0.88 | 1.29E-08 | 7 |
| 96 | H3122 | 10 | 11.7647059 | 72 | 0.065 | 0.88 | 1.29E-08 | 7 |
| 97 | H3122 | 0.03 | 100 | 0 | 0.32 | 0.9265 | 1.54E-08 | 8 |
| 97 | H3122 | 0.03 | 99.4785953 | 72 | 0.32 | 0.9265 | 1.54E-08 | 8 |
| 98 | H3122 | 0.1 | 100 | 0 | 0.32 | 0.9265 | 1.54E-08 | 8 |
| 98 | H3122 | 0.1 | 74.4529184 | 72 | 0.32 | 0.9265 | 1.54E-08 | 8 |
| 99 | H3122 | 0.3 | 100 | 0 | 0.32 | 0.9265 | 1.54E-08 | 8 |
| 99 | H3122 | 0.3 | 51.4560434 | 72 | 0.32 | 0.9265 | 1.54E-08 | 8 |
| 100 | H3122 | 1 | 100 | 0 | 0.32 | 0.9265 | 1.54E-08 | 8 |
| 100 | H3122 | 1 | 34.5433885 | 72 | 0.32 | 0.9265 | 1.54E-08 | 8 |
| 101 | H3122 | 3 | 100 | 0 | 0.32 | 0.9265 | 1.54E-08 | 8 |
| 101 | H3122 | 3 | 23.8526256 | 72 | 0.32 | 0.9265 | 1.54E-08 | 8 |
| 102 | H3122 | 10 | 100 | 0 | 0.32 | 0.9265 | 1.54E-08 | 8 |
| 102 | H3122 | 10 | 13.3550403 | 72 | 0.32 | 0.9265 | 1.54E-08 | 8 |
| 103 | H3122 | 30 | 100 | 0 | 0.32 | 0.9265 | 1.54E-08 | 8 |
| 103 | H3122 | 30 | 6.59732086 | 72 | 0.32 | 0.9265 | 1.54E-08 | 8 |
| 104 | A549 | 0.03 | 100 | 0 | 6.33 | 0.8333428 | 3.04E-08 | 8 |
| 104 | A549 | 0.03 | 96.397806 | 72 | 6.33 | 0.8333428 | 3.04E-08 | 8 |
| 105 | A549 | 0.1 | 100 | 0 | 6.33 | 0.8333428 | 3.04E-08 | 8 |
| 105 | A549 | 0.1 | 94.8733638 | 72 | 6.33 | 0.8333428 | 3.04E-08 | 8 |
| 106 | A549 | 0.3 | 100 | 0 | 6.33 | 0.8333428 | 3.04E-08 | 8 |
| 106 | A549 | 0.3 | 95.6469483 | 72 | 6.33 | 0.8333428 | 3.04E-08 | 8 |
| 107 | A549 | 1 | 100 | 0 | 6.33 | 0.8333428 | 3.04E-08 | 8 |
| 107 | A549 | 1 | 83.7691484 | 72 | 6.33 | 0.8333428 | 3.04E-08 | 8 |
| 108 | A549 | 3 | 100 | 0 | 6.33 | 0.8333428 | 3.04E-08 | 8 |
| 108 | A549 | 3 | 79.2320972 | 72 | 6.33 | 0.8333428 | 3.04E-08 | 8 |
| 109 | A549 | 10 | 100 | 0 | 6.33 | 0.8333428 | 3.04E-08 | 8 |
| 109 | A549 | 10 | 42.7407618 | 72 | 6.33 | 0.8333428 | 3.04E-08 | 8 |
| 110 | A549 | 30 | 100 | 0 | 6.33 | 0.8333428 | 3.04E-08 | 8 |
| 110 | A549 | 30 | 16.6657197 | 72 | 6.33 | 0.8333428 | 3.04E-08 | 8 |
| 111 | H1299 | 0.03 | 100 | 0 | 11.21 | 0.8346 | 1.97E-07 | 8 |
| 111 | H1299 | 0.03 | 96.6827648 | 72 | 11.21 | 0.8346 | 1.97E-07 | 8 |
| 112 | H1299 | 0.1 | 100 | 0 | 11.21 | 0.8346 | 1.97E-07 | 8 |
| 112 | H1299 | 0.1 | 98.2325561 | 72 | 11.21 | 0.8346 | 1.97E-07 | 8 |
| 113 | H1299 | 0.3 | 100 | 0 | 11.21 | 0.8346 | 1.97E-07 | 8 |
| 113 | H1299 | 0.3 | 100.681803 | 72 | 11.21 | 0.8346 | 1.97E-07 | 8 |
| 114 | H1299 | 1 | 100 | 0 | 11.21 | 0.8346 | 1.97E-07 | 8 |
| 114 | H1299 | 1 | 88.2471974 | 72 | 11.21 | 0.8346 | 1.97E-07 | 8 |
| 115 | H1299 | 3 | 100 | 0 | 11.21 | 0.8346 | 1.97E-07 | 8 |
| 115 | H1299 | 3 | 81.1927187 | 72 | 11.21 | 0.8346 | 1.97E-07 | 8 |
| 116 | H1299 | 10 | 100 | 0 | 11.21 | 0.8346 | 1.97E-07 | 8 |
| 116 | H1299 | 10 | 51.4158344 | 72 | 11.21 | 0.8346 | 1.97E-07 | 8 |
| 117 | H1299 | 30 | 100 | 0 | 11.21 | 0.8346 | 1.97E-07 | 8 |
| 117 | H1299 | 30 | 15.5442407 | 72 | 11.21 | 0.8346 | 1.97E-07 | 8 |
| 118 | H661 | 0.25 | 100 | 0 | 0.58 | 0.73282443 | 4.96E-09 | 9 |
| 118 | H661 | 0.25 | 85.4961832 | 72 | 0.58 | 0.73282443 | 4.96E-09 | 9 |
| 119 | H661 | 0.5 | 100 | 0 | 0.58 | 0.73282443 | 4.96E-09 | 9 |
| 119 | H661 | 0.5 | 59.5419847 | 72 | 0.58 | 0.73282443 | 4.96E-09 | 9 |
| 120 | H661 | 1 | 100 | 0 | 0.58 | 0.73282443 | 4.96E-09 | 9 |
| 120 | H661 | 1 | 40.4580153 | 72 | 0.58 | 0.73282443 | 4.96E-09 | 9 |
| 121 | H661 | 2 | 100 | 0 | 0.58 | 0.73282443 | 4.96E-09 | 9 |
| 121 | H661 | 2 | 26.7175573 | 72 | 0.58 | 0.73282443 | 4.96E-09 | 9 |
| 122 | H1299 | 0.25 | 100 | 0 | 0.99 | 0.74766355 | 2.14E-07 | 9 |
| 122 | H1299 | 0.25 | 102.803738 | 72 | 0.99 | 0.74766355 | 2.14E-07 | 9 |
| 123 | H1299 | 0.5 | 100 | 0 | 0.99 | 0.74766355 | 2.14E-07 | 9 |
| 123 | H1299 | 0.5 | 65.4205608 | 72 | 0.99 | 0.74766355 | 2.14E-07 | 9 |
| 124 | H1299 | 1 | 100 | 0 | 0.99 | 0.74766355 | 2.14E-07 | 9 |
| 124 | H1299 | 1 | 47.6635514 | 72 | 0.99 | 0.74766355 | 2.14E-07 | 9 |
| 125 | H1299 | 2 | 100 | 0 | 0.99 | 0.74766355 | 2.14E-07 | 9 |
| 125 | H1299 | 2 | 25.2336449 | 72 | 0.99 | 0.74766355 | 2.14E-07 | 9 |
| 126 | H292 | 0.25 | 100 | 0 | 1.35 | 0.70079 | 1.78E-09 | 9 |
| 126 | H292 | 0.25 | 97.6377953 | 72 | 1.35 | 0.70079 | 1.78E-09 | 9 |
| 127 | H292 | 0.5 | 100 | 0 | 1.35 | 0.70079 | 1.78E-09 | 9 |
| 127 | H292 | 0.5 | 73.2283465 | 72 | 1.35 | 0.70079 | 1.78E-09 | 9 |
| 128 | H292 | 1 | 100 | 0 | 1.35 | 0.70079 | 1.78E-09 | 9 |
| 128 | H292 | 1 | 61.4173228 | 72 | 1.35 | 0.70079 | 1.78E-09 | 9 |
| 129 | H292 | 2 | 100 | 0 | 1.35 | 0.70079 | 1.78E-09 | 9 |
| 129 | H292 | 2 | 29.9212598 | 72 | 1.35 | 0.70079 | 1.78E-09 | 9 |

Table S2: Cell viability data for NSCLC cell lines that were treated with crizotinib. Note that the expression “E-x” refers to x10^-x^, Monolix uses this format to express powers. The cell volume is calculated based on the cell micrographs and the number of cells in each well. Conc= concentration.

Note: The data presented is based on the data extracted from these plots using WebPlotDigitizer (https://automeris.io/WebPlotDigitizer). The reference number refers to the original source for the cell viability curves:

1. Katayama R, et al. Two Novel ALK Mutations Mediate Acquired Resistance to the Next-Generation ALK Inhibitor Alectinib. *Clinical Cancer Research*. 20(22):5686-5696 (2014).
2. Arai S, et al. Osimertinib Overcomes Alectinib Resistance Caused by Amphiregulin in a Leptomeningeal Carcinomatosis Model of ALK-Rearranged Lung Cancer. *Journal of Thoracic Oncology*. 15(5):752-765 (2020).
3. Katayama R, et al. P-glycoprotein Mediates Ceritinib Resistance in Anaplastic Lymphoma Kinase-rearranged Non-small Cell Lung Cancer. *EBioMedicine*. 3:54-66 (2016).
4. Nanjo S, et al. In vivo imaging models of bone and brain metastases and pleural carcinomatosis with a novel human EML 4‐ ALK lung cancer cell line. *Cancer Science.* 106(3):244-252 (2015).
5. Fukuda K, et al. Epithelial-to-Mesenchymal Transition Is a Mechanism of ALK Inhibitor Resistance in Lung Cancer Independent of ALK Mutation Status. *Cancer Research*. 79(7):1658-1670 (2019).
6. Yasuda H, Figueiredo-Pontes L, Kobayashi S, Costa D. Preclinical Rationale for Use of the Clinically Available Multitargeted Tyrosine Kinase Inhibitor Crizotinib in ROS1-Translocated Lung Cancer. *Journal of Thoracic Oncology*. 7(7):1086-1090 (2012).
7. Kim S, et al. Heterogeneity of Genetic Changes Associated with Acquired Crizotinib Resistance in ALK-Rearranged Lung Cancer. *Journal of Thoracic Oncology*. 8(4):415-422 (2013).
8. Ji C, et al. Induction of autophagy contributes to crizotinib resistance in ALK-positive lung cancer. *Cancer Biology & Therapy*. 15(5):570-577 (2014).
9. Ding N, You A, Tian W, Gu L, Deng D. Chidamide increases the sensitivity of non-small cell lung cancer to crizotinib by decreasing c-MET mRNA methylation. <<https://www.biorxiv.org/content/10.1101/2020.03.28.012971v1.full.pdf> >

**Table S3: Tumour cell viability data for erlotinib**

| ID | Cell Line | Conc (μM) | Cell Viability (%) | Time (hr) | *IC_50_* (μM) | *I_MAX_* | *V_C(0)_* (L) | Ref |
| --- | --- | --- | --- | --- | --- | --- | --- | --- |
| 1 | PC9 | 0.02 | 100 | 0 | 0.019 | 0.9943662 | 1.14E-08 | 1 |
| 1 | PC9 | 0.02 | 38.3098592 | 72 | 0.019 | 0.9943662 | 1.14E-08 | 1 |
| 2 | PC9 | 0.05 | 100 | 0 | 0.019 | 0.9943662 | 1.14E-08 | 1 |
| 2 | PC9 | 0.05 | 25.0704225 | 72 | 0.019 | 0.9943662 | 1.14E-08 | 1 |
| 3 | PC9 | 0.1 | 100 | 0 | 0.019 | 0.9943662 | 1.14E-08 | 1 |
| 3 | PC9 | 0.1 | 1.4084507 | 72 | 0.019 | 0.9943662 | 1.14E-08 | 1 |
| 4 | PC9 | 0.5 | 100 | 0 | 0.019 | 0.9943662 | 1.14E-08 | 1 |
| 4 | PC9 | 0.5 | 1.4084507 | 72 | 0.019 | 0.9943662 | 1.14E-08 | 1 |
| 5 | PC9 | 1 | 100 | 0 | 0.019 | 0.9943662 | 1.14E-08 | 1 |
| 5 | PC9 | 1 | 0.56338028 | 72 | 0.019 | 0.9943662 | 1.14E-08 | 1 |
| 6 | PC9 | 0.0002 | 100 | 0 | 0.0127 | 0.9009901 | 6.83E-08 | 2 |
| 6 | PC9 | 0.0002 | 99.009901 | 72 | 0.0127 | 0.9009901 | 6.83E-08 | 2 |
| 7 | PC9 | 0.002 | 100 | 0 | 0.0127 | 0.9009901 | 6.83E-08 | 2 |
| 7 | PC9 | 0.002 | 91.3366337 | 72 | 0.0127 | 0.9009901 | 6.83E-08 | 2 |
| 8 | PC9 | 0.02 | 100 | 0 | 0.0127 | 0.9009901 | 6.83E-08 | 2 |
| 8 | PC9 | 0.02 | 43.0693069 | 72 | 0.0127 | 0.9009901 | 6.83E-08 | 2 |
| 9 | PC9 | 0.2 | 100 | 0 | 0.0127 | 0.9009901 | 6.83E-08 | 2 |
| 9 | PC9 | 0.2 | 14.8514852 | 72 | 0.0127 | 0.9009901 | 6.83E-08 | 2 |
| 10 | PC9 | 2 | 100 | 0 | 0.0127 | 0.9009901 | 6.83E-08 | 2 |
| 10 | PC9 | 2 | 9.9009901 | 72 | 0.0127 | 0.9009901 | 6.83E-08 | 2 |
| 11 | H4006 | 0.01 | 100 | 0 | 0.07 | 0.6793103 | 1.39E-09 | 3 |
| 11 | H4006 | 0.01 | 105.517241 | 72 | 0.07 | 0.6793103 | 1.39E-09 | 3 |
| 12 | H4006 | 0.1 | 100 | 0 | 0.07 | 0.6793103 | 1.39E-09 | 3 |
| 12 | H4006 | 0.1 | 41.3793103 | 72 | 0.07 | 0.6793103 | 1.39E-09 | 3 |
| 13 | H4006 | 1 | 100 | 0 | 0.07 | 0.6793103 | 1.39E-09 | 3 |
| 13 | H4006 | 1 | 26.8965517 | 72 | 0.07 | 0.6793103 | 1.39E-09 | 3 |
| 14 | H4006 | 10 | 100 | 0 | 0.07 | 0.6793103 | 1.39E-09 | 3 |
| 14 | H4006 | 10 | 32.0689655 | 72 | 0.07 | 0.6793103 | 1.39E-09 | 3 |
| 15 | HCC827 | 0.01 | 100 | 0 | 1.07 | 0.65862069 | 8.34E-07 | 3 |
| 15 | HCC827 | 0.01 | 88.9655172 | 72 | 1.07 | 0.65862069 | 8.34E-07 | 3 |
| 16 | HCC827 | 0.1 | 100 | 0 | 1.07 | 0.65862069 | 8.34E-07 | 3 |
| 16 | HCC827 | 0.1 | 61.0344828 | 72 | 1.07 | 0.65862069 | 8.34E-07 | 3 |
| 17 | HCC827 | 1 | 100 | 0 | 1.07 | 0.65862069 | 8.34E-07 | 3 |
| 17 | HCC827 | 1 | 53.7931035 | 72 | 1.07 | 0.65862069 | 8.34E-07 | 3 |
| 18 | HCC827 | 10 | 100 | 0 | 1.07 | 0.65862069 | 8.34E-07 | 3 |
| 18 | HCC827 | 10 | 34.137931 | 72 | 1.07 | 0.65862069 | 8.34E-07 | 3 |
| 19 | HCC827 | 0.001 | 100 | 0 | 0.0086 | 0.89429037 | 3.13E-08 | 4 |
| 19 | HCC827 | 0.001 | 85.7925216 | 72 | 0.0086 | 0.88952617 | 3.13E-08 | 4 |
| 20 | HCC827 | 0.003 | 100 | 0 | 0.0086 | 0.88952617 | 3.13E-08 | 4 |
| 20 | HCC827 | 0.003 | 78.1698089 | 72 | 0.0086 | 0.88952617 | 3.13E-08 | 4 |
| 21 | HCC827 | 0.01 | 100 | 0 | 0.0086 | 0.88952617 | 3.13E-08 | 4 |
| 21 | HCC827 | 0.01 | 37.0145609 | 72 | 0.0086 | 0.88952617 | 3.13E-08 | 4 |
| 22 | HCC827 | 0.03 | 100 | 0 | 0.0086 | 0.88952617 | 3.13E-08 | 4 |
| 22 | HCC827 | 0.03 | 17.8523192 | 72 | 0.0086 | 0.88952617 | 3.13E-08 | 4 |
| 23 | HCC827 | 0.1 | 100 | 0 | 0.0086 | 0.88952617 | 3.13E-08 | 4 |
| 23 | HCC827 | 0.1 | 9.40905475 | 72 | 0.0086 | 0.88952617 | 3.13E-08 | 4 |
| 24 | HCC827 | 0.3 | 100 | 0 | 0.0086 | 0.88952617 | 3.13E-08 | 4 |
| 24 | HCC827 | 0.3 | 10.5709634 | 72 | 0.0086 | 0.88952617 | 3.13E-08 | 4 |
| 25 | HCC827 | 1 | 100 | 0 | 0.0086 | 0.88952617 | 3.13E-08 | 4 |
| 25 | HCC827 | 1 | 11.1926215 | 72 | 0.0086 | 0.88952617 | 3.13E-08 | 4 |
| 26 | HCC827 | 3 | 100 | 0 | 0.0086 | 0.88952617 | 3.13E-08 | 4 |
| 26 | HCC827 | 3 | 13.7301338 | 72 | 0.0086 | 0.88952617 | 3.13E-08 | 4 |
| 27 | HCC827 | 10 | 100 | 0 | 0.0086 | 0.88952617 | 3.13E-08 | 4 |
| 27 | HCC827 | 10 | 11.0473829 | 72 | 0.0086 | 0.88952617 | 3.13E-08 | 4 |
| 28 | PC9 | 0.1 | 100 | 0 | 0.6777 | 0.89811711 | 1.25E-08 | 5 |
| 28 | PC9 | 0.1 | 58.7785883 | 72 | 0.6777 | 0.89811711 | 1.25E-08 | 5 |
| 29 | PC9 | 1 | 100 | 0 | 0.6777 | 0.89811711 | 1.25E-08 | 5 |
| 29 | PC9 | 1 | 48.5350946 | 72 | 0.6777 | 0.89811711 | 1.25E-08 | 5 |
| 30 | PC9 | 10 | 100 | 0 | 0.6777 | 0.89811711 | 1.25E-08 | 5 |
| 30 | PC9 | 10 | 25.4416404 | 72 | 0.6777 | 0.89811711 | 1.25E-08 | 5 |
| 31 | PC9 | 100 | 100 | 0 | 0.6777 | 0.89811711 | 1.25E-08 | 5 |
| 31 | PC9 | 100 | 10.1882886 | 72 | 0.6777 | 0.89811711 | 1.25E-08 | 5 |
| 32 | A549 | 1 | 100 | 0 | 11.98 | 0.82269392 | 7.61E-09 | 6 |
| 32 | A549 | 1 | 95.2778499 | 72 | 11.98 | 0.82269392 | 7.61E-09 | 6 |
| 33 | A549 | 2.5 | 100 | 0 | 11.98 | 0.82269392 | 7.61E-09 | 6 |
| 33 | A549 | 2.5 | 89.724268 | 72 | 11.98 | 0.82269392 | 7.61E-09 | 6 |
| 34 | A549 | 5 | 100 | 0 | 11.98 | 0.82269392 | 7.61E-09 | 6 |
| 34 | A549 | 5 | 89.6955744 | 72 | 11.98 | 0.82269392 | 7.61E-09 | 6 |
| 35 | A549 | 10 | 100 | 0 | 11.98 | 0.82269392 | 7.61E-09 | 6 |
| 35 | A549 | 10 | 50.9824149 | 72 | 11.98 | 0.82269392 | 7.61E-09 | 6 |
| 36 | A549 | 25 | 100 | 0 | 11.98 | 0.82269392 | 7.61E-09 | 6 |
| 36 | A549 | 25 | 46.0450627 | 72 | 11.98 | 0.82269392 | 7.61E-09 | 6 |
| 37 | A549 | 50 | 100 | 0 | 11.98 | 0.82269392 | 7.61E-09 | 6 |
| 37 | A549 | 50 | 40.4935303 | 72 | 11.98 | 0.82269392 | 7.61E-09 | 6 |
| 38 | A549 | 100 | 100 | 0 | 11.98 | 0.82269392 | 7.61E-09 | 6 |
| 38 | A549 | 100 | 31.8717805 | 72 | 11.98 | 0.82269392 | 7.61E-09 | 6 |
| 39 | A549 | 125 | 100 | 0 | 11.98 | 0.82269392 | 7.61E-09 | 6 |
| 39 | A549 | 125 | 17.7306079 | 72 | 11.98 | 0.82269392 | 7.61E-09 | 6 |
| 40 | H1975 | 0.3 | 100 | 0 | 4.4 | 0.67138337 | 8.43E-09 | 6 |
| 40 | H1975 | 0.3 | 97.64324 | 72 | 4.4 | 0.67138337 | 8.43E-09 | 6 |
| 41 | H1975 | 0.9 | 100 | 0 | 4.4 | 0.67138337 | 8.43E-09 | 6 |
| 41 | H1975 | 0.9 | 96.749226 | 72 | 4.4 | 0.67138337 | 8.43E-09 | 6 |
| 42 | H1975 | 1.5 | 100 | 0 | 4.4 | 0.67138337 | 8.43E-09 | 6 |
| 42 | H1975 | 1.5 | 73.5655542 | 72 | 4.4 | 0.67138337 | 8.43E-09 | 6 |
| 43 | H1975 | 3.5 | 100 | 0 | 4.4 | 0.67138337 | 8.43E-09 | 6 |
| 43 | H1975 | 3.5 | 62.8019258 | 72 | 4.4 | 0.67138337 | 8.43E-09 | 6 |
| 44 | H1975 | 5 | 100 | 0 | 4.4 | 0.67138337 | 8.43E-09 | 6 |
| 44 | H1975 | 5 | 47.5757287 | 72 | 4.4 | 0.67138337 | 8.43E-09 | 6 |
| 45 | H1975 | 10 | 100 | 0 | 4.4 | 0.67138337 | 8.43E-09 | 6 |
| 45 | H1975 | 10 | 34.9054159 | 72 | 4.4 | 0.67138337 | 8.43E-09 | 6 |
| 46 | H1975 | 25 | 100 | 0 | 4.4 | 0.67138337 | 8.43E-09 | 6 |
| 46 | H1975 | 25 | 35.6023513 | 72 | 4.4 | 0.67138337 | 8.43E-09 | 6 |
| 47 | H1975 | 50 | 100 | 0 | 4.4 | 0.67138337 | 8.43E-09 | 6 |
| 47 | H1975 | 50 | 31.5237108 | 72 | 4.4 | 0.67138337 | 8.43E-09 | 6 |
| 48 | H1975 | 100 | 100 | 0 | 4.4 | 0.67138337 | 8.43E-09 | 6 |
| 48 | H1975 | 100 | 32.8616631 | 72 | 4.4 | 0.67138337 | 8.43E-09 | 6 |
| 49 | H1993 | 0.75 | 100 | 0 | 53.392 | 0.69497893 | 3.40E-09 | 6 |
| 49 | H1993 | 0.75 | 89.2945197 | 72 | 53.392 | 0.69497893 | 3.40E-09 | 6 |
| 50 | H1993 | 1.5 | 100 | 0 | 53.392 | 0.69497893 | 3.40E-09 | 6 |
| 50 | H1993 | 1.5 | 90.112449 | 72 | 53.392 | 0.69497893 | 3.40E-09 | 6 |
| 51 | H1993 | 2.5 | 100 | 0 | 53.392 | 0.69497893 | 3.40E-09 | 6 |
| 51 | H1993 | 2.5 | 76.9531811 | 72 | 53.392 | 0.69497893 | 3.40E-09 | 6 |
| 52 | H1993 | 5 | 100 | 0 | 53.392 | 0.69497893 | 3.40E-09 | 6 |
| 52 | H1993 | 5 | 66.5249097 | 72 | 53.392 | 0.69497893 | 3.40E-09 | 6 |
| 53 | H1993 | 10 | 100 | 0 | 53.392 | 0.69497893 | 3.40E-09 | 6 |
| 53 | H1993 | 10 | 60.3565232 | 72 | 53.392 | 0.69497893 | 3.40E-09 | 6 |
| 54 | H1993 | 25 | 100 | 0 | 53.392 | 0.69497893 | 3.40E-09 | 6 |
| 54 | H1993 | 25 | 52.9631997 | 72 | 53.392 | 0.69497893 | 3.40E-09 | 6 |
| 55 | H1993 | 50 | 100 | 0 | 53.392 | 0.69497893 | 3.40E-09 | 6 |
| 55 | H1993 | 50 | 50.1474099 | 72 | 53.392 | 0.69497893 | 3.40E-09 | 6 |
| 56 | H1993 | 100 | 100 | 0 | 53.392 | 0.69497893 | 3.40E-09 | 6 |
| 56 | H1993 | 100 | 40.3224754 | 72 | 53.392 | 0.69497893 | 3.40E-09 | 6 |
| 57 | H1993 | 125 | 100 | 0 | 53.392 | 0.69497893 | 3.40E-09 | 6 |
| 57 | H1993 | 125 | 30.5021068 | 72 | 53.392 | 0.69497893 | 3.40E-09 | 6 |
| 58 | HCC827 | 0.005 | 100 | 0 | 0.2 | 0.66026411 | 3.13E-08 | 7 |
| 58 | HCC827 | 0.005 | 76.5906363 | 72 | 0.2 | 0.66026411 | 3.13E-08 | 7 |
| 59 | HCC827 | 0.01 | 100 | 0 | 0.2 | 0.66026411 | 3.13E-08 | 7 |
| 59 | HCC827 | 0.01 | 67.2869148 | 72 | 0.2 | 0.66026411 | 3.13E-08 | 7 |
| 60 | HCC827 | 0.2 | 100 | 0 | 0.2 | 0.66026411 | 3.13E-08 | 7 |
| 60 | HCC827 | 0.2 | 50 | 72 | 0.2 | 0.66026411 | 3.13E-08 | 7 |
| 61 | HCC827 | 1 | 100 | 0 | 0.2 | 0.66026411 | 3.13E-08 | 7 |
| 61 | HCC827 | 1 | 33.9735894 | 72 | 0.2 | 0.66026411 | 3.13E-08 | 7 |

Table S3: Cell viability data for NSCLC cell lines that were treated with erlotinib. Note that the expression “E-x” refers to x10^-x^, Monolix uses this format to express powers. The cell volume is calculated based on the cell micrographs and the number of cells in each well. Conc= Concentration.

Note: The data presented is based on the data extracted from these plots using WebPlotDigitizer (https://automeris.io/WebPlotDigitizer). The reference number refers to the original source for the cell viability curves:

1. Chen J, Cui J, Guo X, Cao X, Li Q. Increased expression of miR-641 contributes to erlotinib resistance in non-small-cell lung cancer cells by targeting NF1. *Cancer Medicine*. 7(4):1394-1403 (2018).
2. Chin T, et al. Reduced Erlotinib Sensitivity of Epidermal Growth Factor Receptor-Mutant Non–Small Cell Lung Cancer following Cisplatin Exposure: A Cell Culture Model of Second-line Erlotinib Treatment. *Clinical Cancer Research*. 14(21):6867-6876 (2008).
3. Xiao Z, et al. Metformin Triggers Autophagy to Attenuate Drug-Induced Apoptosis in NSCLC Cells, with Minor Effects on Tumors of Diabetic Patients. *Neoplasia*. 19(5):385-395 (2017).
4. Wilson C, et al. Overcoming EMT-associated resistance to anti-cancer drugs via Src/FAK pathway inhibition. *Oncotarget*. 5(17):7328-7341 (2014).
5. Shintani T, et al. Eukaryotic translation initiation factor 3 subunit C is associated with acquired resistance to erlotinib in non-small cell lung cancer. *Oncotarget*. 9(101):37520-37533 (2018).
6. Dermawan J, et al. Quinacrine Overcomes Resistance to Erlotinib by Inhibiting FACT, NF-κB, and Cell-Cycle Progression in Non–Small Cell Lung Cancer. *Molecular Cancer Therapeutics*. 13(9):2203-2214 (2014).
7. Kim T, et al. DNA Polymerase Alpha Subunit B Is a Binding Protein for Erlotinib Resistance in Non-Small Cell Lung Cancer. *Cancers*. 12(9):2613 (2020).

**Table S4: Tumour cell viability data for gefitinib**

| ID | Cell  Line | Conc (μM) | Cell Viability (%) | Time (hr) | *IC_50_* (μM) | *I_MAX_* | *V_C(0)_* (L) | Ref |
| --- | --- | --- | --- | --- | --- | --- | --- | --- |
| 1 | H292 | 0.001 | 100 | 0 | 0.149 | 0.98282209 | 3.43E-09 | 1 |
| 1 | H292 | 0.001 | 85.6441718 | 72 | 0.149 | 0.98282209 | 3.43E-09 | 1 |
| 2 | H292 | 0.01 | 100 | 0 | 0.149 | 0.98282209 | 3.43E-09 | 1 |
| 2 | H292 | 0.01 | 69.4478528 | 72 | 0.149 | 0.98282209 | 3.43E-09 | 1 |
| 3 | H292 | 0.1 | 100 | 0 | 0.149 | 0.98282209 | 3.43E-09 | 1 |
| 3 | H292 | 0.1 | 50.797546 | 72 | 0.149 | 0.98282209 | 3.43E-09 | 1 |
| 4 | H292 | 1 | 100 | 0 | 0.149 | 0.98282209 | 3.43E-09 | 1 |
| 4 | H292 | 1 | 45.8895706 | 72 | 0.149 | 0.98282209 | 3.43E-09 | 1 |
| 5 | H292 | 10 | 100 | 0 | 0.149 | 0.98282209 | 3.43E-09 | 1 |
| 5 | H292 | 10 | 16.4417178 | 72 | 0.149 | 0.98282209 | 3.43E-09 | 1 |
| 6 | H292 | 50 | 100 | 0 | 0.149 | 0.98282209 | 3.43E-09 | 1 |
| 6 | H292 | 50 | 1.71779141 | 72 | 0.149 | 0.98282209 | 3.43E-09 | 1 |
| 7 | HCC827 | 0.001 | 100 | 0 | 0.0075 | 0.9413 | 1.30E-07 | 1 |
| 7 | HCC827 | 0.001 | 87.3619632 | 72 | 0.0075 | 0.9413 | 1.30E-07 | 1 |
| 8 | HCC827 | 0.01 | 100 | 0 | 0.0075 | 0.9413 | 1.30E-07 | 1 |
| 8 | HCC827 | 0.01 | 45.6441718 | 72 | 0.0075 | 0.9413 | 1.30E-07 | 1 |
| 9 | HCC827 | 0.1 | 100 | 0 | 0.0075 | 0.9413 | 1.30E-07 | 1 |
| 9 | HCC827 | 0.1 | 30.6748466 | 72 | 0.0075 | 0.9413 | 1.30E-07 | 1 |
| 10 | HCC827 | 1 | 100 | 0 | 0.0075 | 0.9413 | 1.30E-07 | 1 |
| 10 | HCC827 | 1 | 15.4601227 | 72 | 0.0075 | 0.9413 | 1.30E-07 | 1 |
| 11 | HCC827 | 10 | 100 | 0 | 0.0075 | 0.9413 | 1.30E-07 | 1 |
| 11 | HCC827 | 10 | 8.09815951 | 72 | 0.0075 | 0.9413 | 1.30E-07 | 1 |
| 12 | HCC827 | 50 | 100 | 0 | 0.0075 | 0.9413 | 1.30E-07 | 1 |
| 12 | HCC827 | 50 | 5.87 | 72 | 0.0075 | 0.9413 | 1.30E-07 | 1 |
| 13 | PC9 | 0.00625 | 100 | 0 | 0.01 | 0.81169341 | 9.10E-09 | 2 |
| 13 | PC9 | 0.00625 | 66.9546172 | 72 | 0.01 | 0.81169341 | 9.10E-09 | 2 |
| 14 | PC9 | 0.0125 | 100 | 0 | 0.01 | 0.81169341 | 9.10E-09 | 2 |
| 14 | PC9 | 0.0125 | 46.251407 | 72 | 0.01 | 0.81169341 | 9.10E-09 | 2 |
| 15 | PC9 | 0.025 | 100 | 0 | 0.01 | 0.81169341 | 9.10E-09 | 2 |
| 15 | PC9 | 0.025 | 38.7716726 | 72 | 0.01 | 0.81169341 | 9.10E-09 | 2 |
| 16 | PC9 | 0.05 | 100 | 0 | 0.01 | 0.81169341 | 9.10E-09 | 2 |
| 16 | PC9 | 0.05 | 31.0419448 | 72 | 0.01 | 0.81169341 | 9.10E-09 | 2 |
| 17 | PC9 | 0.1 | 100 | 0 | 0.01 | 0.81169341 | 9.10E-09 | 2 |
| 17 | PC9 | 0.1 | 26.5084684 | 72 | 0.01 | 0.81169341 | 9.10E-09 | 2 |
| 18 | PC9 | 0.2 | 100 | 0 | 0.01 | 0.81169341 | 9.10E-09 | 2 |
| 18 | PC9 | 0.2 | 24.1834856 | 72 | 0.01 | 0.81169341 | 9.10E-09 | 2 |
| 19 | PC9 | 0.4 | 100 | 0 | 0.01 | 0.81169341 | 9.10E-09 | 2 |
| 19 | PC9 | 0.4 | 18.8306589 | 72 | 0.01 | 0.81169341 | 9.10E-09 | 2 |
| 20 | HCC827 | 0.001 | 100 | 0 | 0.0052 | 0.75260664 | 1.04E-07 | 3 |
| 20 | HCC827 | 0.001 | 71.6587678 | 72 | 0.0052 | 0.75260664 | 1.04E-07 | 3 |
| 21 | HCC827 | 0.005 | 100 | 0 | 0.0052 | 0.75260664 | 1.04E-07 | 3 |
| 21 | HCC827 | 0.005 | 63.6966825 | 72 | 0.0052 | 0.75260664 | 1.04E-07 | 3 |
| 22 | HCC827 | 0.01 | 100 | 0 | 0.0052 | 0.75260664 | 1.04E-07 | 3 |
| 22 | HCC827 | 0.01 | 31.2796209 | 72 | 0.0052 | 0.75260664 | 1.04E-07 | 3 |
| 23 | HCC827 | 0.05 | 100 | 0 | 0.0052 | 0.75260664 | 1.04E-07 | 3 |
| 23 | HCC827 | 0.05 | 27.5829384 | 72 | 0.0052 | 0.75260664 | 1.04E-07 | 3 |
| 24 | HCC827 | 0.1 | 100 | 0 | 0.0052 | 0.75260664 | 1.04E-07 | 3 |
| 24 | HCC827 | 0.1 | 26.7298578 | 72 | 0.0052 | 0.75260664 | 1.04E-07 | 3 |
| 25 | HCC827 | 0.5 | 100 | 0 | 0.0052 | 0.75260664 | 1.04E-07 | 3 |
| 25 | HCC827 | 0.5 | 27.014218 | 72 | 0.0052 | 0.75260664 | 1.04E-07 | 3 |
| 26 | HCC827 | 1 | 100 | 0 | 0.0052 | 0.75260664 | 1.04E-07 | 3 |
| 26 | HCC827 | 1 | 24.7393365 | 72 | 0.0052 | 0.75260664 | 1.04E-07 | 3 |
| 27 | HCC827 | 0.005 | 100 | 0 | 0.024 | 0.83458647 | 6.25E-08 | 4 |
| 27 | HCC827 | 0.005 | 85.7142857 | 72 | 0.024 | 0.83458647 | 6.25E-08 | 4 |
| 28 | HCC827 | 0.01 | 100 | 0 | 0.024 | 0.83458647 | 6.25E-08 | 4 |
| 28 | HCC827 | 0.01 | 64.6616541 | 72 | 0.024 | 0.83458647 | 6.25E-08 | 4 |
| 29 | HCC827 | 0.05 | 100 | 0 | 0.024 | 0.83458647 | 6.25E-08 | 4 |
| 29 | HCC827 | 0.05 | 28.5714286 | 72 | 0.024 | 0.83458647 | 6.25E-08 | 4 |
| 30 | HCC827 | 0.1 | 100 | 0 | 0.024 | 0.83458647 | 6.25E-08 | 4 |
| 30 | HCC827 | 0.1 | 22.556391 | 72 | 0.024 | 0.83458647 | 6.25E-08 | 4 |
| 31 | HCC827 | 0.5 | 100 | 0 | 0.024 | 0.83458647 | 6.25E-08 | 4 |
| 31 | HCC827 | 0.5 | 23.3082707 | 72 | 0.024 | 0.83458647 | 6.25E-08 | 4 |
| 32 | HCC827 | 1 | 100 | 0 | 0.024 | 0.83458647 | 6.25E-08 | 4 |
| 32 | HCC827 | 1 | 20.3007519 | 72 | 0.024 | 0.83458647 | 6.25E-08 | 4 |
| 33 | HCC827 | 5 | 100 | 0 | 0.024 | 0.83458647 | 6.25E-08 | 4 |
| 33 | HCC827 | 5 | 16.5413534 | 72 | 0.024 | 0.83458647 | 6.25E-08 | 4 |
| 34 | PC9 | 0.005 | 100 | 0 | 0.029 | 0.87218045 | 1.37E-08 | 4 |
| 34 | PC9 | 0.005 | 90.9774436 | 72 | 0.029 | 0.87218045 | 1.37E-08 | 4 |
| 35 | PC9 | 0.01 | 100 | 0 | 0.029 | 0.87218045 | 1.37E-08 | 4 |
| 35 | PC9 | 0.01 | 68.4210526 | 72 | 0.029 | 0.87218045 | 1.37E-08 | 4 |
| 36 | PC9 | 0.05 | 100 | 0 | 0.029 | 0.87218045 | 1.37E-08 | 4 |
| 36 | PC9 | 0.05 | 42.1052632 | 72 | 0.029 | 0.87218045 | 1.37E-08 | 4 |
| 37 | PC9 | 0.1 | 100 | 0 | 0.029 | 0.87218045 | 1.37E-08 | 4 |
| 37 | PC9 | 0.1 | 25.5639098 | 72 | 0.029 | 0.87218045 | 1.37E-08 | 4 |
| 38 | PC9 | 0.5 | 100 | 0 | 0.029 | 0.87218045 | 1.37E-08 | 4 |
| 38 | PC9 | 0.5 | 18.7969925 | 72 | 0.029 | 0.87218045 | 1.37E-08 | 4 |
| 39 | PC9 | 1 | 100 | 0 | 0.029 | 0.87218045 | 1.37E-08 | 4 |
| 39 | PC9 | 1 | 18.0451128 | 72 | 0.029 | 0.87218045 | 1.37E-08 | 4 |
| 40 | PC9 | 5 | 100 | 0 | 0.029 | 0.87218045 | 1.37E-08 | 4 |
| 40 | PC9 | 5 | 12.7819549 | 72 | 0.029 | 0.87218045 | 1.37E-08 | 4 |
| 41 | A549 | 5 | 100 | 0 | 8.42 | 0.75263158 | 2.54E-08 | 5 |
| 41 | A549 | 5 | 63.6842105 | 72 | 8.42 | 0.75263158 | 2.54E-08 | 5 |
| 42 | A549 | 10 | 100 | 0 | 8.42 | 0.75263158 | 2.54E-08 | 5 |
| 42 | A549 | 10 | 49.4736842 | 72 | 8.42 | 0.75263158 | 2.54E-08 | 5 |
| 43 | A549 | 15 | 100 | 0 | 8.42 | 0.75263158 | 2.54E-08 | 5 |
| 43 | A549 | 15 | 41.5789474 | 72 | 8.42 | 0.75263158 | 2.54E-08 | 5 |
| 44 | A549 | 20 | 100 | 0 | 8.42 | 0.75263158 | 2.54E-08 | 5 |
| 44 | A549 | 20 | 32.1052632 | 72 | 8.42 | 0.75263158 | 2.54E-08 | 5 |
| 45 | A549 | 25 | 100 | 0 | 8.42 | 0.75263158 | 2.54E-08 | 5 |
| 45 | A549 | 25 | 29.4736842 | 72 | 8.42 | 0.75263158 | 2.54E-08 | 5 |
| 46 | A549 | 30 | 100 | 0 | 8.42 | 0.75263158 | 2.54E-08 | 5 |
| 46 | A549 | 30 | 25.2631579 | 72 | 8.42 | 0.75263158 | 2.54E-08 | 5 |
| 47 | A549 | 40 | 100 | 0 | 8.42 | 0.75263158 | 2.54E-08 | 5 |
| 47 | A549 | 40 | 24.7368421 | 72 | 8.42 | 0.75263158 | 2.54E-08 | 5 |
| 48 | H1650 | 5 | 100 | 0 | 5.26 | 0.81052632 | 2.65E-10 | 5 |
| 48 | H1650 | 5 | 53.6842105 | 72 | 5.26 | 0.81052632 | 2.65E-10 | 5 |
| 49 | H1650 | 10 | 100 | 0 | 5.26 | 0.81052632 | 2.65E-10 | 5 |
| 49 | H1650 | 10 | 43.1578947 | 72 | 5.26 | 0.81052632 | 2.65E-10 | 5 |
| 50 | H1650 | 15 | 100 | 0 | 5.26 | 0.81052632 | 2.65E-10 | 5 |
| 50 | H1650 | 15 | 35.2631579 | 72 | 5.26 | 0.81052632 | 2.65E-10 | 5 |
| 51 | H1650 | 20 | 100 | 0 | 5.26 | 0.81052632 | 2.65E-10 | 5 |
| 51 | H1650 | 20 | 28.4210526 | 72 | 5.26 | 0.81052632 | 2.65E-10 | 5 |
| 52 | H1650 | 25 | 100 | 0 | 5.26 | 0.81052632 | 2.65E-10 | 5 |
| 52 | H1650 | 25 | 23.1578947 | 72 | 5.26 | 0.81052632 | 2.65E-10 | 5 |
| 53 | H1650 | 30 | 100 | 0 | 5.26 | 0.81052632 | 2.65E-10 | 5 |
| 53 | H1650 | 30 | 20 | 72 | 5.26 | 0.81052632 | 2.65E-10 | 5 |
| 54 | H1650 | 40 | 100 | 0 | 5.26 | 0.81052632 | 2.65E-10 | 5 |
| 54 | H1650 | 40 | 18.9473684 | 72 | 5.26 | 0.81052632 | 2.65E-10 | 5 |
| 55 | PC9 | 0.01 | 100 | 0 | 0.103 | 0.971 | 4.55E-08 | 6 |
| 55 | PC9 | 0.01 | 98.0563075 | 72 | 0.103 | 0.971 | 4.55E-08 | 6 |
| 56 | PC9 | 0.025 | 100 | 0 | 0.103 | 0.971 | 4.55E-08 | 6 |
| 56 | PC9 | 0.025 | 88.8467786 | 72 | 0.103 | 0.971 | 4.55E-08 | 6 |
| 57 | PC9 | 0.05 | 100 | 0 | 0.103 | 0.971 | 4.55E-08 | 6 |
| 57 | PC9 | 0.05 | 67.3362209 | 72 | 0.103 | 0.971 | 4.55E-08 | 6 |
| 58 | PC9 | 0.1 | 100 | 0 | 0.103 | 0.971 | 4.55E-08 | 6 |
| 58 | PC9 | 0.1 | 50.5035192 | 72 | 0.103 | 0.971 | 4.55E-08 | 6 |
| 59 | PC9 | 0.25 | 100 | 0 | 0.103 | 0.971 | 4.55E-08 | 6 |
| 59 | PC9 | 0.25 | 27.2604223 | 72 | 0.103 | 0.971 | 4.55E-08 | 6 |
| 60 | PC9 | 0.5 | 100 | 0 | 0.103 | 0.971 | 4.55E-08 | 6 |
| 60 | PC9 | 0.5 | 15.0839199 | 72 | 0.103 | 0.971 | 4.55E-08 | 6 |
| 61 | PC9 | 1 | 100 | 0 | 0.103 | 0.971 | 4.55E-08 | 6 |
| 61 | PC9 | 1 | 7.61234434 | 72 | 0.103 | 0.971 | 4.55E-08 | 6 |
| 62 | PC9 | 5 | 100 | 0 | 0.103 | 0.971 | 4.55E-08 | 6 |
| 62 | PC9 | 5 | 3.80075799 | 72 | 0.103 | 0.971 | 4.55E-08 | 6 |
| 63 | PC9 | 10 | 100 | 0 | 0.103 | 0.971 | 4.55E-08 | 6 |
| 63 | PC9 | 10 | 2.9 | 72 | 0.103 | 0.971 | 4.55E-08 | 6 |
| 64 | H292 | 0.01 | 100 | 0 | 0.259 | 0.88846779 | 5.48E-09 | 6 |
| 64 | H292 | 0.01 | 98.8521927 | 72 | 0.259 | 0.88846779 | 5.48E-09 | 6 |
| 65 | H292 | 0.025 | 100 | 0 | 0.259 | 0.88846779 | 5.48E-09 | 6 |
| 65 | H292 | 0.025 | 87.3037358 | 72 | 0.259 | 0.88846779 | 5.48E-09 | 6 |
| 66 | H292 | 0.05 | 100 | 0 | 0.259 | 0.88846779 | 5.48E-09 | 6 |
| 66 | H292 | 0.05 | 82.7395777 | 72 | 0.259 | 0.88846779 | 5.48E-09 | 6 |
| 67 | H292 | 0.1 | 100 | 0 | 0.259 | 0.88846779 | 5.48E-09 | 6 |
| 67 | H292 | 0.1 | 74.6670276 | 72 | 0.259 | 0.88846779 | 5.48E-09 | 6 |
| 68 | H292 | 0.25 | 100 | 0 | 0.259 | 0.88846779 | 5.48E-09 | 6 |
| 68 | H292 | 0.25 | 51.4347591 | 72 | 0.259 | 0.88846779 | 5.48E-09 | 6 |
| 69 | H292 | 0.5 | 100 | 0 | 0.259 | 0.88846779 | 5.48E-09 | 6 |
| 69 | H292 | 0.5 | 41.6188414 | 72 | 0.259 | 0.88846779 | 5.48E-09 | 6 |
| 70 | H292 | 1 | 100 | 0 | 0.259 | 0.88846779 | 5.48E-09 | 6 |
| 70 | H292 | 1 | 30.0487277 | 72 | 0.259 | 0.88846779 | 5.48E-09 | 6 |
| 71 | H292 | 5 | 100 | 0 | 0.259 | 0.88846779 | 5.48E-09 | 6 |
| 71 | H292 | 5 | 18.624797 | 72 | 0.259 | 0.88846779 | 5.48E-09 | 6 |
| 72 | H292 | 10 | 100 | 0 | 0.259 | 0.88846779 | 5.48E-09 | 6 |
| 72 | H292 | 10 | 11.1532214 | 72 | 0.259 | 0.88846779 | 5.48E-09 | 6 |
| 73 | PC9 | 0.001 | 100 | 0 | 0.047 | 0.90871369 | 1.14E-09 | 7 |
| 73 | PC9 | 0.001 | 100.414938 | 72 | 0.047 | 0.90871369 | 1.14E-09 | 7 |
| 74 | PC9 | 0.01 | 100 | 0 | 0.047 | 0.90871369 | 1.14E-09 | 7 |
| 74 | PC9 | 0.01 | 89.626556 | 72 | 0.047 | 0.90871369 | 1.14E-09 | 7 |
| 75 | PC9 | 0.1 | 100 | 0 | 0.047 | 0.90871369 | 1.14E-09 | 7 |
| 75 | PC9 | 0.1 | 34.439834 | 72 | 0.047 | 0.90871369 | 1.14E-09 | 7 |
| 76 | PC9 | 1 | 100 | 0 | 0.047 | 0.90871369 | 1.14E-09 | 7 |
| 76 | PC9 | 1 | 33.6099585 | 72 | 0.047 | 0.90871369 | 1.14E-09 | 7 |
| 77 | PC9 | 10 | 100 | 0 | 0.047 | 0.90871369 | 1.14E-09 | 7 |
| 77 | PC9 | 10 | 9.12863071 | 72 | 0.047 | 0.90871369 | 1.14E-09 | 7 |
| 78 | HCC827 | 0.001 | 100 | 0 | 0.0204 | 0.88796681 | 5.21E-09 | 7 |
| 78 | HCC827 | 0.001 | 107.883817 | 72 | 0.0204 | 0.88796681 | 5.21E-09 | 7 |
| 79 | HCC827 | 0.01 | 100 | 0 | 0.0204 | 0.88796681 | 5.21E-09 | 7 |
| 79 | HCC827 | 0.01 | 61.8257261 | 72 | 0.0204 | 0.88796681 | 5.21E-09 | 7 |
| 80 | HCC827 | 0.1 | 100 | 0 | 0.0204 | 0.88796681 | 5.21E-09 | 7 |
| 80 | HCC827 | 0.1 | 31.5352697 | 72 | 0.0204 | 0.88796681 | 5.21E-09 | 7 |
| 81 | HCC827 | 1 | 100 | 0 | 0.0204 | 0.88796681 | 5.21E-09 | 7 |
| 81 | HCC827 | 1 | 24.8962656 | 72 | 0.0204 | 0.88796681 | 5.21E-09 | 7 |
| 82 | HCC827 | 10 | 100 | 0 | 0.0204 | 0.88796681 | 5.21E-09 | 7 |
| 82 | HCC827 | 10 | 11.2033195 | 72 | 0.0204 | 0.88796681 | 5.21E-09 | 7 |

Table S4: Cell viability data for NSCLC cell lines that were treated with gefitinib. Note that the expression “E-x” refers to x10^-x^, Monolix uses this format to express powers. The cell volume is calculated based on the cell micrographs and the number of cells in each well.

Note: The data presented is based on the data extracted from these plots using WebPlotDigitizer (https://automeris.io/WebPlotDigitizer). The reference number refers to the original source for the cell viability curves:

1. Han S, Ding H, Zhao W, Teng F, Li P. Enhancement of gefitinib-induced growth inhibition by Marsdenia tenacissima extract in non-small cell lung cancer cells expressing wild or mutant EGFR. *BMC Complementary and Alternative Medicine*. 14(1) (2014).
2. Shen H, et al. Alteration in Mir-21/PTEN Expression Modulates Gefitinib Resistance in Non-Small Cell Lung Cancer. *PLoS ONE*. 9(7):e103305 (2014).
3. Hong S, et al. Cucurbitacin D Overcomes Gefitinib Resistance by Blocking EGF Binding to EGFR and Inducing Cell Death in NSCLCs. *Frontiers in Oncology*. 10 (2020).
4. Xiao Y, et al. FBXW 7 deletion contributes to lung tumor development and confers resistance to gefitinib therapy. *Molecular Oncology*. 12(6):883-895 (2018).
5. Yao W, et al. All-trans retinoic acid reduces cancer stem cell-like cell-mediated resistance to gefitinib in NSCLC adenocarcinoma cells. *BMC Cancer*. 20(1) (2020).
6. Zhao H, Huang Y, Shi J, Dai Y, Wu L, Zhou H. ABCC10 Plays a Significant Role in the Transport of Gefitinib and Contributes to Acquired Resistance to Gefitinib in NSCLC. *Frontiers in Pharmacology*. 9 (2018).
7. Terai H, et al. Activation of the FGF2-FGFR1 Autocrine Pathway: A Novel Mechanism of Acquired Resistance to Gefitinib in NSCLC. *Molecular Cancer Research*. 11(7):759-767 (2013).

**Table S5:** **Summary of electron micrographs for NSCLC cell lines**

| Cell Line | Estimated Radius (μM) | Estimated Volume (μM^3^) | Ref |
| --- | --- | --- | --- |
| A549 | 10.66 | 5074.11404 | 1 |
| A925* | 4.75 | 448.9205 | 2 |
| A925L* | 4.75 | 448.9205 | 2 |
| A925LPE3 | 4.75 | 448.9205 | 2 |
| H1299 | 19.88 | 32910.7477 | 3 |
| H1650 | 2.33 | 52.9854189 | 4 |
| H1975 | 11.03 | 5621.02021 | 5 |
| H1993 | 8.15 | 2267.57 | 6 |
| H292 | 4.03 | 274.159783 | 7 |
| H3122 | 8.5 | 2572.44078 | 8 |
| H4006 | 10.39 | 4698.24058 | 9 |
| H460 | 13.7 | 10770.8583 | 10 |
| H661 | 5.67 | 763.550535 | 11 |
| HCC2935 | 3.528 | 183.94 | 12 |
| HCC4006 | 1.88 | 27.833137 | 13 |
| HCC78 | 13.73 | 10841.7708 | 14 |
| HCC827 | 13.55 | 10420.9304 | 15 |
| lc-2 ad | 8.91 | 2962.93225 | 16 |
| PC9 | 8.16 | 2275.93097 | 17 |

Table S5: Summary of NSCLC cell line measurements. The final measurement is the measurement for one cell and thus will be multiplied by the total number of cells in each well to give the estimated volume for the cell viability analysis. *It is assumed that A925L and A925 are the same sizes as A925LPE3. The estimated tumour volume is based on the volume of a sphere.

Note: The data presented is based on the data extracted from these images using ImageJ (<https://imagej.net/ij/index.html>). The reference number refers to the original source for the electron micrographs:

1. (ATCC) American Type Culture Collection, A549. <<https://www.atcc.org/products/ccl-185#detailed-product-images>> (2023). Accessed 15 June 2023.
2. Taniguchi H, et al. Amphiregulin triggered epidermal growth factor receptor activation confersin vivocrizotinib-resistance of EML4-ALK lung cancer and circumvention by epidermal growth factor receptor inhibitors. *Cancer Science*. 108(1):53-60 (2016).
3. (ATCC) American Type Culture Collection, H1299. <<https://www.atcc.org/products/crl-5803#detailed-product-images>> (2023) Accessed 15 June 2023.
4. Duan L, et al. VEGFC/VEGFR3 axis mediates TGFβ1-induced epithelial-to-mesenchymal transition in non-small cell lung cancer cells. *PLOS ONE*. 13(7):e0200452 (2018).
5. (ATCC) American Type Culture Collection, H1975. <<https://www.atcc.org/products/crl-5908#detailed-product-images>> (2023). Accessed 15 June 2023.
6. Cai J, Huang J, Wang W, Zeng J, Wang P. miR-124-3p Regulates FGF2–EGFR Pathway to Overcome Pemetrexed Resistance in Lung Adenocarcinoma Cells by Targeting MGAT5. *Cancer Management and Research*. 12:11597-11609 (2020).
7. Dabbagh K, Takeyama K, Lee H,  Ueki IF, Lausier JA, Nadel JA. IL-4 Induces Mucin Gene Expression and Goblet Cell Metaplasia In Vitro and In Vivo. *The Journal of Immunology*. 162 (10) 6233-6237 (1999).
8. Gower A, Hsu W, Hsu S, Wang Y, Giaccone G. EMT is associated with, but does not drive resistance to ALK inhibitors among EML4-ALK non-small cell lung cancer. *Molecular Oncology*. 10(4):601-609 (2015).
9. Zhang W, Cai X, Yu J, Lu X, Qian Q, Qian W. Exosome-mediated transfer of lncRNA RP11‑838N2.4 promotes erlotinib resistance in non-small cell lung cancer*. International Journal of Oncology*. (2018).
10. Hao S, et al. The In Vitro Anti-Tumor Activity of Phycocyanin against Non-Small Cell Lung Cancer Cells. *Marine Drugs*. 16(6):178 (2018).
11. Xu Y, et al. Mutated p53 Promotes the Symmetric Self-Renewal of Cisplatin-Resistant Lung Cancer Stem-Like Cells and Inhibits the Recruitment of Macrophages. *Journal of Immunology Research*. 2019:1-9 (2019).
12. (ATCC) American Type Culture Collection, HCC2935. <<https://www.atcc.org/products/crl-2869?geo_country=gb#detailed-product-images>> (2023). Accessed 15 June 2023.
13. Lai M. Opportunity for Pharmaceutical Intervention in Lung Cancer: Selective Inhibition of JAK1/2 to Eliminate EMT-Derived Mesenchymal Cells. *Journal of Young Investigators*. 31(5) (2016).
14. Song A, et al. Molecular Changes Associated with Acquired Resistance to Crizotinib in ROS1-Rearranged Non–Small Cell Lung Cancer. *Clinical Cancer Research*. 21(10):2379-2387 (2015).
15. (ATCC) American Type Culture Collection, HCC827. <<https://www.atcc.org/products/crl-2868?geo_country=gb#detailed-product-images>> (2023). Accessed 15 June 2023.
16. Cell Bank, RCB0440: LC-2 ad. <<https://cellbank.brc.riken.jp/cell_bank/CellInfo/?cellNo=RCB0440>> (2023). Accessed 15 June 2023.

1. Wang H, Hsu M, Wang K, Tseng C, Chen F, Hsu JT. Non-small-cell lung cancer cells combat epidermal growth factor receptor tyrosine kinase inhibition through immediate adhesion-related responses. *OncoTargets and Therapy*. 2016:2961-2973 (2016).

**Structural identifiability analysis for NLME model**

Below shows Eqn(6) (main article) reinterpreted for a NLME model context;

|  | $\left\{ \begin{aligned} \dot{x}_{i}(t,\psi_{i})=f(x_{i}(t,\psi_{i}),\psi_{i},\psi(t)) \\ y_{i}\left( t,\psi_{i} \right)=g(x_{i}\left( t,\psi_{i} \right),\psi_{i},\psi\left( t \right)) \\ x_{i}\left( 0 \right)=x_{o}(\psi_{i}) \end{aligned} \right.$ | (S.1) |
| --- | --- | --- |

where $\psi_{i}$ represented a function which consists of population effects (parameters) *θ* and the random effects which are usually defined as a normal distribution with a mean of 0 and $\Omega$ (the covariance matrix of the random effects). Inside the matrix the random effects themselves are usually represented as $\omega_{i}$. Given that the effects (both population and random) from NLME models originate form a distribution, the previous concept of structural identifiability can be expanded to include distributions. As an example, $\psi_{i}$ is said to be globally identifiabile if a unique distribution can be determined from the output distribution $y_{i}\left( t,\psi_{i} \right)$. Note that $y_{i}\left( t,\psi_{i} \right)$ cab ve represented as $p\left( y_{\{\theta,\Omega\}},t \right)$. Therefore in order for the NLME model to be classed as at-least locally identifiable, *θ* and $\Omega$ will have to be at-least locally identifiable.

Using the Taylor Series as well as the input-output approach, the coefficients from the Taylor series, as well as the monomial coefficients from the input-output approach, will generate unique solutions. These solutions can be represented as $Z\left( \theta,\eta\right)$. Thus, the statistical moments and the covariances of $Z\left( \theta,\eta\right)$ can be used to assess the structural identifiability of the NLME model.

For the sake of brevity the *in-vitro* TKI model will be represented as follows;

| $\frac{d\left( {TKI}_{M} \right)}{dt}= -\left( \theta_{1}\times{TKI}_{M} \right)+\left( \frac{\theta_{2}}{\frac{\theta_{3}}{(\theta_{4}\times(\frac{TumourCellViabilityCellGrowth}{TumourCellViabilityCellGrowth\left( 0 \right)})}} \right)\times{TKI}_{C}$ | (S.2) |
| --- | --- |
| $\frac{d({TKI}_{C})}{dt}=\left( \frac{\theta_{1}}{\frac{\theta_{4}\times(\frac{TumourCellViabilityCellGrowth}{TumourCellViabilityCellGrowth\left( 0 \right)})}{\theta_{3}}} \right)\times{TKI}_{M}-(\theta_{2}\times{TKI}_{C})$ | (S.3) |
| $\frac{d\left( TumourCellViability \right)}{dt}=\theta_{5}\times\left( 1-\frac{\theta_{6}\times{TKI}_{C}}{\theta_{7}+{TKI}_{C}} \right) -\theta_{8}\times TumourCellViability$ | (S.4) |

where $\theta_{x}$ represents the parameters of the model and *TKI_M_*_,_ *TKI_C_* and *TumourCellviability* are the model outputs. Note that $\theta_{4},\theta_{6},\theta_{7}$ represent *V_C_, I_MAX_* and *IC_50_* and are known values. The input-output approach was able to generate the following monomial coefficients for the model with *TumourCellViability* as the observed state.

$$\phi_{1}=\theta_{2}$$

$$\phi_{2}=\theta_{2}\theta_{8}^{3}$$

$$\phi_{3}=\theta_{5}\theta_{6}$$

$$\phi_{4}=\theta_{5}\theta_{6}\theta_{8}$$

$$\phi_{5}=3\theta_{2}\theta_{8}$$

$$\phi_{6}= 3\theta_{2}\theta_{8}^{2}$$

$$\phi_{7}= -2\theta_{5}\theta_{6}$$

$$\phi_{8}= -\theta_{5}^{2}\theta_{6}+\theta_{5}^{2}\theta_{6}^{2}$$

$$\phi_{9}= -\theta_{2}\theta_{5}^{3}+2\theta_{2}\theta_{5}^{3}\theta_{6}-\theta_{2}\theta_{5}^{3}\theta_{6}^{2}$$

$$\phi_{10}= -3\theta_{2}\theta_{5}+2\theta_{2}\theta_{5}\theta_{6}+\theta_{5}\theta_{6}\theta_{8}$$

$$\phi_{11}= \theta_{1}\theta_{5}\theta_{6}+\theta_{2}\theta_{5}\theta_{6}-2\theta_{5}\theta_{6}\theta_{8}$$

$$\phi_{12}= \theta_{1}\theta_{5}\theta_{6}\theta_{8}+\theta_{2}\theta_{5}\theta_{6}\theta_{8}+\theta_{5}\theta_{6}\theta_{8}^{2}$$

$$\phi_{13}= -3\theta_{2}\theta_{5}\theta_{8}^{2}+\theta_{1}\theta_{5}\theta_{6}\theta_{8}^{2}+3\theta_{2}\theta_{5}\theta_{6}\theta_{8}^{2}$$

$$\phi_{14}= -6\theta_{2}\theta_{5}\theta_{8}+\theta_{1}\theta_{5}\theta_{6}\theta_{8}+5\theta_{2}\theta_{5}\theta_{6}\theta_{8}-\theta_{5}\theta_{6}\theta_{8}^{2}$$

$$\phi_{15}= 3\theta_{2}\theta_{5}^{2}-4\theta_{2}\theta_{5}^{2}\theta_{6}+\theta_{2}\theta_{5}^{2}\theta_{6}^{2}-\theta_{5}^{2}\theta_{6}\theta_{8}+\theta_{5}^{2}\theta_{6}^{2}\theta_{8}$$

$$\phi_{16}= 3\theta_{2}\theta_{5}^{2}\theta_{8}-\theta_{1}\theta_{5}^{2}\theta_{6}\theta_{8}-5\theta_{2}\theta_{5}^{2}\theta_{6}\theta_{8}+\theta_{1}\theta_{5}^{2}\theta_{6}^{2}\theta_{8}+2\theta_{2}\theta_{5}^{2}\theta_{6}^{2}\theta_{8}$$

$$\phi_{17}= -\theta_{1}\theta_{5}^{2}\theta_{6}-\theta_{2}\theta_{5}^{2}\theta_{6}+\theta_{1}\theta_{5}^{2}\theta_{6}^{2}+\theta_{2}\theta_{5}^{2}\theta_{6}^{2}-\theta_{5}^{2}\theta_{6}\theta_{8}+\theta_{5}^{2}\theta_{6}^{2}\theta_{8}$$

The Taylor Series approach generated the following coefficients based on *TumourCellViability* as the observed output:

$$\phi_{18}= \frac{\theta_{5}\theta_{7}-100\theta_{7}\theta_{8}}{\theta_{7}}$$

$$\phi_{19}= -\frac{\theta_{8}(\theta_{5}\theta_{7}-100\theta_{7}\theta_{8})}{\theta_{7}}+\frac{\mathrm{Dose}\theta_{1}\theta_{4}(\frac{\theta_{5}-\theta_{5}\theta_{6}-100\theta_{8}}{\theta_{7}}-\frac{\theta_{5}\theta_{7}-100\theta_{7}\theta_{8}}{\theta_{7}^{2}})}{\theta_{3}}$$

Note that *Dose* is a known parameter. The coefficients will need to be transformed in order to take into account the mixed effects parameters. The corresponding vector for the unknown fixed parameters consists of: $\theta_{1},\theta_{2},\theta_{3},\theta_{5}$ and $\theta_{8}$ ($\theta_{4},\theta_{6},\theta_{7}$ represent *V_C_, I_MAX_* and *IC_50_*). The unknown random effects are present in the covariance matrix:

|  | $\Omega=\left( \begin{matrix} \omega_{1} & \omega_{12} & \omega_{13} & \omega_{15} & \omega_{18} \\ \omega_{21} & \omega_{2} & \omega_{23} & \omega_{25} & \omega_{28} \\ \omega_{31} & \omega_{32} & \omega_{3} & \omega_{35} & \omega_{38} \\ \omega_{51} & \omega_{52} & \omega_{53} & \omega_{5} & \omega_{58} \\ \omega_{81} & \omega_{82} & \omega_{83} & \omega_{85} & \omega_{8} \end{matrix} \right)$ | (S.5) |
| --- | --- | --- |

Note that $\omega_{12}$ would be the same as $\omega_{21}$. Therefore, based on the population parameters and the random effects, there are 20 parameters in total that need to be identifiable in order for the model to be classed as structurally identifiable. For the sake of brevity, the transformations of the coefficients are not reported here however the moments will be presented below.

Moments of the transformed coefficients

$$Z_{1}= ⅇ^{\frac{\omega_{2}}{2}}\theta_{2}$$

$$Z_{1}^{2}= ⅇ^{2\omega_{2}}\theta_{2}^{2}$$

$$Z_{2}= ⅇ^{\frac{1}{2}(\omega_{2}+9\omega_{8}+6\omega_{28})}\theta_{2}\theta_{8}^{3}$$

$$Z_{2}^{2}= ⅇ^{2(\omega_{2}+9\omega_{8}+6\omega_{28})}\theta_{2}^{2}\theta_{8}^{6}$$

$$Z_{3}= ⅇ^{\frac{\omega_{5}}{2}}\theta_{5}\theta_{6}$$

$$Z_{3}^{2}= ⅇ^{2\omega_{5}}\theta_{5}^{2}\theta_{6}^{2}$$

$$Z_{4}= ⅇ^{\frac{1}{2}(\omega_{5}+\omega_{8}+2\omega_{58})}\theta_{5}\theta_{6}\theta_{8}$$

$$Z_{4}^{2}= ⅇ^{2(\omega_{5}+\omega_{8}+2\omega_{58})}\theta_{5}^{2}\theta_{6}^{2}\theta_{8}^{2}$$

$$Z_{5}= 3ⅇ^{\frac{1}{2}(\omega_{2}+\omega_{8}+2\omega_{28})}\theta_{2}\theta_{8}$$

$$Z_{5}^{2}= 9ⅇ^{2(\omega_{2}+\omega_{8}+2\omega_{28})}\theta_{2}^{2}\theta_{8}^{2}$$

$$Z_{6}= 3ⅇ^{\frac{1}{2}(\omega_{2}+4(\omega_{8}+\omega_{28}))}\theta_{2}\theta_{8}^{2}$$

$$Z_{6}^{2}= 9ⅇ^{2(\omega_{2}+4(\omega_{8}+\omega_{28}))}\theta_{2}^{2}\theta_{8}^{4}$$

$$Z_{7}= -2ⅇ^{\frac{\omega_{5}}{2}}\theta_{5}\theta_{6}$$

$$Z_{7}^{2}= 4ⅇ^{2\omega_{5}}\theta_{5}^{2}\theta_{6}^{2}$$

$$Z_{8}= ⅇ^{2\omega_{5}}\theta_{5}^{2}(-1+\theta_{6})\theta_{6}$$

$$Z_{8}^{2}= ⅇ^{8\omega_{5}}\theta_{5}^{4}{(-1+\theta_{6})}^{2}\theta_{6}^{2}$$

$$Z_{9}= -ⅇ^{\frac{1}{2}(\omega_{2}+9\omega_{5}+6\omega_{25})}\theta_{2}\theta_{5}^{3}{(-1+\theta_{6})}^{2}$$

$$Z_{9}^{2}= ⅇ^{2(\omega_{2}+9\omega_{5}+6\omega_{25})}\theta_{2}^{2}\theta_{5}^{6}{(-1+\theta_{6})}^{4}$$

$$Z_{10}= ⅇ^{\frac{\omega_{5}}{2}}\theta_{5}(ⅇ^{\frac{\omega_{2}}{2}+\omega_{25}}\theta_{2}(-3+2\theta_{6})+ⅇ^{\frac{\omega_{8}}{2}+\omega_{58}}\theta_{6}\theta_{8})$$

$$Z_{10}^{2}= ⅇ^{2\omega_{5}}\theta_{5}^{2}(ⅇ^{2\omega_{2}+4\omega_{25}}\theta_{2}^{2}{(3-2\theta_{6})}^{2}+2ⅇ^{\frac{1}{2}(\omega_{2}+\omega_{8}+4\omega_{25}+2\omega_{28}+4\omega_{58})}\theta_{2}\theta_{6}(-3+2\theta_{6})\theta_{8}+ⅇ^{2\omega_{8}+4\omega_{58}}\theta_{6}^{2}\theta_{8}^{2})$$

$$Z_{11}= ⅇ^{\frac{\omega_{5}}{2}}\theta_{5}\theta_{6}(ⅇ^{\frac{\omega_{1}}{2}+\omega_{15}}\theta_{1}+ⅇ^{\frac{\omega_{2}}{2}+\omega_{25}}\theta_{2}-2ⅇ^{\frac{\omega_{8}}{2}+\omega_{58}}\theta_{8})$$

$$Z_{11}^{2}= ⅇ^{2\omega_{5}}\theta_{5}^{2}\theta_{6}^{2}(ⅇ^{2\omega_{1}+4\omega_{15}}\theta_{1}^{2}+ⅇ^{2\omega_{2}+4\omega_{25}}\theta_{2}^{2}-4ⅇ^{\frac{1}{2}(\omega_{2}+\omega_{8}+4\omega_{25}+2\omega_{28}+4\omega_{58})}\theta_{2}\theta_{8}+4ⅇ^{2\omega_{8}+4\omega_{58}}\theta_{8}^{2}+2ⅇ^{\frac{\omega_{1}}{2}+2\omega_{15}}\theta_{1}(ⅇ^{\frac{\omega_{2}}{2}+\omega_{12}+2\omega_{25}}\theta_{2}-2ⅇ^{\frac{\omega_{8}}{2}+\omega_{18}+2\omega_{58}}\theta_{8}))$$

$$Z_{12}= ⅇ^{\frac{1}{2}(\omega_{5}+\omega_{8}+2\omega_{58})}\theta_{5}\theta_{6}\theta_{8}(ⅇ^{\frac{\omega_{1}}{2}+\omega_{15}+\omega_{18}}\theta_{1}+ⅇ^{\frac{\omega_{2}}{2}+\omega_{25}+\omega_{28}}\theta_{2}+ⅇ^{\frac{3\omega_{8}}{2}+\omega_{58}}\theta_{8})$$

$$Z_{12}^{2}= ⅇ^{2(\omega_{5}+\omega_{8}+2\omega_{58})}\theta_{5}^{2}\theta_{6}^{2}\theta_{8}^{2}(ⅇ^{2(\omega_{1}+2(\omega_{15}+\omega_{18}))}\theta_{1}^{2}+ⅇ^{2(\omega_{2}+2(\omega_{25}+\omega_{28}))}\theta_{2}^{2}+2ⅇ^{\frac{1}{2}(\omega_{2}+5\omega_{8}+4\omega_{25}+6\omega_{28}+4\omega_{58})}\theta_{2}\theta_{8}+ⅇ^{6\omega_{8}+4\omega_{58}}\theta_{8}^{2}+2ⅇ^{\frac{1}{2}(\omega_{1}+4(\omega_{15}+\omega_{18}))}\theta_{1}(ⅇ^{\frac{\omega_{2}}{2}+\omega_{12}+2(\omega_{25}+\omega_{28})}\theta_{2}+ⅇ^{\frac{5\omega_{8}}{2}+\omega_{18}+2\omega_{58}}\theta_{8}))$$

$$Z_{13}= ⅇ^{\frac{1}{2}(\omega_{5}+4(\omega_{8}+\omega_{58}))}\theta_{5}(3ⅇ^{\frac{\omega_{2}}{2}+\omega_{25}+2\omega_{28}}\theta_{2}(-1+\theta_{6})+ⅇ^{\frac{\omega_{1}}{2}+\omega_{15}+2\omega_{18}}\theta_{1}\theta_{6})\theta_{8}^{2}$$

$$Z_{13}^{2}= ⅇ^{2(\omega_{5}+4(\omega_{8}+\omega_{58}))}\theta_{5}^{2}(9ⅇ^{2\omega_{2}+4\omega_{25}+8\omega_{28}}\theta_{2}^{2}{(-1+\theta_{6})}^{2}+6ⅇ^{\frac{1}{2}(\omega_{1}+\omega_{2}+2\omega_{12}+4\omega_{15}+8\omega_{18}+4\omega_{25}+8\omega_{28})}\theta_{1}\theta_{2}(-1+\theta_{6})\theta_{6}+ⅇ^{2\omega_{1}+4\omega_{15}+8\omega_{18}}\theta_{1}^{2}\theta_{6}^{2})\theta_{8}^{4}$$

$$Z_{14}= -ⅇ^{\frac{1}{2}(\omega_{5}+\omega_{8}+2\omega_{58})}\theta_{5}\theta_{8}(-ⅇ^{\frac{\omega_{2}}{2}+\omega_{25}+\omega_{28}}\theta_{2}(-6+5\theta_{6})+\theta_{6}(-ⅇ^{\frac{\omega_{1}}{2}+\omega_{15}+\omega_{18}}\theta_{1}+ⅇ^{\frac{3\omega_{8}}{2}+\omega_{58}}\theta_{8}))$$

$$Z_{14}^{2}= ⅇ^{2(\omega_{5}+\omega_{8}+2\omega_{58})}\theta_{5}^{2}\theta_{8}^{2}(ⅇ^{2(\omega_{2}+2(\omega_{25}+\omega_{28}))}\theta_{2}^{2}{(6-5\theta_{6})}^{2}-2ⅇ^{\frac{1}{2}(\omega_{2}+4(\omega_{25}+\omega_{28}))}\theta_{2}\theta_{6}(-6+5\theta_{6})(-ⅇ^{\frac{\omega_{1}}{2}+\omega_{12}+2(\omega_{15}+\omega_{18})}\theta_{1}+ⅇ^{\frac{5\omega_{8}}{2}+\omega_{28}+2\omega_{58}}\theta_{8})+\theta_{6}^{2}(ⅇ^{2(\omega_{1}+2(\omega_{15}+\omega_{18}))}\theta_{1}^{2}-2ⅇ^{\frac{1}{2}(\omega_{1}+5\omega_{8}+4\omega_{15}+6\omega_{18}+4\omega_{58})}\theta_{1}\theta_{8}+ⅇ^{6\omega_{8}+4\omega_{58}}\theta_{8}^{2}))$$

$$Z_{15}= ⅇ^{2\omega_{5}}\theta_{5}^{2}(-1+\theta_{6})(ⅇ^{\frac{\omega_{2}}{2}+2\omega_{25}}\theta_{2}(-3+\theta_{6})+ⅇ^{\frac{\omega_{8}}{2}+2\omega_{58}}\theta_{6}\theta_{8})$$

$$Z_{15}^{2}= ⅇ^{8\omega_{5}}\theta_{5}^{4}{(-1+\theta_{6})}^{2}(ⅇ^{2\omega_{2}+8\omega_{25}}\theta_{2}^{2}{(-3+\theta_{6})}^{2}+2ⅇ^{\frac{1}{2}(\omega_{2}+\omega_{8}+8\omega_{25}+2\omega_{28}+8\omega_{58})}\theta_{2}(-3+\theta_{6})\theta_{6}\theta_{8}+ⅇ^{2\omega_{8}+8\omega_{58}}\theta_{6}^{2}\theta_{8}^{2})$$

$$Z_{16}= ⅇ^{2\omega_{5}+\frac{\omega_{8}}{2}+2\omega_{58}}\theta_{5}^{2}(-1+\theta_{6})(ⅇ^{\frac{\omega_{1}}{2}+2\omega_{15}+\omega_{18}}\theta_{1}\theta_{6}+ⅇ^{\frac{\omega_{2}}{2}+2\omega_{25}+\omega_{28}}\theta_{2}(-3+2\theta_{6}))\theta_{8}$$

$$Z_{16}^{2}= ⅇ^{8\omega_{5}+2\omega_{8}+8\omega_{58}}\theta_{5}^{4}{(-1+\theta_{6})}^{2}(ⅇ^{2\omega_{2}+8\omega_{25}+4\omega_{28}}\theta_{2}^{2}{(3-2\theta_{6})}^{2}+ⅇ^{2\omega_{1}+8\omega_{15}+4\omega_{18}}\theta_{1}^{2}\theta_{6}^{2}+2ⅇ^{\frac{1}{2}(\omega_{1}+\omega_{2}+2\omega_{12}+8\omega_{15}+4\omega_{18}+8\omega_{25}+4\omega_{28})}\theta_{1}\theta_{2}\theta_{6}(-3+2\theta_{6}))\theta_{8}^{2}$$

$$Z_{17}= ⅇ^{2\omega_{5}}\theta_{5}^{2}(-1+\theta_{6})\theta_{6}(ⅇ^{\frac{\omega_{1}}{2}+2\omega_{15}}\theta_{1}+ⅇ^{\frac{\omega_{2}}{2}+2\omega_{25}}\theta_{2}+ⅇ^{\frac{\omega_{8}}{2}+2\omega_{58}}\theta_{8})$$

$$Z_{17}^{2}= ⅇ^{8\omega_{5}}\theta_{5}^{4}{(-1+\theta_{6})}^{2}\theta_{6}^{2}(ⅇ^{2\omega_{1}+8\omega_{15}}\theta_{1}^{2}+ⅇ^{2\omega_{2}+8\omega_{25}}\theta_{2}^{2}+2ⅇ^{\frac{1}{2}(\omega_{2}+\omega_{8}+8\omega_{25}+2\omega_{28}+8\omega_{58})}\theta_{2}\theta_{8}+ⅇ^{2\omega_{8}+8\omega_{58}}\theta_{8}^{2}+2ⅇ^{\frac{\omega_{1}}{2}+4\omega_{15}}\theta_{1}(ⅇ^{\frac{\omega_{2}}{2}+\omega_{12}+4\omega_{25}}\theta_{2}+ⅇ^{\frac{\omega_{8}}{2}+\omega_{18}+4\omega_{58}}\theta_{8}))$$

$$Z_{18}= ⅇ^{\frac{\omega_{5}}{2}}\theta_{5}-100ⅇ^{\frac{\omega_{8}}{2}}\theta$$

$$Z_{18}^{2}= ⅇ^{2\omega_{5}}\theta_{5}^{2}-200ⅇ^{\frac{1}{2}(\omega_{5}+\omega_{8}+2\omega_{58})}\theta_{5}\theta_{8}+10000ⅇ^{2\omega_{8}}\theta_{8}^{2}$$

$$Z_{19}= -\frac{\mathrm{Dose}ⅇ^{\frac{1}{2}(\omega_{1}+\omega_{3}+\omega_{5}-2\omega_{13}+2\omega_{15}-2\omega_{35})}\theta_{1}\theta_{4}\theta_{5}\theta_{6}}{\theta_{3}\theta_{7}}-ⅇ^{\frac{1}{2}(\omega_{5}+\omega_{8}+2\omega_{58})}\theta_{5}\theta_{8}+100ⅇ^{2\omega_{8}}\theta_{8}^{2}$$

$$Z_{19}^{2}= \frac{1}{\theta_{3}^{2}\theta_{7}^{2}}ⅇ^{-2(2\omega_{13}+2\omega_{35}+\omega_{38})}(\mathrm{Dose}^{2}ⅇ^{2(\omega_{1}+\omega_{3}+\omega_{5}+2\omega_{15}+\omega_{38})}\theta_{1}^{2}\theta_{4}^{2}\theta_{5}^{2}\theta_{6}^{2}+2\mathrm{Dose}ⅇ^{\frac{1}{2}(\omega_{1}+\omega_{3}+\omega_{5}+\omega_{8}+6\omega_{13}+2\omega_{15}+2\omega_{18}+4\omega_{35}+4\omega_{58})}\theta_{1}\theta_{3}\theta_{4}\theta_{5}\theta_{6}\theta_{7}\theta_{8}(ⅇ^{\frac{3\omega_{5}}{2}+\omega_{15}+\omega_{38}}\theta_{5}-100ⅇ^{\frac{3\omega_{8}}{2}+\omega_{18}+\omega_{35}}\theta_{8})+ⅇ^{2(\omega_{8}+2\omega_{13}+2\omega_{35}+\omega_{38})}\theta_{3}^{2}\theta_{7}^{2}\theta_{8}^{2}(ⅇ^{2\omega_{5}+4\omega_{58}}\theta_{5}^{2}-200ⅇ^{\frac{1}{2}(\omega_{5}+5\omega_{8}+6\omega_{58})}\theta_{5}\theta_{8}+10000ⅇ^{6\omega_{8}}\theta_{8}^{2}))$$

Moments can also be multiplied with each other to create covariance for example:

$Z_{1}Z_{19}= -\frac{1}{\theta_{3}\theta_{7}}ⅇ^{\frac{1}{2}(\omega_{2}-2(\omega_{13}+\omega_{23}+\omega_{35}))}\theta_{2}(-\mathrm{Dose}ⅇ^{\frac{1}{2}(\omega_{1}+\omega_{3}+\omega_{5}+2\omega_{15})}(ⅇ^{\omega_{23}}-ⅇ^{\omega_{12}+\omega_{25}})\theta_{1}\theta_{4}\theta_{5}\theta_{6}+ⅇ^{\frac{\omega_{8}}{2}+\omega_{13}+\omega_{23}+\omega_{35}}\theta_{3}\theta_{7}\theta_{8}(ⅇ^{\frac{\omega_{5}}{2}+\omega_{58}}(-1+ⅇ^{\omega_{25}+\omega_{28}})\theta_{5}-100ⅇ^{\frac{3\omega_{8}}{2}}(-1+ⅇ^{2\omega_{28}})\theta_{8}))$

$$Z_{2}Z_{19}= -\frac{\mathrm{Dose}ⅇ^{\frac{1}{2}(\omega_{1}+\omega_{2}+\omega_{3}+\omega_{5}+2\omega_{12}-2\omega_{13}+2\omega_{15}+3\omega_{18}-2\omega_{23}+2\omega_{25}+3\omega_{28}-2\omega_{35}-3\omega_{38}+3\omega_{58}+3(3\omega_{8}+\omega_{18}+\omega_{28}-\omega_{38}+\omega_{58}))}\theta_{1}\theta_{2}\theta_{4}\theta_{5}\theta_{6}\theta_{8}^{3}}{\theta_{3}\theta_{7}}-ⅇ^{\frac{1}{2}(\omega_{2}+\omega_{5}+2\omega_{25}+4\omega_{28}+4\omega_{58}+4(4\omega_{8}+\omega_{28}+\omega_{58}))}\theta_{2}\theta_{5}\theta_{8}^{4}+100ⅇ^{\frac{1}{2}(\omega_{2}+5\omega_{28}+5(5\omega_{8}+\omega_{28}))}\theta_{2}\theta_{8}^{5}$$

$$Z_{11}Z_{19}= \frac{1}{\theta_{3}\theta_{7}}ⅇ^{\frac{\omega_{5}}{2}}\theta_{5}\theta_{6}(\mathrm{Dose}ⅇ^{\frac{1}{2}(2\omega_{1}+\omega_{3}+\omega_{5}-4\omega_{13}+4\omega_{15}-4\omega_{35})}(-ⅇ^{\omega_{1}+\omega_{5}+2\omega_{15}}+ⅇ^{\omega_{13}+\omega_{35}})\theta_{1}^{2}\theta_{4}\theta_{5}\theta_{6}+ⅇ^{\frac{\omega_{8}}{2}}\theta_{3}\theta_{7}\theta_{8}(2ⅇ^{\frac{1}{2}(\omega_{5}+\omega_{8}+4\omega_{58})}(-1+ⅇ^{\omega_{5}+\omega_{8}+2\omega_{58}})\theta_{5}\theta_{8}-200ⅇ^{2\omega_{8}+\omega_{58}}(-1+ⅇ^{2(\omega_{8}+\omega_{58})})\theta_{8}^{2}-ⅇ^{\frac{\omega_{2}}{2}+\omega_{25}}\theta_{2}(ⅇ^{\frac{\omega_{5}}{2}+\omega_{58}}(-1+ⅇ^{\omega_{5}+\omega_{25}+\omega_{28}+\omega_{58}})\theta_{5}-100ⅇ^{\frac{3\omega_{8}}{2}}(-1+ⅇ^{2(\omega_{28}+\omega_{58})})\theta_{8}))+ⅇ^{\frac{\omega_{1}}{2}-\omega_{13}+\omega_{15}-\omega_{23}-2\omega_{35}-\omega_{38}}\theta_{1}(-\mathrm{Dose}ⅇ^{\frac{1}{2}(\omega_{2}+\omega_{3}+\omega_{5}+2\omega_{25}+2\omega_{38})}(ⅇ^{\omega_{5}+\omega_{12}+\omega_{15}+\omega_{25}}-ⅇ^{\omega_{23}+\omega_{35}})\theta_{2}\theta_{4}\theta_{5}\theta_{6}+ⅇ^{\frac{\omega_{8}}{2}+\omega_{23}}\theta_{8}(2\mathrm{Dose}ⅇ^{\frac{1}{2}(\omega_{3}+\omega_{5}+2\omega_{58})}(-ⅇ^{\omega_{35}+\omega_{38}}+ⅇ^{\omega_{5}+\omega_{15}+\omega_{18}+\omega_{58}})\theta_{4}\theta_{5}\theta_{6}-ⅇ^{\omega_{13}+2\omega_{35}+\omega_{38}}\theta_{3}\theta_{7}(ⅇ^{\frac{\omega_{5}}{2}+\omega_{58}}(-1+ⅇ^{\omega_{5}+\omega_{15}+\omega_{18}+\omega_{58}})\theta_{5}-100ⅇ^{\frac{3\omega_{8}}{2}}(-1+ⅇ^{2(\omega_{18}+\omega_{58})})\theta_{8}))))$$

Assessing parameters for identifiability

- From $Z_{1}$ and $Z_{1}^{2}$, $\theta_{2}$ and $\omega_{2}$ can be identified
- $Z_{3}$ and $Z_{7}$ can be used to identify $\theta_{5}$ and $\omega_{5}$
- $Z_{2}$, $Z_{2}^{2}$ and $Z_{5}$ can be used to identify $\theta_{8}$, $\omega_{28}$ and $\omega_{8}$
- Using the above solution, from $Z_{4}$ , $\omega_{58}$ can be identified
- From $Z_{9}$ , $\omega_{25}$ can be identified
- Solving $Z_{11}$ for $\omega_{1}$ and substituting into $Z_{12}$ identifies $\omega_{18}$
- Doing the same substitution in $Z_{17}$ identifies $\omega_{15}$
- $\theta_{1}$, $\omega_{1}$ and $\omega_{12}$ can be identified using $Z_{13}^{2}$ to solve for $\omega_{12}$. Then use $Z_{11}^{2}$ with the new substitution of $\omega_{12}$ and use $Z_{13}$ to solve for $\omega_{1}$ and identify $\theta_{1}$. From this $\omega_{1}$ and $\omega_{12}$ can also be identified
- Using $Z_{19}$ to solve for $\omega_{3}$ which can be substituted into $Z_{1}Z_{19}$ to identify $\omega_{23}$
- Doing the same with $Z_{3}Z_{19}$ will identify $\omega_{35}$
- Using $Z_{2}Z_{19}$ to solve for $\omega_{38}$ and substituting into $Z_{19}^{2}$ will identify $\theta_{3}$
- Using the previous solution and substituting into $Z_{11}Z_{19}$ will identify $\omega_{13}$
- Now from $Z_{19}$, $\omega_{3}$ can be identified and thus all of the parameters have been identified and the model is structurally identifiable.
